# Simulating the Impact of Improved Cardiovascular Risk Interventions on Clinical and Economic Outcomes in Russia

**DOI:** 10.1371/journal.pone.0103280

**Published:** 2014-08-20

**Authors:** Kenny Shum, Peter Alperin, Svetlana Shalnova, Sergey Boytsov, Anna Kontsevaya, Alexey Vigdorchik, Adam Guetz, Jennifer Eriksson, David Hughes

**Affiliations:** 1 Archimedes, San Francisco, California, United States of America; 2 National Research Center for Preventive Medicine, Moscow, Russia; 3 Novartis Pharma LLC, Moscow, Russia; 4 OptumInsight Life Sciences, Stockholm, Sweden; 5 Department of Healthcare Systems, Novartis International AG, Basel, Switzerland; The University of Auckland, New Zealand

## Abstract

**Objectives:**

Russia faces a high burden of cardiovascular disease. Prevalence of all cardiovascular risk factors, especially hypertension, is high. Elevated blood pressure is generally poorly controlled and medication usage is suboptimal. With a disease-model simulation, we forecast how various treatment programs aimed at increasing blood pressure control would affect cardiovascular outcomes. In addition, we investigated what additional benefit adding lipid control and smoking cessation to blood pressure control would generate in terms of reduced cardiovascular events. Finally, we estimated the direct health care costs saved by treating fewer cardiovascular events.

**Methods:**

The Archimedes Model, a detailed computer model of human physiology, disease progression, and health care delivery was adapted to the Russian setting. Intervention scenarios of achieving systolic blood pressure control rates (defined as systolic blood pressure <140 mmHg) of 40% and 60% were simulated by modifying adherence rates of an antihypertensive medication combination and compared with current care (23.9% blood pressure control rate). Outcomes of major adverse cardiovascular events; cerebrovascular event (stroke), myocardial infarction, and cardiovascular death over a 10-year time horizon were reported. Direct health care costs of strokes and myocardial infarctions were derived from official Russian statistics and tariff lists.

**Results:**

To achieve systolic blood pressure control rates of 40% and 60%, adherence rates to the antihypertensive treatment program were 29.4% and 65.9%. Cardiovascular death relative risk reductions were 13.2%, and 29.6%, respectively. For the current estimated 43,855,000-person Russian hypertensive population, each control-rate scenario resulted in an absolute reduction of 1.0 million and 2.4 million cardiovascular deaths, and a reduction of 1.2 million and 2.7 million stroke/myocardial infarction diagnoses, respectively. Averted direct costs from current care levels ($7.6 billion [in United States dollars]) were $1.1 billion and $2.6 billion, respectively.

## Introduction

Russia faces a high burden of cardiovascular (CV) disease (CVD), which is the primary cause of mortality accounting for 57% of all deaths in the country [1]. The rates of CV mortality and morbidity in Russia are among the highest in Europe [2]. In particular, male adult mortality rates substantially exceed those of other countries with similar gross domestic product per capita [3]. For example, in Russia, only approximately 45% of 20-year-old men can expect to live until age 65, compared to 88% of same-aged males in Switzerland [4]. In addition, cardiovascular mortality is high in the working age population, especially in males [3], [5], [6]. The prevalence of hypertension, one of the most common risk factors for CVD, is estimated at 39.7% in the adult Russian population [7], and has remained relatively stable during the last 10 years. This prevalence is higher than that in other countries, including the United Kingdom (28% to 31%) [8], the United States (US; 29%) [9], France (31%) [10], and Canada (22%) [11].

Treatment and control of hypertension in Russia is less than optimal. According to the latest reports from a 10-year federal program evaluating the prevention and treatment of hypertension, few treated patients achieved blood pressure (BP) control (from 23% [beginning of study] to 24% [end of study]), despite a modest increase in treatment rate from 63% to 66% [7]. From 2009–2010, the most commonly prescribed hypertension treatments in Russia were angiotensin-converting enzyme (ACE) inhibitors (63% of patients), diuretics (37%), beta blockers (31%), calcium channel blockers (CCBs; 15%), and angiotensin receptor blockers (ARBs; 5%) [7]. A pharmacoepidemiologic study of hypertensive patients in clinical practices in Russia reported that approximately 26% of hypertensive patients are treated with monotherapy, 37% with two drugs and 37% with three or more drugs [12]. Data on statins use in Russia had large variability. From 2004 to 2009, use of statins in the population with ischemic heart disease increased from 10.0% to 85.5% [13]. In another study conducted between 2005 and 2007, only 1.9% of the high-risk patients were taking statins prior to an acute myocardial infarction [14]. Hence, data suggested that the use of statins for primary CVD prevention in the high-risk Russian population was low.

Reasons for suboptimal hypertensive management in Russia include additional comorbidities (eg, smoking, left ventricular hypertrophy, obesity, dyslipidemia,) [15], poor treatment adherence (eg, patients not taking their medication regularly) [16], [17], and nonadherence (eg, unwillingness to change smoking, diet, and exercise patterns or show up for appointments) [18]. ‘Therapeutic inertia’ of physicians also contributes to poor management of hypertension in Russia, with documented low rates of combination therapy and thiazide diuretics use [19], [20].

The economic burden of CVD in Russia is high. A recent study [6] estimated direct health care costs related to CVD in Russia (in 2009) of approximately US$7 billion. Coronary heart disease (CHD) accounted for 45.3% of total CVD direct health care costs (∼$3.1 billion), while total health care costs of cerebrovascular disease were estimated at $1.2 billion [6].

The purpose of this study was to assess the potential benefits of better hypertension treatment programs in Russia, with the intent of reducing CV mortality and morbidity in the hypertensive population. With a disease-model simulation, we forecast how various treatment programs aimed at increasing BP control would affect CV outcomes. Systolic blood pressure (SBP) control rate was used because of the high rate of SBP hypertension in Russia [15], the strong association between systolic hypertension and adverse cardiovascular outcomes [21], and to better align with available data. In addition, we investigated what additional benefit adding lipid control and smoking cessation to BP control would generate in terms of reduced CV events. Finally, we estimated the direct health care costs saved by treating fewer CV events.

## Methods

### Adaptation of the model

The Archimedes Model (the Model) is a trial-validated, clinically detailed simulation model of human physiology, disease progression, and health care delivery [22]. The core of the Model is a set of equations representing the physiological pathways pertinent to diseases and their complications. Use of the integrated Archimedes model enables a comparison of a wide range of treatments, guidelines, and performance measures within an integrated system, and to address comorbidities, syndromes that span multiple organ systems, drugs that have multiple effects, and combinations of treatments. The Model has been carefully developed over several years to predict adverse health outcome event rates and the effects of medical interventions on health outcomes. The structure of the model and data sources used in the modeling is described elsewhere [23] and is provided for reference in Appendix S1 in File S1. The CVD module of the Model has been calibrated and validated against a large number of publicly available summary- and individual-level data sets [24] (and see Appendix S2 in File S1).

Because the Model was primarily developed using patient data from the US and Western Europe, it had to be calibrated to allow it to capture the event rates observed in Russia. To do this, the underlying dynamics of disease onset related to important risk factors was assumed to be similar, but the baseline event rate was modified to match that observed in the Russian population. Data sources used for model calibrations were age-adjusted CHD mortality rate and age-adjusted cerebrovascular (hereafter referred to as ‘stroke’) mortality rate (unpublished data: see Appendix S3 in File S1). The Model was calibrated in the following way: (1) baseline event rates for myocardial infarction (MI), death after MI, other CHD death unrelated to MI, stroke, and death after stroke were adjusted so that both age-adjusted CHD mortality and stroke mortality rates in the Model matched those reported for Russia, and (2) the diagnosed/undiagnosed ratios of MI and of stroke were calibrated to better reflect a lower diagnosis rate of MI and stroke compared with the US, based on data from the Yaroslavl region of Russia (unpublished data: Appendix S4 in File S1) [25], [26]. The lower diagnosis ratios observed in Russia may, in part, be related to the lack of MI diagnosed in cases of death outside hospital, the failure to obtain care, or the lack of broad access to advanced diagnostic technologies. Other baseline characteristics of the Russian population including age distribution, gender distribution, and smoking status were controlled for. Baseline values for age, smoking status, prevalence of hypertension, and BP level and distribution in the hypertensive population were estimated using multiple sources [2], [7], [27]–[29] and imputed from the Model-based population. The Model was updated and adapted based on a recent model simulation of clinical outcomes in the Yaroslavl region of Russia (unpublished data, Archimedes/Novartis: white paper on model simulation).

### Intervention scenarios

The simulated hypertensive population in Russia was treated with an antihypertensive fixed-dose combination pill consisting of 3 components: an ARB, a CCB, and a diuretic. This combination of antihypertensive drug classes is included in the hypertension guideline management algorithm [30], [31]. The combination of an ARB, CCB, and diuretic has been shown to have greater proportion of patients achieving BP control (<140/90 mmHg) compared with dual therapies [32], [33]. The effect of the fixed-dose combination pill was modeled to have the same effect as taking each component separately, and its effects on cardiovascular outcomes were modeled as proportional to the reduction in SBP. This modeling approach is consistent with findings in a meta-analysis of antihypertensive trials [34]. Scenarios were generated in which different rates of SBP control (ie, SBP <140 mmHg) were achieved by modifying the adherence rates of the antihypertensive combination pill. The SBP control rates considered were 23.9% (baseline BP control rate in Russia) [7], 40%, and 60%. In each scenario, if a patient was assigned, and adhered, to the use of the combination pill, they discontinued all other usage of ACE-inhibitors/ARBs, CCBs, and diuretic medication. Otherwise, the patient was assigned medications according to the baseline scenario to achieve a 23.9% control rate. (Intervention scenarios to reach 30% and 50% SBP control were also generated, and these are presented in Table S1.) This simulation setup assumed that the SBP control rates are maintained for the full duration of the simulation. If one is interested in exploring the effect of decreasing SBP control rate over time, other simulation scenarios with lower SBP control rates can be used as lower bounds for the estimated effect on clinical outcomes.

In addition to improving the BP control rate, two other CV risk-reduction interventions were considered. First, the impact of improved dyslipidemia management by administering atorvastatin 20 mg together with the combination pill was assessed. This dosage was modeled to reduce low-density lipoprotein (LDL) cholesterol by 43% [35]. Fixed-dose medication bundles that include an antihypertensive, a statin, and aspirin have been shown to reduce CV outcomes [36]. In this simulation study, the potential impact of adding dyslipidemia treatment was estimated by assuming program participants adhered to the lipid-lowering medication the same amount as to their antihypertensive combination pill. If a patient was assigned, and adhered, to the use of atorvastatin, previous use of other statins as documented at baseline was discontinued. Second, the impact of a smoking cessation program in addition to increased BP control and improved dyslipidemia management was evaluated. Two smoking cessation program scenarios were considered: 25% and 50% reductions in smoking prevalence within the target hypertensive population maintained for 20 years. Hypertension treatment was evaluated first (before dyslipidemia and smoking) as hypertension is the CVD risk factor that is relatively easy to diagnose and treat. It is the number one risk factor for premature death [37] and is highly prevalent in Russia compared with other countries [38]. There is also a substantial amount of evidence that better hypertension control is correlated with better morbidity and mortality outcomes [30] and there are clear treatment guidelines in Russia [39]. Dyslipidemia treatment was added next. Smoking cessation was added last because, although it is a very large burden in Russia, it is extremely difficult to treat. This order is also consistent with results from the EURIKA study, which found hypertension to be the greatest risk factor in cardiovascular disease, followed by hyperlipidemia, and then smoking [40].

Intervention scenarios were also run in the 38% subpopulation of working-age patients: males 20–59 years and females 20–54 years of age (the upper limit is the statutory retirement age in Russia) [41].

### Clinical outcomes

A total of 100 000 virtual patients aged over 20 years and representative of the Russian hypertensive population were generated. The results were scaled to the 2010 Russian population. Based on the 2010 Russian population size [29] and the prevalence of hypertension in Russia [2], the estimated size of the hypertensive population was 44 million. Each simulated patient was run through the baseline control and 4 treatment scenarios and the following health outcomes were generated:

- Major adverse cardiac event (MACE; defined as the composite outcome of MI, stroke, or death due to CVD)
- Stroke diagnosis
- MI diagnosis
- CHD death (defined as fatal MI or other CHD death)
- Stroke death
- CVD death (defined as CHD death or stroke death)
- Life expectancy (which was computed by running the simulation for the full lifetime of each person [mean age at baseline was 61.1 years]).

### Economic outcomes

Direct health care costs related to treatment of MI and stroke events were calculated for each intervention scenario by multiplying a unit cost with each MI and stroke event. Direct health care costs attributable to the third-party payer were included. Costs (in US dollars) of MI ($1524/event) and stroke ($859/event) included hospitalizations, emergency care, and percutaneous coronary intervention/cardiosurgery (acute event) [6]. Follow-up costs, including outpatient visits and medications, were added for the first year after an MI ($328) and stroke ($53) event for patients surviving 30 days after the event. Total averted health care costs were calculated. Costs of the intervention program were not considered in this analysis. Costs were discounted at 3.5% per annum and presented in 2011 US dollars.

## Results

Baseline characteristics for the Russian hypertensive population simulated from the Archimedes Model are reported in Table 1. Demographic characteristics (ie, age and gender) and baseline risk factors of our population are generally consistent with that of the hypertensive Russian population [15]. To achieve an SBP control rate of 40% and 60%, the required adherence rate to the antihypertensive combination pill was estimated at 29.4% and 65.9%, respectively.

**Table 1:** Baseline characteristics for the Russian hypertensive population simulated from the Archimedes Model.

Baseline variable*	Male	Female
Gender	44	56
Mean age, years	57.0	64.2
Current smoker	52.3	15.0
Mean systolic blood pressure, mmHg	146	161
Mean diastolic blood pressure, mmHg	83	79
Mean LDL cholesterol, mg/dL	122	128
Mean HbA1c, %	5.8	5.6
History of coronary artery disease	10.8	7.9
History of myocardial infarction	4.3	2.0
History of cerebrovascular disease (stroke)	12.5	11.6
Type 2 diabetes	22.9	16.3
Dyslipidemia	62.0	49.3
Currently taking ACE inhibitor or ARB	19.3	16.3
Currently taking CCB	11.2	11.4
Currently taking diuretic	14.3	10.7
Currently taking statin	19.5	13.7

ACE, angiotensin-converting enzyme; ARB, angiotensin II receptor blocker; CCB, calcium channel blocker; HbA1c, hemoglobin A1c; LDL, low density lipoprotein.

*Data presented as percent of patients unless otherwise noted.

### Clinical outcomes of hypertension intervention

The hypertension intervention program was associated with significant improvements in simulated 10-year risk across all clinical outcomes (Table 2). The simulation results indicate that slightly less than doubling the current SBP control rate (ie, from 23.9% to 40.0%) would lead to a 3.8% absolute risk reduction (12.9% relative risk reduction) in MACE while the CVD death rate would improve by 2.7% (absolute risk reduction; 13.2% relative risk reduction). The scenario with 60% SBP control rate would lead to an 8.5% absolute risk reduction (28.9% relative risk reduction) in 10-year MACE rate and a 6.1% absolute risk reduction (29.6% relative risk reduction) in 10-year CVD death rate.

**Table 2:** 10-Year event rates (95% confidence intervals) and risk reduction (95% confidence intervals) in event rate in the Russian hypertensive population by SBP control rate scenario.

	MACE	Cerebro-vascular event diagnosis	MI diagnosis	CHD death	Cerebro-vascular (stroke) death	CVD death	Life expectancy, years
Current care, 23.9% SBP control	0.294 (0.291–0.297)	0.143 (0.140–0.145)	0.048 (0.046–0.049)	0.130 (0.128–0.132)	0.090 (0.088–0.092)	0.208 (0.205–0.210)	15.94
**Absolute risk reduction**							
Absolute difference of current care from:							
40% SBP control rate	−0.038 (−0.042–−0.034)	−0.021 (−0.024–−0.018)	−0.007 (−0.009–−0.005)	−0.019 (−0.022–−0.016)	−0.011 (−0.014–−0.008)	−0.027 (−0.031–−0.024)	+0.93
60% SBP control rate	−0.085 (−0.089–−0.081)	−0.047 (−0.050–−0.044)	−0.015 (−0.017–−0.013)	−0.042 (−0.045–−0.039)	−0.025 (−0.028–−0.022)	−0.061 (−0.065–−0.058)	+2.09
**Relative risk reduction**							
Relative risk reduction with respect to current care from:							
40% SBP control rate	12.9 (11.6–14.2)	14.6 (12.5–16.7)	14.2 (10.3–18.1)	14.5 (12.3–16.7)	12.4 (9.6–15.2)	13.2 (11.5–14.8)	–
60% SBP control rate	28.9 (27.8–30.1)	32.8 (31.0–34.6)	31.8 (28.5–35.1)	32.4 (30.6–34.3)	27.9 (25.4–30.3)	29.6 (28.1–31.0)	–

CHD, coronary heart disease; CVD, cardiovascular disease; MACE, major adverse cardiac event; MI, myocardial infarction; SBP, systolic blood pressure.

**Figure 1:**
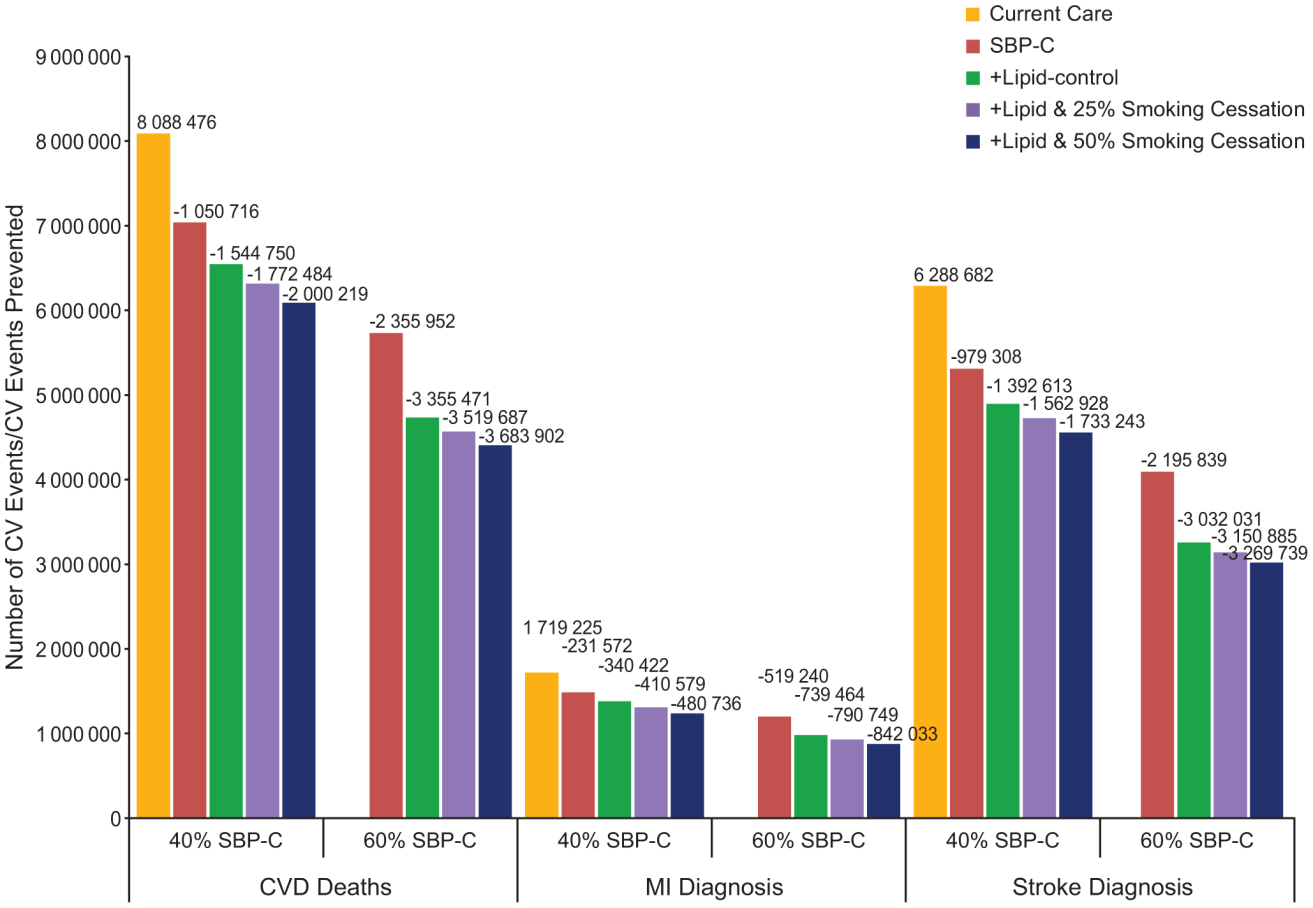
10-Year total number of CV events and CV events prevented in Russian hypertensive population by intervention scenario. CV, cardiovascular; CVD, cardiovascular disease; MI, myocardial infarction; SBP-C, systolic blood pressure control.

The simulated number of events and cases prevented with increased SBP control rates in the 10-year period are presented in Figure 1. Increasing the SBP control rate from current 23.9% to 40.0% would prevent 1.0 million CV deaths, 980 000 stroke diagnoses, and 230 000 MI diagnoses over 10 years in the Russian hypertensive population. A further increase of SBP control to 60% would prevent 2.4 million CV deaths, 2.2 million stroke diagnoses, and 520 000 MI diagnoses.

Life expectancy in the Russian hypertensive population is estimated to increase by 0.93 and 2.09 years at 40% and 60% SBP control, respectively (Table 2). When simulating the impact of the hypertension intervention in the 38% of patients in the working age hypertensive subpopulation, an increase from current care to 40% SBP control led to a gain of 1.21 life-years (CVD death rate reduction of 1.1% [absolute] and 14.9% [relative]; Table 3).

**Table 3:** 10-Year event rates (95% confidence intervals) for CVD death, risk reduction (95% confidence intervals), and life expectancy in the working age Russian hypertensive population by SBP control rate scenario.

	CVD death	Life expectancy, years
Current care, 23.9% SBP control	0.076 (0.073–0.078)	24.05
**Absolute risk reduction**		
Absolute difference of current care from:		
40% SBP control rate	−0.011	+1.21
	(−0.015–−0.008)	
60% SBP control rate	−0.025	+2.72
	(−0.029–−0.022)	
**Relative risk reduction**		
Relative risk reduction with respect to current care from:		
40% SBP control rate	14.9	–
	(10.6–19.1)	
60% SBP control rate	33.4	–
	(29.8–36.9)	

CVD, cardiovascular; SBP, systolic blood pressure.

### Clinical outcomes of dyslipidemia and smoking cessation interventions

**Figure 2:**
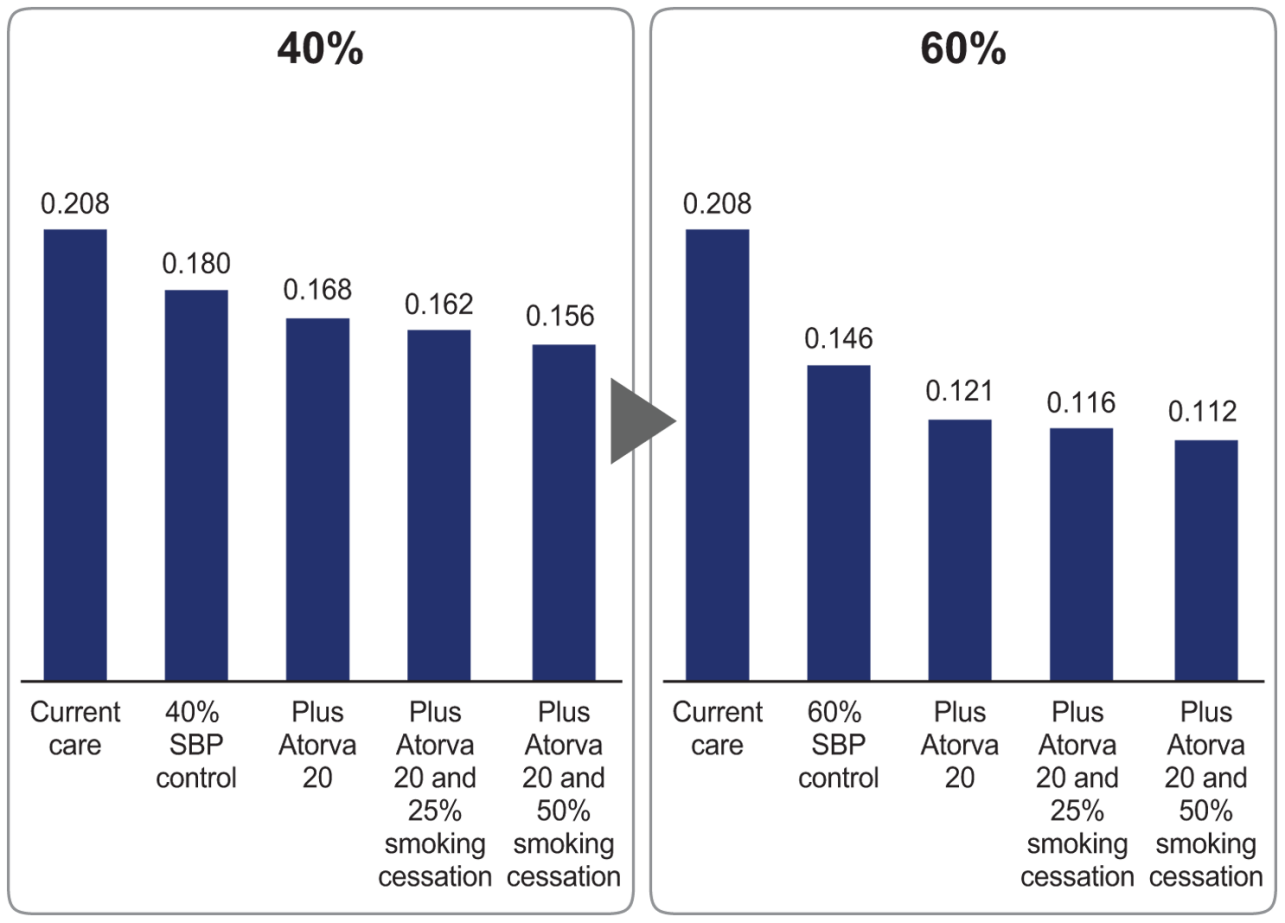
10-Year CVD mortality rates in the Russian hypertensive population by intervention scenario. CVD, cardiovascular disease; SBP, systolic blood pressure.

Simulation results with the addition of the lipid-lowering treatment and smoking cessation program are presented in Figure 2. The addition of atorvastatin 20 mg to the antihypertensive combination pill further reduced the 10-year CVD death rate by 1.2% if a BP control rate of 40% is achieved and by 2.5% if a BP control rate of 60% is achieved. These combined interventions would prevent in total 1.5 million CVD deaths, 1.3 million stroke diagnoses, and 340 000 MI diagnoses with a 40% SBP control rate over 10 years in the Russian hypertensive population (Figure 1). Treatment of dyslipidemia in a hypertensive patient population with 60% SBP control would prevent 3.4 million CVD deaths, 3.0 million MI diagnoses, and 740 000 stroke diagnoses.

With the addition of a smoking cessation program effective in 25% of patients, the 10-year CVD death rate is estimated to improve by 0.6% with a 40% SBP control rate (Figure 2). In addition to the 40% SBP control and lipid-lowering treatment, a smoking cessation program effective in 50% of patients would reduce 10-year CVD death rate by 1.2%, while preventing a total of 2.0 million CVD deaths. A 60% SBP control rate, plus dyslipidemia control and smoking cessation programs at 25% and 50% effectiveness would prevent 3.5 million and 3.7 million CVD deaths, respectively (Figure 1).

### Economic outcomes

**Figure 3:**
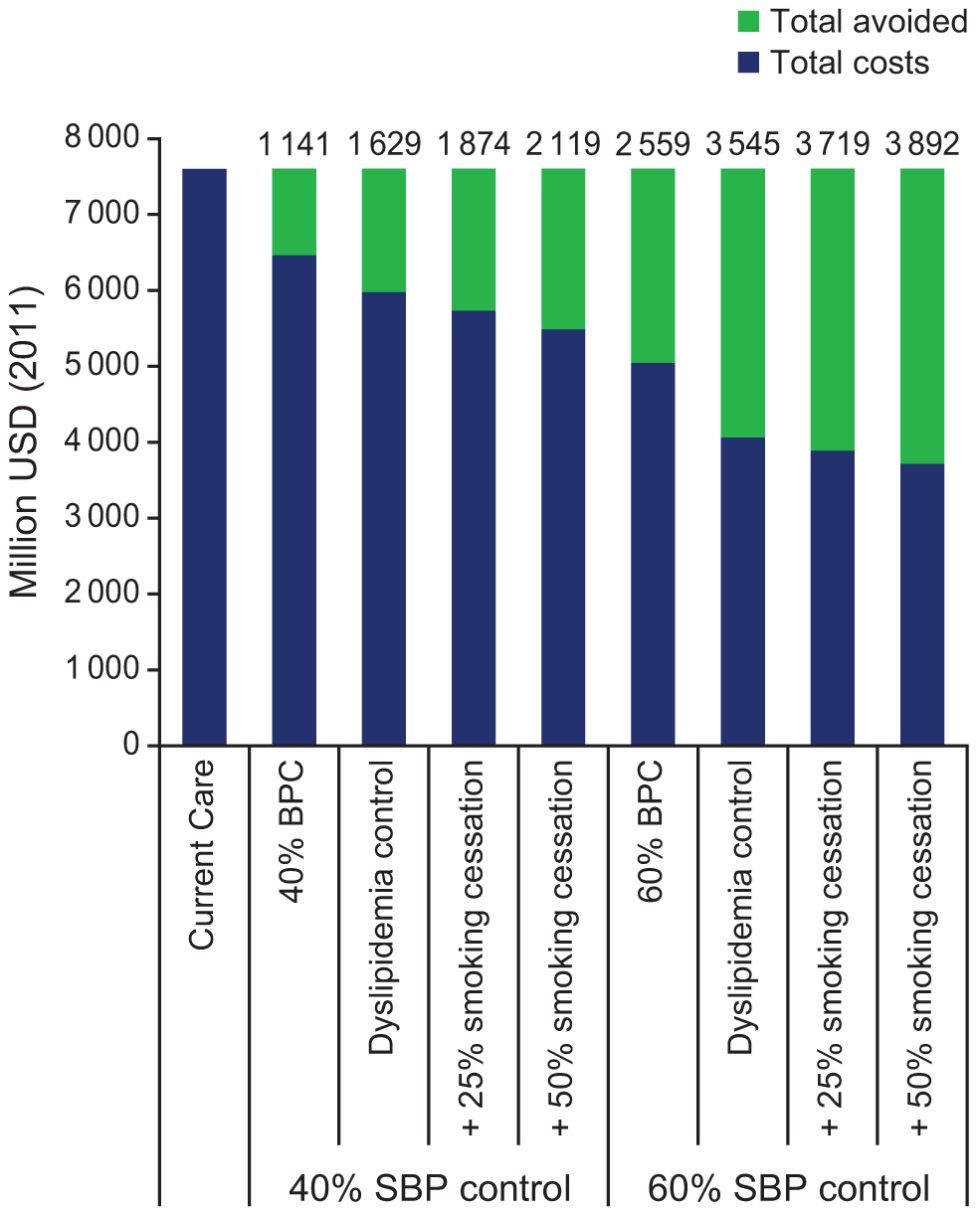
10-Year averted direct health care costs by intervention scenario. BPC, blood pressure control; SBP systolic blood pressure; USD, United States dollars.

At current BP control rates, the cost of MI and cerebrovascular event care in Russia is estimated at US $7.6 billion over 10 years (Figure 3). This represents an annualized cost of MI and cerebrovascular treatment of roughly 0.04% of GDP in 2011 [42]. Increasing SBP control from baseline to 40% would save US $1.1 billion over 10 years, representing 15% of current care costs. Increasing SBP control from baseline to 60% would avert US $2.6 billion over 10 years, representing 34% of current CVD-related care costs. Implementing a smoking cessation intervention effective in 50% of patients, while also adding lipid control and reaching 40% and 60% BP control, would potentially avert care costs equivalent to US $2.1 billion and US$3.9 billion, respectively, over 10 years.

### Sensitivity analysis

The stability of the model simulation was tested by altering various scenarios in a deterministic sensitivity analysis. Analyses included: altering time horizon (20 years), increasing the age-adjusted CV mortality rates used for the model calibration for the general Russian population by 10% (relative change), increasing the prevalence of hypertension by 20% (relative change), and increasing the proportion of diagnosed versus undiagnosed CV events in Russia by 10% (absolute change) from 40% to 50% for MI and from 70% to 80% for cerebrovascular events. As expected, increasing the time horizon from 10 to 20 years showed that improving the SBP control rate from 23.9% to 40% leads to a 4.7% absolute risk reduction in CVD death at 20 years compared with 2.7% absolute risk reduction in 10 years. Absolute risk reductions thus change as expected with changes in the time horizon. The risk reduction results were stable for the other sensitivity analyses across all scenarios (data not shown), demonstrating that the simulation results are insensitive to moderate variations in calibration parameters.

## Discussion

This study aimed to evaluate the potential impact of improved management of known CV risk factors (hypertension, smoking, and dyslipidemia) in Russia over a 10-year time frame. Our analysis shows that improved hypertension control can lead to an increase in life expectancy of 0.93 years, as a result of a 28.9% relative risk reduction in CVD death rates, at an increase from current SBP control rate to 40%. The addition of smoking cessation and dyslipidemia control would further reduce the number of adverse CV events.

Along with the clarion call for expanding Universal Health Coverage globally [43], the World Health Organization (WHO) has also emphasized the urgency of strengthening primary care services worldwide [44]. In addition, the management of hypertension, one of the most common chronic health conditions, has been suggested as a proxy measure of clinical performance [45], [46]. The contribution of hypertension to total mortality rates in Russia is approximately 35% [47]. In addition, men and women with elevated SBP (>180 mmHg) live 12 and 6 years fewer than those with normal BP, respectively [47]. Risk-factor intervention programs targeted to hypertension control—and the important contribution of systolic hypertension control—is therefore of great importance for Russia.

Dyslipidemia and smoking control were assessed as additional interventions to hypertension control alone. The further reductions in CV events highlight the importance of a multifactorial approach to target CV event reduction. Smoking is a particularly important risk factor in Russia as it is estimated that 60% of men and 22% of women in Russia smoke tobacco [28]. Of note, compared with a decline in smoking rates in most industrialized countries, the proportion of smokers in Russia has steadily increased. This is particularly true for women, with the proportion of smokers doubling over 12 years [48]. Although other risk factors such as poor eating habits, high alcohol intake, physical inactivity, and obesity contribute to CV morbidity and mortality in Russia [48], [49], the results give a clear indication of the potential gains to be had in mortality and morbidity by addressing 3 of the most common, treatable risk factors.

The increased BP control rates for the different intervention scenarios in the model simulation were based on an increase in adherence to the antihypertensive combination pill. Although there is substantial evidence on the efficacy of the combination pill [36] from a health care management perspective additional efforts should be made to stimulate adherence and persistence rates with lifestyle modification and medical therapies. Several studies have demonstrated the positive link between a single combination pill and adherence rates [50], [51]. Using a single combination pill compared with the use of 2 separate drugs reduced the risk of nonadherence by 24% [50]. In addition, 2 large-scale, noninterventional studies conducted in a real-world setting in Russia with combination therapies similar to those used in our model have demonstrated clinically significant reductions in BP [52], [53].

The simulation results indicate that improved hypertension management could increase life expectancy by almost 1 year at 40% SBP control and by 1.2 years for the working age subpopulation. As deaths in Russia occur at younger ages compared with high-income countries, premature deaths in the working age population have a disproportionately high impact on the overall economy. In Russia, the number of working years lost in men and women is estimated at 22.1 and 3.4 years, respectively, while the number of working days lost due to CVD is estimated at 474.2 per 100 000 population [6]. With 38% of the hypertensive population being of working age, there is the potential for significant reductions in indirect costs with CV risk-factor management within this subgroup. Indeed, indirect costs due to CV morbidity and mortality compose 80% of total CV related costs, mainly related to lost productivity [6].

Direct health care cost savings related to reduced CVD events were estimated to be US$1.1 billion over 10 years. While these estimations did not include the added costs of the intervention program, cost of hospital pharmacy medications, nor the indirect cost savings, the direct cost offsets demonstrate the potential economic magnitude of improving effective primary care in Russia. Other studies with intervention programs similar to ours have demonstrated the economic benefits of cardiovascular disease prevention. A cardiovascular disease prevention program involving monitoring and counseling of patients with risk factors (among others hypertension) to be highly cost-effective in men aged 40–59 years [54]. Moreover, scaling up a multidrug regimen similar to ours in high-risk individuals (defined as those having had non-fatal CHD) was found to be effective with moderate increases in health expenditure [55].

The averted costs estimated in our calculation should thus be used to fund improved primary care delivery and further CV risk-factor intervention programs to address the number one burden of excess mortality in Russia.

Considering the results of our simulation, the introduction of CV risk-factor preventative programs in Russia can have a substantial health and economic effect. The means of introducing nationwide prevention of CVD in Russia should include policy mechanisms, legal and regulatory frameworks, health care system measures, training, public education, monitoring systems, and international cooperation [48]. Despite CV event reduction recently becoming a goal in Russia, there are several barriers to reducing these risk factors in Russia, including lack of adequate legislation, scarce resources (recently beginning to be addressed with the introduction in 2013 of a large-scale population screening and behavioral intervention program), lack of consistency in health care policy [48], and only partial reimbursement of hospital pharmacy medications. Although our analysis did not estimate the cost of introducing the intervention programs used in the simulation, other studies have shown that similar preventative programs can be introduced at fairly low cost in low- and middle-income settings [55]. In addition, the recent experience in Yaroslavl region saw a significant improvement in BP control rate through a systematic, evidence-based complex change-management program with modest increase in resources primarily focusing on management of the existing health system resources [38].

Our simulation hinges on the importance of appropriate dosing and patients' adherence to effective, evidence-based therapies. This graphically demonstrates the need to better understand barriers to physician inertia and also patient adherence in addressing the burden of non-communicable diseases in Russia. One important implication is to ensure proper funding mechanisms in order to invest in training and management of the extensive Polyclinic system, as well as addressing the heavy burden of out-of-pocket spending that the majority of patients currently have to bear in the out-patient setting [6].

Other countries with relatively high rates of hypertension have been successful in improving BP control. For example, an analysis of the Canadian population showed that, between 1994 and 2004, the rate of death (standardized for age and gender) from acute MI decreased by 38.1% and the standardized rate of death from stroke decreased by 28.2% [56]. Other changes over this period included an increase in BP control rate from 13.2% in 1992 to 64.6% in 2009 [57], and an increase in initial prescriptions of antihypertensive drugs after the introduction of the Canadian Hypertension Education Program in 1999 [58]. Although there could be multiple explanations for the decrease in CV death rates in Canada between 1994 and 2004, these findings demonstrate the potential benefits that increased use of antihypertensive treatment and increased BP control rates could have on CV outcomes in a hypertensive population. In California, USA, implementation of a large-scale hypertension program was associated with a significant increase in hypertension control. Key elements of the program included a comprehensive hypertension registry, development of performance metrics, evidence-based guidelines, and single-pill combination pharmacotherapy. Over the 9-year study period, hypertension control almost doubled from 43.6% to 80.4% [59].

Hypertension treatment practices have also been extensively studied in the Czech Republic [60], [61]. Cardiovascular risk factors were evaluated over the 22–23 years from the WHO MONICA (MONItoring trends and determinants in CArdiovascular disease) survey in 1985 to the most recent one in 2007/2008 [61]. During this time period, rates of hypertension awareness increased (men, 41% to 68%; women, 59% to 71%), as did rates of treatment (men, 21% to 58%; women, 39% to 59%). This resulted in improved levels of hypertension control (men, 3% to 24%; women, 5% to 25%) [61]. These improvements in hypertension control could explain the observed reductions in CV death in the Czech Republic over time (from 561 per 100 000 [1994] to 357 per 100 000 [2009]) [62].

As described earlier, Russia (the Yaroslavl region) has also been successful in improving hypertension control [38]. Following an initial cross-sectional survey of hypertensive patients (n = 1794), a comprehensive health system improvement program for the Yaroslavl region was initiated. One year later, a second patient survey (n = 2992) using very similar methodology across all 38 major Polyclinics in the Region demonstrated significant improvement over the year in BP control rate, from 16.8% to 23.0%, reflecting a 37% relative improvement (p<0.0001) and showing that improvement in BP control is possible across a Russian region.

This study has several strengths. To our knowledge, it is the first study to quantify the potential clinical and economic impact of different CV risk-factor interventions in Russia. An advantage of mathematical models is that they can evaluate such impact without the use of clinical trials or observational studies, which may not be feasible due to limitations in time or resources or ethical constraints. The analytical approach is thus a solid and pragmatic approach to enable informed resource allocation decision making. The Archimedes Model also has the benefit of using differential equations, which maintain the continuous nature of biological variables, time, and their interaction. Another advantage of the Model is that it has been calibrated and validated against a large number of data sets. In addition, the current model has been calibrated to allow it to capture the event rates observed in Russia, with simulated baseline characteristics matching those of the Russian population.

We recognize that there are some limitations to this analysis. First, there is a dearth of high-quality longitudinal data on CVD progression and the impact of CVD risk-factor interventions in the Russian population. There are also clear differences in the baseline characteristics of the population that we used in the Model compared with the Russian population. It is possible that other important risk factors (such as high alcohol consumption) [63] could explain part of the increased CV risk in the Russian population and may reduce the potential benefit of improved BP control through the use of an antihypertensive combination pill. Longitudinal data from a Russian cohort are needed to perform a more extensive analysis to improve the risk calibration of the Model. Also, because MI incidence and cerebrovascular event incidence for Russia were not available for this simulation study, we had to use diagnosed/undiagnosed ratios for MI and for cerebrovascular event incidences observed in the Yaroslavl region and then extrapolate it to the entire country. In addition, because the focus of the study was on the reduction in CV outcomes, the Model is only calibrated to the age-adjusted CVD mortality rate in Russia. However, since the gain in life expectancy from improved SBP control is mostly attributable to the reduction in CVD mortality, non-CV mortality should have little impact on the simulated life expectancy.

## Conclusions

Our simulation implies that a clinically significant number of CV events in the Russian hypertensive population may be prevented by improving BP control. Over a 10-year period, we estimate that over a million deaths could be avoided and that the Russian hypertensive population could, on average, live an additional year of life by doubling the BP control rate. Additional gains in BP control and other more aggressive CV risk-factor management (dyslipidemia and smoking) could result in further improvements that have the potential to positively impact and extend the lives of millions of Russian people, which would be of particular importance to the hypertensive working age population to avert productivity losses. Of importance to policy makers is that our work highlights the potential gains to be had from structured investments in more effective primary care mechanisms and interventions that lead to better hypertension control and other CV risk-factor mitigation. Such a program should be implemented in concert with tracking key performance metrics in order to deliver better outcomes and a healthier, more productive, and active population. In addition, detailed economic calculations of such a program (including costs of its implementation and additional medications) and comparison with economic savings from the reduction of complications would be helpful for future decision making in this area.

## Supporting Information

Table S1 **10-Year event rates (95% confidence intervals) and risk reduction (95% confidence intervals) in event rate in the Russian hypertensive population by SBP control rate scenario.**
(DOCX)

File S1 **Appendix S1: Description of the Archimedes Model. Appendix S2: Validation Methodology and Results. Appendix S3: Age Standardized Mortality in Russian Federation and Yaroslavl Region in 2010. Appendix S4: Incidence of cardiovascular diseases according to Yaroslavl Region's department of health (DoH) statistics in 2010.**
(ZIP)

## References

[1] Russian Federal Service of State Statistics (2011) [Healthcare in Russia]. Available: http://www.gks.ru/bgd/regl/b11_34/isswww.exe/stg/d01/01-86.htm. Accessed June 19, 2014.

[2] Nichols M, Townsend N, Luengo-Fernandez R, Leal J, Gray A, et al (2012) European cardiovascular disease statistics 2012. European Heart Network, Brussels, European Socity of Cardiology, Sophia Antipolis Available: http://www.escardio.org/about/Documents/EU-cardiovascular-disease-statistics-2012.pdf. Accessed June 19, 2014.

[3] Marquez PV (2005) Dying too young: addressing premature mortality and ill health due to non-communicable diseases and injuries in the Russian Federation. World Bank Available: http://siteresources.worldbank.org/INTECA/Resources/Dying_too_Young_Summary_UPDATED_Oct_19.pdf. Accessed June 19, 2014.

[4] Eberstadt N, Shah A (2009) Russia's demographic disaster. American Enterprises Institute for Public Policy Research Available: http://www.aei.org/files/2009/05/19/RO%20Special%20Edition%20Eberstadt-g.pdf. Accessed June 19, 2014.

[5] Shalnova SA, Deev AD (2011) Russian mortality trends in the early XXI century: official statistics data. Cardiovasc Ther Prev 10: 5–10.

[6] Kontsevaya A, Kalinina A, Oganov R (2013) Econcomic burden of cardiovascular diseases in the Russian Federation. Value Health Reg Issues 2: 199–204. 10.1016/j.vhri.2013.06.010 29702865

[7] Oganov RG, Timofeeva TN, Koltunov IE, Konstantinov VV, Balanova YA, et al (2011) Arterial hypertension epidemiology in Russia: the results of 2003–2010 federal monitoring. Cardiovasc Ther Prev 10: 9–13.

[8] The Health and Social Care Information Centre (2012) Health survey for England 2011: health, social care and lifestyles. Summary of key findings Available: http://www.hscic.gov.uk/catalogue/PUB09300/HSE2011-Sum-bklet.pdf. Accessed June 19, 2014.

[9] Yoon SS, Burt V, Louis T, Carroll MD (2012) Hypertension among adults in the United States, 2009–2010. NCHS data brief No. 107. National Center for Health Statistics Available: http://www.cdc.gov/nchs/data/databriefs/db107.pdf. Accessed June 19, 2014.

[10] Godet-Mardirossian H, Girerd X, Vernay M, Chamontin B, Castetbon K, et al (2012) Patterns of hypertension management in France (ENNS 2006–2007). Eur J Prev Cardiol 19: 213–220. 21450611 10.1177/1741826710394303

[11] Wilkins K, Campbell NR, Joffres MR, McAlister FA, Nichol M, et al (2010) Blood pressure in Canadian adults. Health Rep 21: 37–46. 20426225

[12] Belousov Y, Leonova MV, Steinberg LL, Galitsky A, Belousov DY (2009) Monitoring of the medical-social aspects of the efficiency of the treatment of patients with arteiral hypertension in Russia (according to the results of the PIFAGOR III pharmacoepidemiologic study). Problems of Standardization in Healthcare No. 7–8.

[13] Tolpygina SN, Polyanskaya TN, Martsevich SY (2013) Treatment of patients with chronic ischemic heart disease in real clinical practice according to the data from PROGNOZ IBS register (part 1). Rational Pharmacother Card 9: 138–142.

[14] Marcevich SY, Ginzburg ML, Kutishenko NP, et al (2012) The LIS study (Lyubertsy study of mortality in patients with acute myocardial infarction). Evaluation of the pharmacotherapy. Part 1. Treatment of patients before myocardial infarction and its influence on hospital mortality rate. Rational Pharmacother Card 8: 681–684.

[15] Mozheyko M, Eregin S, Vigdorchik A, Hughes D (2012) A cross-sectional survey of hypertension diagnosis and treatment practices among physicians in Yaroslavl Region, Russia. Adv Ther 29: 1016–1025. 23203238 10.1007/s12325-012-0064-2

[16] Kobalova ZD, Starostina EG, Kotovskaia IV, Villeval'de SV, Baranova EI, et al (2008) [Antihypertensive treatment compliance and obstacles to its improvement. Results of Russian program ARGUS-2]. Ter Arkh 80: 76–82. 18441691

[17] Roberts B, Stickley A, Balabanova D, McKee M (2012) Irregular treatment of hypertension in the former Soviet Union. J Epidemiol Community Health 66: 482–488. 21051778 10.1136/jech.2010.111377

[18] Greenberg HM, Galyavich AS, Ziganshina LE, Tinchurina MR, Chamidullin AG, et al (2005) Identification and management of patients with hypertension in the polyclinic system of the Russian Federation. Am J Hypertens 18: 943–948. 16053991 10.1016/j.amjhyper.2005.01.012

[19] Kobalava ZD, Kotovskaia IV, Starostina EG, Villeval'de SV, Luk'ianova EA, et al (2007) [Problems of a physician-patient interaction and control of arterial hypertension in Russia. Main results of scientific-practical program ARGUS-2]. Kardiologiia 47: 38–47. 17495848

[20] Kobalava ZD, Kotovskaya YV, Villevalde SV, Moiseev VS (2009) Russian ARGUS-2 study (2009) Treating hypertension by rational use of diuretics: results of the Russian ARGUS-2 Study. Curr Med Res Opin 25: 2229–2237. 19622008 10.1185/03007990903157531

[21] US Department of Health and Human Services Services (2004) The Seventh Report of the Joint National Committee on Prevention, Detection, Evaluation, and Treatment of High Blood Pressure. NIH Publication No 04-5230.

[22] Schlesinger L, Eddy DM (2002) Archimedes: a new model for simulating health care systems the mathematical formulation. J Biomed Inform 35: 37–50. 12415725 10.1016/s1532-0464(02)00006-0

[23] Schuetz CA, Alperin P, Guda S, van Herick A, Cariou B, et al (2013) A standardized vascular disease health check in Europe: a cost-effectiveness analysis. PLoS One 8: 1–11. 10.1371/journal.pone.0066454 PMC3712021 23869204

[24] Eddy D, Cohen M, Shum K, Dziuba J (2013) Validation methodology and results. ARCHeS Simulator 2.5. Archimedes Available: http://archimedesmodel.com/sites/default/files/Archimedes-Validation-ARCHeS-Simulator-2.5.pdf. Accessed June 19, 2014.

[25] World Health Organization (2012) World Health Statistics 2012. Available: http://apps.who.int/iris/bitstream/10665/44844/1/9789241564441_eng.pdf. Accessed June 19, 2014.

[26] Go AS, Mozaffarian D, Roger VL, Benjamin EJ, Berry JD, et al (2013) Heart disease and stroke statistics–2013 update: a report from the American Heart Association. Circulation 127: e6–245. 23239837 10.1161/CIR.0b013e31828124ad PMC5408511

[27] Posnenkova OM, Kiselev AR, Gridnev VI, Shvartz VA, Dovgalevsky PY, et al (2011) Pharmacotherapy quality in patients with arterial hypertension observed in primary care practice. Hypertension register data. Rational Pharmacother Cardiol 7: 725–732.

[28] World Health Organization (2009) Global Adult Tobacco Survey (GATS). Russian Federation 2009 Country Report Available: http://www.who.int/tobacco/surveillance/en_tfi_gats_russian_countryreport.pdf. Accessed June 19, 2014.

[29] Russian Federation Statistical Service (2010) The Demographic Yearbook of Russia. 2010: Statistical Handbook. Moscow Available: http://www.gks.ru/doc_2010/demo.pdf. Accessed June 19, 2014.

[30] James PA, Oparil S, Carter BL, Cushman WC, Dennison-Himmelfarb C, et al (2014) 2014 Evidence-based guideline for the management of high blood pressure in adults. Report from the panel members appointed to the eighth Joint National Committee (JNC 8). JAMA 311: 507–520. 24352797 10.1001/jama.2013.284427

[31] Mancia G, Fagard R, Narkiewicz K, Redon J, Zanchetti A, et al (2013) 2013 ESH/ESC Guidelines for the management of arterial hypertension. Eur Heart J 34: 2159–2219. 23771844 10.1093/eurheartj/eht151

[32] Calhoun DA, Lacourcière Y, Chiang YT, Glazer RD (2009) Triple antihypertensive therapy with amlodipine, valsartan, and hydrochlorothiazide: a randomized clinical trial. Hypertension 54: 32–39. 19470877 10.1161/HYPERTENSIONAHA.109.131300

[33] Oparil S, Melino M, Lee J, Fernandez V, Heyrman R (2010) Triple therapy with olmesartan medoxomil, amlodipine besylate, and hydrochlorothiazide in adult patients with hypertension: The TRINITY multicenter, randomized, double-blind, 12-week, parallel-group study. Clin Ther 32: 1252–1269. 20678674 10.1016/j.clinthera.2010.07.008

[34] Law MR, Morris JK, Wald NJ (2009) Use of blood pressure lowering drugs in the prevention of cardiovascular disease: meta-analysis of 147 randomised trials in the context of expectations from prospective epidemiological studies. BMJ 338: b1665. 19454737 10.1136/bmj.b1665 PMC2684577

[35] Jones PH, Davidson MH, Stein EA, Bays HE, McKenney JM, et al (2003) Comparison of the efficacy and safety of rosuvastatin versus atorvastatin, simvastatin, and pravastatin across doses (STELLAR* Trial). Am J Cardiol 92: 152–160. 12860216 10.1016/s0002-9149(03)00530-7

[36] Dudl RJ, Wang MC, Wong M, Bellows J (2009) Preventing myocardial infarction and stroke with a simplified bundle of cardioprotective medications. Am J Manag Care 15: e88–e94. 19817511

[37] Huxley RR, Perkovic V (2014) The modifiable burden of worldwide mortality from cardiovascular disease. Lancet Diabetes Endocrinol May 16 Epub ahead of print. 10.1016/S2213-8587(14)70040-3 24842600

[38] Mozheyko M, Eregin S, Vigdorchik A, Tobe S, Campbell N, et al (2013) Changes in hypertension treatment in the Yaroslavl region of Russia: improvements observed between 2 cross-sectional surveys. J Clin Hypertens (Greenwich) 15: 918–924. 24118731 10.1111/jch.12214 PMC4255293

[39] Chazova IE, Ratova LG, Boytsov SA, et al (2010) for the Experts Committe of the Russian Medical Society of Hypertension and Russian Scientific Society of Cardiology (2010) Diagnostics and treatment of arterial hypertension. Russian Guidelines (4th Review): Systemic Hypertension 3: 5–26.

[40] Guallar E, Banegas JR, Blasco-Colmenares E, et al (2011) Excess risk attributable to traditional cardiovascular risk factors in clinical practice setting across Europe – The EURIKA Study. BMC Public Health 11: 704. 21923932 10.1186/1471-2458-11-704 PMC3184074

[41] Pension Fund of the Russian Federation. Pension system principles. Available at: http://www.pfrf.ru/ot_en/system/. Accessed June 19, 2014.

[42] International Monetary Fund (2011) World economic outlook database. Available: http://www.imf.org/external/pubs/ft/weo/2011/02/weodata/index.aspx. Accessed June 19, 2014.

[43] World Health Organization (2013) Universal Health Coverage 2013. Available: http://www.who.int/universal_health_coverage/en/. Accessed June 19, 2014.

[44] World Health Organization (2008) World health report–primary health care: now more than ever. Available: http://www.who.int/whr/2008/08_contents_en.pdf. Accessed June 19, 2014.

[45] Wong MC, Wang HH, Wong SY, Wei X, Yang N, et al (2012) Performance comparison among the major healthcare financing systems in six cities of the Pearl River Delta region, mainland China. PLoS One 7: e46309. 23029474 10.1371/journal.pone.0046309 PMC3460811

[46] Cené CW, Roter D, Carson KA, Miller ER III, Cooper LA (2009) The effect of patient race and blood pressure control on patient-physician communication. J Gen Intern Med 24: 1057–1064. 19575270 10.1007/s11606-009-1051-4 PMC2726885

[47] Oganov RG, Shalnova SA, Maslennikova GY, Deev AD (2001) The role of health lifestyle strategies for public health protection. Ross Med Vesti 3: 34–37.

[48] Petrukhin IS, Lunina EY (2011) Cardiovascular disease risk factors and mortality in Russia. Public Health Rev 33: 436–449.

[49] Zaridze D, Lewington S, Boroda A, Scelo G, Karpov R, et al (2014) Alcohol and mortality in Russia: prospective observational study of 151 000 adults. Lancet 383 9927: 1465–1473 Epub 2014 Jan 31. 24486187 10.1016/S0140-6736(13)62247-3 PMC4007591

[50] Bangalore S, Kamalakkannan G, Parkar S, Messerli FH (2007) Fixed-dose combinations improve medication compliance: a meta-analysis. Am J Med 120: 713–719. 17679131 10.1016/j.amjmed.2006.08.033

[51] Taylor AA, Shoheiber O (2003) Adherence to antihypertensive therapy with fixed-dose amlodipine besylate/benazepril HCl versus comparable component-based therapy. Congest Heart Fail 9: 324–332. 14688505 10.1111/j.1527-5299.2003.03269.x

[52] Chazova IE, Dongre N, Vigdorchik AV (2011) Real-life safety and effectiveness of amlodipine/valsartan combination in the treatment of hypertension. Adv Ther 28: 134–149. 21240661 10.1007/s12325-010-0099-1

[53] Karpov Y, Dongre N, Vigdorchik A, Sastravaha K (2012) Amlodipine/valsartan single-pill combination: a prospective, observational evaluation of the real-life safety and effectiveness in the routine treatment of hypertension. Adv Ther 29: 134–147. 22271158 10.1007/s12325-011-0095-0

[54] Kontsevaya A, Kalinina A, Deev D (2010) Cost-effectiveness of the 5-year cardiovascular program in Russian population. J Hypertens 28: e239 Oral session 6C.

[55] Lim SS, Gaziano TA, Gakidou E, Reddy KS, Farzadfar F, et al (2007) Prevention of cardiovascular disease in high-risk individuals in low-income and middle-income countries: health effects and costs. Lancet 370: 2054–2062. 18063025 10.1016/S0140-6736(07)61699-7

[56] Tu JV, Nardi L, Fang J, Liu J, Khalid L, et al (2009) National trends in rates of death and hospital admissions related to acute myocardial infarction, heart failure and stroke, 1994–2004. CMAJ 180: E118–125. 19546444 10.1503/cmaj.081197 PMC2696549

[57] McAlister FA, Wilkins K, Joffres M, Leenen FH, Fodor G, et al (2011) Changes in the rates of awareness, treatment and control of hypertension in Canada over the past two decades. CMAJ 183: 1007–1013. 21576297 10.1503/cmaj.101767 PMC3114892

[58] Campbell NR, Tu K, Brant R, Duong-Hua M, McAlister FA, et al (2006) The impact of the Canadian Hypertension Education Program on antihypertensive prescribing trends. Hypertension 47: 22–28. 16344380 10.1161/01.HYP.0000196269.98463.fd

[59] Jaffe MG, Lee GA, Young JD, Sidney S, Go AS (2013) Improved blood pressure control associated with a large-scale hypertension program. JAMA 310: 699–705. 23989679 10.1001/jama.2013.108769 PMC4270203

[60] Cifková R, Skodová Z, Lánská V, Adámková V, Novozámská E, et al (2004) Prevalence, awareness, treatment, and control of hypertension in the Czech Republic. Results of two nationwide cross-sectional surveys in 1997/1998 and 2000/2001, Czech Post-MONICA Study. J Hum Hypertens 18: 571–579. 15002000 10.1038/sj.jhh.1001686

[61] Cífková R, Škodová Z, Bruthans J, Adámková V, Jozífová M, et al (2010) Longitudinal trends in major cardiovascular risk factors in the Czech population between 1985 and 2007/8. Czech MONICA and Czech post-MONICA. Atherosclerosis 211: 676–681. 20471016 10.1016/j.atherosclerosis.2010.04.007

[62] Davidkovová H, Kysely J, Kriz B, Vojtisek P, Bobák M (2013) Trends in cardiovascular mortality and hospitalisations, and potential contribution of inhospital case-fatality rates to changes in national mortality in the Czech Republic 1994–2009. Heart 99: 409–416. 23390048 10.1136/heartjnl-2012-303123

[63] Leon DA, Saburova L, Tomkins S, Andreev E, Kiryanov N, et al (2007) Hazardous alcohol drinking and premature mortality in Russia: a population based case-control study. Lancet 369: 2001–2009. 17574092 10.1016/S0140-6736(07)60941-6

