# Supplementary material for: Simulating the Impact of Improved Cardiovascular Risk Interventions on Clinical and Economic Outcomes in Russia
Source: PLoS One. 2014 Aug 20;9(8):e103280. doi: 10.1371/journal.pone.0103280 (PMC4139197; doi:10.1371/journal.pone.0103280)
Supplement: Table S1 — 10-Year event rates (95% confidence intervals) and risk reduction (95% confidence intervals) in event rate in the Russian hypertensive population by SBP control rate scenario. (DOCX) [file pone.0103280.s001.docx]

Table S1

10-Year event rates (95% confidence intervals) and risk reduction (95% confidence intervals) in event rate in the Russian hypertensive population by SBP control rate scenario

|  | **MACE** | **Cerebro-vascular event diagnosis** | **MI  diagnosis** | **CHD  death** | **Cerebro-vascular (stroke) death** | **CVD death** | **Life expectancy, year** |
| --- | --- | --- | --- | --- | --- | --- | --- |
| Current care, 23.9% SBP control | 0.294 | 0.143 | 0.048 | 0.130 | 0.090 | 0.208 | 15.94 |
|  | (0.291 −  0.297) | (0.140 −  0.145) | (0.046 −  0.049) | (0.128 −  0.132) | (0.088 −  0.092) | (0.205 −  0.210) |  |
| **Absolute risk reduction** | | | | | | | |
| Absolute difference of current care from: | | | | | | | |
| 30% SBP control rate | −0.014 | −0.008 | −0.003 | −0.007 | −0.004 | −0.010 | +0.35 |
|  | (-0.019 −  -0.010) | (-0.011 −  -0.005) | (-0.005 −  -0.001) | (-0.010 −  -0.004) | (-0.007 −  -0.002) | (-0.014 −  -0.007) |  |
| 40% SBP control rate | −0.038 | −0.021 | −0.007 | −0.019 | −0.011 | −0.027 | +0.93 |
|  | (-0.042 −  -0.034) | (-0.024 −  -0.018) | (-0.009 −  -0.005) | (-0.022 −  -0.016) | (-0.014 −  -0.008) | (-0.031 −  -0.024) |  |
| 50% SBP control rate | −0.061 | −0.034 | −0.011 | −0.030 | −0.018 | −0.044 | +1.51 |
|  | (-0.066 −  -0.057) | (-0.037 −  -0.031) | (-0.013 −  -0.009) | (-0.034 −  -0.027) | (-0.021 −  -0.015) | (-0.048 −  -0.041) |  |
| 60% SBP control rate | −0.085 | −0.047 | −0.015 | −0.042 | −0.025 | −0.061 | +2.09 |
|  | (-0.089 −  -0.081) | (-0.050 −  -0.044) | (-0.017 −  -0.013) | (-0.045 −  -0.039) | (-0.028 −  -0.022) | (-0.065 −  -0.058) |  |
| **Relative risk reduction** | | | | | | | |
| Relative risk reduction with respect to current care from: | | | | | | | |
| 30% SBP control rate | 4.9 | 5.5 | 5.4 | 5.5 | 4.7 | 5.0 | − |
|  | (3.5 −  6.3) | (3.3 −  7.8) | (1.2 −  9.6) | (3.1 −  7.8) | (1.8 −  7.7) | (3.2 −  6.7) |  |
| 40% SBP control rate | 12.9 | 14.6 | 14.2 | 14.5 | 12.4 | 13.2 | − |
|  | (11.6 −  14.2) | (12.5 −  16.7) | (10.3 −  18.1) | (12.3 −  16.7) | (9.6 −  15.2) | (11.5 −  14.8) |  |
| 50% SBP control rate | 20.9 | 23.7 | 23.0 | 23.5 | 20.1 | 21.4 | − |
|  | (19.7 −  22.1) | (21.7 −  25.7) | (19.4 −  26.6) | (21.4 −  25.5) | (17.5 −  22.7) | (19.8 −  22.9) |  |
| 60% SBP control rate | 28.9 | 32.8 | 31.8 | 32.4 | 27.9 | 29.6 | − |
|  | (27.8 −  30.1) | (31.0 −  34.6) | (28.5 −  35.1) | (30.6 −  34.3) | (25.4 −  30.3) | (28.1 −  31.0) |  |

CHD, coronary heart disease; CVD, cardiovascular disease; MACE, major adverse cardiac event; MI, myocardial infarction; SBP, systolic blood pressure
