# Supplementary material for: Simulating the Impact of Improved Cardiovascular Risk Interventions on Clinical and Economic Outcomes in Russia
Source: PLoS One. 2014 Aug 20;9(8):e103280. doi: 10.1371/journal.pone.0103280 (PMC4139197; doi:10.1371/journal.pone.0103280)
Supplement: File S1 — Appendix S1: Description of the Archimedes Model. Appendix S2: Validation Methodology and Results. Appendix S3: Age Standardized Mortality in Russian Federation and Yaroslavl Region in 2010. Appendix S4: Incidence of cardiovascular diseases according to Yaroslavl Region's department of health (DoH) statistics in 2010. (ZIP) [file pone.0103280.s002.zip › File S1/Appendix S1 Archimedes Model.pdf]

Description of the Archimedes Model

ARCHeS Simulator 2.5

March 2013

David Eddy, MD, PhD
Marc-david Cohen, PhD

ARCHIMEDES
Quantifying Healthcare

Table of Contents

Table of Contents 2

Introduction 5

What's New in ARCHeS with Simulator 2.5 6

Applications 7

Overview of the Model 7

- Physiology Model 8
- Population Model 11
- Healthcare System Model 15
- Outcomes Model 18
- Executing the Model 19
- Validating the Model 20

Physiology Model Variables 20

- Identification and Selection of Data Sources 20
- Disease Diagrams 21

Diabetes Metabolism Model 21

- Type 2 Diabetes Progression Function 23
- Fasting Plasma Glucose, Insulin Efficiency, and HbA1c 24
- Effects on Other Disease Models 25

Diabetic Retinopathy Model 26

- Risk Factors for Non-Proliferative Diabetic Retinopathy 27
- Risk Factors for Proliferative Diabetic Retinopathy 28
- Risk Factors for Diabetic Macular Edema 28
- Blindness 29

Diabetic Neuropathy Model 29

ARCHIMEDES
© 2011-2013

Risk Factors for Sensory Neuropathy 30
Risk Factors for Foot Ulcer 30
Risk Factors for Amputation 31
Nephropathy Model 31
Coronary Artery Disease Model 34
Risk Factors for First Myocardial Infarction 35
Risk Factors for Unstable Angina 36
Risk Factors for Stable Angina 36
Heart Failure Model 37
Chronic Atrial Fibrillation Model 39
Lipids Model 40
Stroke Model 41
First Stroke 42
Recurrent Stroke 45
Chronic Obstructive Pulmonary Disease (COPD) Model 45
Mortality 47
Interventions 48
Aspirin Model 49
Statin Model 50
Diabetes Medications 51
Blood Pressure Medications Model 58
Diabetes Diet Model 60
Healthcare System Model 61
Guidelines Implemented in the Model 61
Datasets Used for Model Calibration 65

© 2011-2013

Limitations of the Model....67

Acknowledgements....69

Sources of Funding....70

© 2011-2013

Introduction

The Archimedes Model is a simulation model of human physiology, patient populations, and healthcare systems. It creates a virtual world with simulated people, each of whom has a simulated anatomy and physiology. They get diseases, develop symptoms, seek care by scheduling appointments with their doctors or visiting emergency rooms, are seen by simulated physicians or other healthcare providers, are prescribed tests and treatments, choose whether or not to comply, and respond to the treatments. These events occur and recur continuously, as they do in real life, until the simulation ends or the patient dies.

This report is a non-technical description of the parts of the Model used in ARCHeS.

ARCHeS

ARCHeS is a suite of healthcare simulation and analytics tools designed to provide answers to questions about the health outcomes and economic effects of different interventions in specific populations and healthcare settings.

Part of ARCHeS provides a web-based interface to the Archimedes Model. It enables users to set up simulations online using the ARCHeS Trial Designer, run them through the Model (also called the "Simulator"), and analyze the results using ARCHeS Outcomes Analyzer. ARCHeS[1] addresses risk factors, interventions, and outcomes related to a variety of conditions, including diabetes and its complications, coronary artery disease, stroke, dyslipidemia, hypertension, nephropathy, obesity, COPD, and smoking. It can be used to answer a wide range of questions about the occurrence and management of those conditions.

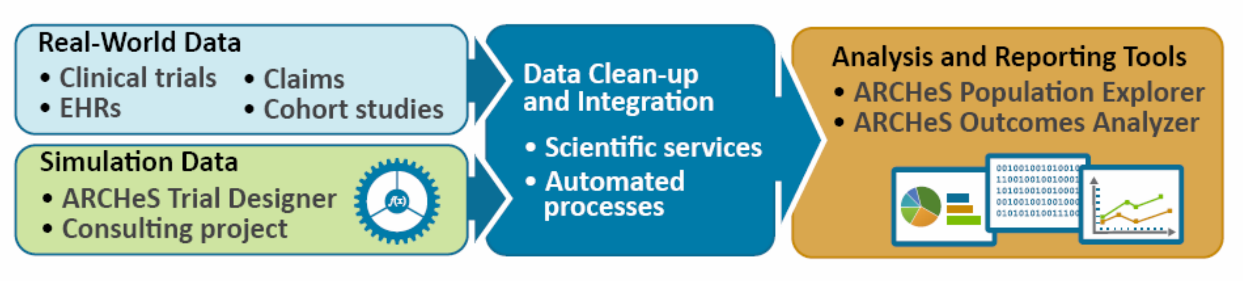

Figure 1. ARCHeS.

Simulator 2.5

The Archimedes Model was designed for multiple applications and repeated use. To date, more than 100 analyses have been conducted using the Model. To enable application of the Model to multiple problems, many of them being conducted simultaneously, we maintain a "base version" of the Model,

[1] Future versions of ARCHeS will be periodically released as the Archimedes Model is improved to incorporate new science and evidence, and as the functionality of ARCHeS is increased.

ARCHIMEDES
© 2011-2013

or “Base Model,” that includes all the variables and equations needed to calculate a person’s physiology, the occurrence and outcomes of diseases, and the effects of tests and interventions. Many questions can be answered with the Base Model. When there is a need to conduct an analysis that cannot be done with the Base Model – e.g., it requires a higher level of physiological or pharmacological detail, involves emerging risk factors or novel interventions, or involves different settings and care processes – then appropriate parts of the Base Model are expanded or modified to address the new question. The modified, project-specific versions are saved for those particular analyses, but the Base Model is maintained unaltered. Over time, new science, technology, and evidence, and/or improvements in software may warrant changes to the Base Model. At that point, a new version of the Base Model is released. ARCHeS uses version 2.5 of the Base Model, called “Simulator 2.5.” This is the version of the Model that is described in this report.

Non-technical description

This report is a non-technical description of the parts of Simulator 2.5 that are accessed by ARCHeS. For convenience, in this report we will use the terms “ARCHeS Simulator 2.5,” and more simply “the Model,” to refer to these parts of the Archimedes Model, with the understanding that the full Archimedes Model includes other diseases that are not in ARCHeS, as well as other versions built using the Archimedes Modeling Framework. This report describes the types of applications ARCHeS is designed to address, the main parts of the Model, the variables and relationships in each of the disease sub-models, the sources used to build the disease sub-models, the sources used to build and calibrate the care processes for preventing or managing the diseases, and limitations of the Model. Thus this report describes *what* the Model does. Validation of Simulator 2.5 is described in the report “Validation Methodology and Results: ARCHeS Simulator 2.5.” All reports can be found on our website at archimedesmodel.com/resource-center.

This report is intended for all audiences. Parties interested in a more technical description of the Model or ARCHeS can arrange for a technical, quantitative description by contacting us at archimedesmodel.com/contact. This report is not intended to be a manual or tutorial on ARCHeS. Additional information about ARCHeS and its functionality can be found on our website.

What’s New in ARCHeS with Simulator 2.5

The following Model improvements are included in Simulator version 2.5:

- A new model of chronic obstructive pulmonary disease (COPD) has been added. Refer to the “Chronic Obstructive Pulmonary Disease (COPD) Model” section of this document for more information.
- The nephropathy model was updated to incorporate new evidence on chronic kidney disease. The new model was built using results from the Chronic Kidney Disease Prognosis Consortium and data from NHANES 1999-2008 and the United States Renal Data System. Refer to the “Nephropathy Model” section of this document for more information.

© 2011-2013

Applications

The Archimedes Model is designed to address questions at a variety of levels, including physiology, pharmacology, management of individual patients, and management of populations. For some case studies and examples of how the Archimedes Model has been applied, see archimedesmodel.com/case-studies. ARCHeS is designed to address questions relating to the management of cardio-metabolic risk in populations. Applications of ARCHeS include: forecasting, cost- and cost-effectiveness analysis, comparative-effectiveness analysis, priority setting, clinical trial design and prediction, performance improvement and incentives, guideline design, drug portfolio selection, the translation of efficacy (for example, clinical trial results) into effectiveness (results that can be expected in real settings), and the design of population-level policies.

Overview of the Model

The Archimedes Model is composed of four main components that work closely together: the physiology model, the population model, the healthcare system model, and the outcomes model (Figure 2). This section provides an overview of each component, focusing in particular on the variables and their relationships. Methods for validating the Model and the results of a standardized suite of validations are available in the report "Validation Methodology and Results: ARCHeS Simulator 2.5," available at archimedesmodel.com/resource-center.

© 2011-2013

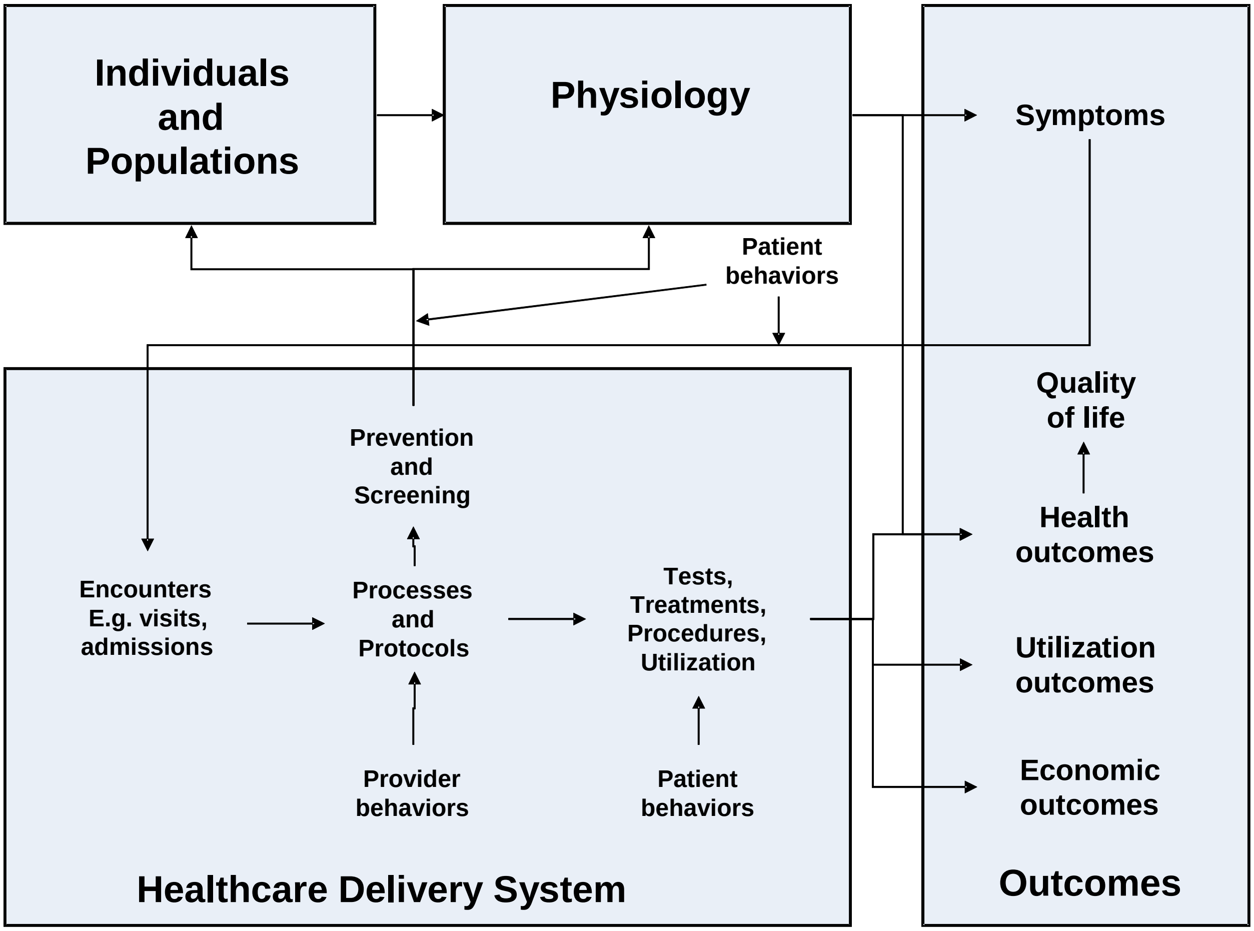

Figure 2. Components of the Archimedes Model.

Physiology Model

The physiology component is the foundation of the Model. It represents the physiology of each simulated person and causes them to get diseases, have symptoms, seek care, and so forth. It includes human anatomy, physiology, disease processes, the effects of interventions (e.g., preventive activities, tests, treatments, procedures), and outcomes.

Physiological variables

The fundamental building blocks are the physiological variables pertinent to clinical decisions and clinical policies. These other variables are considered by physicians and other providers when making decisions about individual patients, considered by those designing and interpreting clinical trials, and considered by those designing and applying policies such as guidelines, performance measures, and incentives. Thus the physiology model includes objects and variables that represent organs and their major parts, physiological variables and biomarkers (e.g. plasma glucose, various cholesterols, cardiac output), demographic variables, health-related behaviors, physical examination findings, past medical history, symptoms, interventions, and health outcomes. The particular variables in each disease sub-model are described below.

© 2011-2013

Level of detail

The particular variables included in the physiology model are determined by the questions the Model is intended to address. For example, because we want the Model to be able to analyze various weight-loss programs, the physiology model was built to include the variable "weight," each simulated person has a weight; weight can be measured in various ways (e.g., weight, body mass index (BMI)); a person's weight affects the progression of other variables (e.g. lipids, fasting plasma glucose (FPG); blood pressure), and a person's weight can be modified by interventions. The Model does not include phenomena at the cellular or sub-cellular level, although the Archimedes Modeling Framework enables extension of the Model to deeper levels to address specific questions.

Trajectories of physiological variables

While some variables are fixed (e.g. sex, race/ethnicity), most variables are continuously valued, continuously changing, and continuously interacting. For example, as in reality each simulated person in the Model at any time has a systolic blood pressure (SBP) which is continuously increasing or decreasing depending on various factors and treatments. The SBP in turn affects the development of atherosclerotic plaque, which can cause an MI. An MI can affect cardiac output, and so forth. Thus the value of any particular variable is a function of time, as well as of the values of other variables. We use the term "trajectory" to describe how the value of a variable changes over time and as a function of other variables.

Tests and symptoms

The physiological variables determine test results, symptoms, and health outcomes. When a test is done (e.g., a FPG test), it reads the value of the pertinent variable and reports it back, possibly modified by any random or systematic biases in the test or its interpretation. In this context, the concept of a test is very general and includes not only laboratory tests but any other activity that collects information about a patient, such as a provider taking a history or doing a physical exam, or a patient filling out a survey. When the values of variables reach certain levels they can cause symptoms and health outcomes. For example, when the value of the variable that represents the occlusion of a coronary artery approaches 75%, the person might experience angina.

Diseases

Diseases are defined in terms of the underlying physiological variables. For example, a person is said to have "diabetes" when their FPG > 125 mg/dL. This reflects the fact that in reality diseases are not physiological "states" that people are "in," but are actually labels that are applied when physiological variables meet certain criteria. By defining diseases in terms of the underlying variables the Model is able to address several issues: concepts of abnormality change; many diseases are "man made" based solely on the results of tests (e.g. "mild hypertension," "pre-diabetes"); many diseases have multiple – often competing – definitions (e.g. "metabolic syndrome"); definitions can change over time (e.g. prior to 1998 the diagnosis of diabetes was based on FPG > 140, not FPG > 125); definitions can be applied differently in different settings' and "diseases" can overlap (e.g., "coronary artery disease," "diabetes,"

© 2011-2013

“cardio-metabolic syndrome”). This approach also enables the Model to accommodate evidence collected at times when different definitions were used.

Treatments

Treatments modify the risk of an event, either through direct modification of the hazard rate (e.g. revascularization), or through changes in biomarkers which affect the hazard rate (e.g. lowering systolic blood pressure with antihypertensive medications), or both. If a treatment is known to affect a symptom or health outcome, but the mechanism of action of that effect is not known, the effect can be specified directly. For example, the risks or side effects of a treatment can be modeled either by specifying the treatment’s effect on another feature if that is known, or by specifying an effect directly on the outcome.

Disease progression variables and functions

In addition to variables that correspond to real physiological phenomena, the Model includes variables that represent physiological constructs for which there are no directly measurable counterparts in reality. An example is the variable that represents insulin resistance. Clinicians and researchers talk about “insulin resistance” as a “cause” of diabetes, even though “resistance” is an abstract construct that cannot be directly observed or measured. In the same way, the Model includes a closely related variable called “insulin efficiency” (the inverse of insulin resistance), which in the Model is the main cause of type 2 diabetes in the simulated people. Another example is “plaque,” the main variable that causes MIs. We call these “disease progression variables,” and the equations that determine their progression over time are called “disease progression functions” [2]. Every disease includes at least one disease progression function, usually named to represent the clinical condition of which it is the cause (e.g. “retinopathy progression variable”).

Like other variables in the Model, disease progression functions are defined at a clinical level of detail. The “plaque progression variable” is a good example; the Base Model does not try to include the development of fatty deposits, introduction of foam cells, or the clotting cascade. The Model can be extended to include these if needed for particular projects (provided there are sufficient data), but they are not included in the Base Model or ARCHeS Simulator 2.5. Because the Model is written at a clinical level of detail, the equations for the disease progression functions frequently take the form of proportional hazard models.

Equations

The Physiological model contains algebraic and differential equations that describe how each variable relates to other variables and how all the variables together change over time (as the patient ages). Thus the equations are not static like a typical risk calculator which takes a person’s current values of a small number of variables and calculates a ten-year risk of some event such as a heart attack. Rather, in the

---

[2] In previous publications we used the term “features” as a synonym for “variables.” For example, see Eddy DM, Schlessinger L. Archimedes: a Trial Validated Model of Diabetes, Diabetes Care 2003, 26:3093-3101.

© 2011-2013

equations in the physiology model, virtually all of the variables are continuous functions of time and other variables. This is important for several reasons. Most importantly, it enables the Model to calculate the effects of changes in risk factors over time. This enables the Model to capture past medical history and past values of risk factors and other variables that affect the occurrence and progression of diseases. It also enables the Model to capture the effects of interventions and other events that may occur in the future.

The equations are based on observed data as recorded in physiological research, surveys, epidemiological studies, registries, and clinical trials. The variables and their relationships for each disease, as well as the sources used to derive the equations for the trajectories and relationships, are described in the section on disease models below.

Data sources

The equations are estimated from a variety of sources, which are listed in the sections that describe the variables and their relationships for each disease. The equations for progression functions are estimated from data on age-specific incidence rates of the clinical events the progression functions are causing. For example, the equation for the type 2 diabetes progression function (corresponding clinically to the "cause" of insulin resistance) can be estimated from data on age-specific incidence rates of type 2 diabetes. This can be done for different populations defined by race/ethnicity or other variables, when there are incidence-rate data for those populations.

Individual variability

Many of the physiology equations include random variables to replicate individual variations in such things as the occurrence and progression of diseases, and the development of symptoms. Random variables are also used in the creation of simulated populations, in care processes, and in patient and physician behaviors, as described below.

Single integrated model

Because we want the Model to be able to compare a variety of interventions that affect different diseases (e.g. for comparative-effectiveness research or priority setting), the Model includes all the variables, outcomes, and interventions in a single integrated model. Use of a single integrated model also enables the Model to address co-morbidities, syndromes that affect multiple organ systems, use of multiple drugs, and drugs that have multiple effects.

User control

Users of ARCHeS cannot modify the physiology model through the ARCHeS interface. If a modification or extension of the Model is desired, contact us at archimedesmodel.com/contact.

Population Model

The second main component of the Model is the population model, which creates simulated people according to specifications provided by the users of ARCHeS.

© 2011-2013

Project population

Two types of populations can be specified. The first is the "project population" in which the analysis will be done and for which results can be reported. For example, if the purpose of a project is to evaluate several different interventions to reduce cardiovascular risk in adults between age 40 and 85 in the United States, then the project population would be defined as "40 < age < 85." The project population can be defined by a wide variety of variables (e.g., demographics, biomarkers, behaviors, past medical history, and current medications). The ARCHeS interface includes tools that enable creation of simulated populations whose baseline characteristics match those of any population for which a user of ARCHeS has baseline data, such as populations defined by geographic regions, employers, health plan membership, and insurance coverage.

Target population

The second type of population is the "target population" for a particular treatment, with the requirement that the target population is within (a subset of) the project population. There can be as many target populations as there are treatments. For example, if one intervention is to give antihypertensive medications to everyone with SPB > 140 mg/dL, then the target population for that intervention is defined by "SBP > 140." Because the target population is always a subset of the project population, the target population in this example is "((40 < age < 85) and (SBP > 140))." This ability to define both project and target populations is particularly important for projects that include or compare multiple interventions that have different target populations. It also facilitates the identification of populations that are most appropriate for particular treatments.

Creation of a simulated population

In the base version of Archimedes, simulated populations are created using person-specific data from the Continuous NHANES survey of the US population. The methods are best described by walking through the process for creating a project population. The process begins by the user specifying inclusion and exclusion criteria for the project population, as is done for clinical trials and other studies. The specifications can include multiple variables, combinations of criteria, and nested criteria. (The ARCHeS interface includes a tool for using pull-down lists of variables and logical tests, and parentheses for specifying nested criteria.) The population model then searches the NHANES database to identify people who meet those criteria.

For each of those real persons, the Model then creates a simulated person who, when he or she is aged from his or her birth (age = 0) to the age of the real person at the time of the survey, will match the real person with respect to all the variables pertinent to cardio-metabolic risk. This is done by calculating parameters for the equations that define the trajectories of the variables; specifically, setting the parameters so that the trajectories of all of the variables in the simulated person match the values of the variables observed in the real person[3]. The result of this process is that for every real person, there

---

[3] The concept can be illustrated with a simple non-medical example. Suppose the variable we are interested in is the distance a person has travelled from some start point. Suppose that after half an hour (i.e., the "age" of the

© 2011-2013

is a simulated person whose variables very closely match the variables of the real person. The match is not exact because of random factors in the physiology model that affect the values of the simulated person's variables while the person is "aging" (i.e., while the person's physiology is being calculated starting from age = 0).

This method of aging the simulated person from birth is important because it captures a person's past medical history and the effects on variables that are important determinants of future events but that cannot be measured and therefore do not appear in surveys or other data sources. An example is the plaque/MI progression function (representing the plaque in the person's coronary arteries), which is not measured directly but is crucial for calculating the probability that an individual will have a coronary artery event in the future.

The results of this part of the process are illustrated in Table 1, which shows the characteristics of the simulated population and compares them to the characteristics of the real NHANES population, in this case for the age group 20 – 85.

Table 1 shows the marginal values of the demographic and biomarker variables. The method also recreates all the correlations between variables.

Table 1. Comparison of Characteristics of Simulated Population and NHANES Population Age 20 – 85.

Variable	Simulation	NHANES
Male fraction	0.48	0.48
Black fraction	0.11	0.11
Hispanic fraction	0.05	0.05
Mexican fraction	0.08	0.08
White fraction	0.71	0.72
Age	46.35	45.99
BMI	28.13	28.32
Cholesterol	198.04	200.63
DBP	71.49	71.83
FPG	100.91	103.86
HDL	53.99	52.56
Hemoglobin	5.42	5.48
LDL	116.59	118.74
SBP	121.43	122.52
Serum creatinine	0.91	0.89

person after the start of the travel = 30 minutes) the person has traveled 30 miles. The equation for the trajectory of distance as a function of time is "Distance = Rate x Time." Given the available information, we can calculate the value of "rate" for this real person; it is 60 mph. We can then create a simulated person and assign them a rate of 60 mph, and know that after a half an hour the simulated person will also have traveled 30 miles. By calculating the parameter "rate" based on information about the real person, and assigning that rate to a simulated person, we have created a simulated person whose distance matches that of the real person. In the Model, this is done for all variables for all people.

© 2011-2013

Fraction who smoke	0.24	0.25
Triglycerides	138.29	144.46
Urinary albumin-creatinine ratio	19.36	33.75

After the simulated population has been created, the population model then displays the population-level data (i.e., the first two columns of Table 1) in the ARCHeS interface. The user of ARCHeS can then review the information, and either accept the values or modify any or all of them. If the user is conducting an analysis for the US population, he or she would not need to modify the population created from the NHANES dataset. However, the user might be interested in a particular geographical area where smoking rates are higher than the US average, or the user might have baseline characteristics from a real trial that they want to replicate. If the user chooses to change any of the population level values, the population model will resample the simulated people to find a subset that has the desired levels of the variables, still subject to the inclusion and exclusion criteria the user originally specified. The population model contains automated methods that select virtual people in a way that causes the selected population to converge on any specified targets for biomarkers and other variables, retaining the correlations between variables, as closely as possible given the size of the NHANES database.

The target population is defined in a similar way, except that users can only specify the inclusion and exclusion criteria. The population model will then apply those criteria to the project population[4]. An example is the ALLHAT hypertension trial, which had the following inclusion and exclusion criteria.

Inclusion criteria

- Age 55 years or older
- Known hypertensive with BP ≤ 160/100 mmHg on treatment, or BP ≥ 140/90 mmHg and ≤ 180/110 without treatment
- At least one of the following:
    - Left ventricular hypertrophy on electrocardiogram or echocardiogram
    - Known atherosclerotic cardiovascular disease (CVD)
    - Type 2 diabetes mellitus
    - HDL cholesterol <35 mg/dl
    - Current cigarette smoker

Exclusion criteria

- Recent MI or stroke
- Known congestive heart failure or angina pectoris

[4] The user cannot modify the characteristics of the target populations because doing so would alter the characteristics of the project population which had previously been set.

© 2011-2013

- Need for any study drug for reasons other than hypertension
- Need for more than two antihypertensive drugs to control BP
- Serious systemic disease
- Elevated serum creatinine (2 mg/dl or greater)

If those criteria are applied to the US population they create a simulated population which can be compared to the ALLHAT population. Table 2 shows the results.

Table 2. Comparison of Baseline Characteristics in ALLHAT and Simulated Populations.

Characteristic	Simulation mean	ALLHAT mean	Simulation std. dev.	ALLHAT std. dev.
Age	66.9	66.9	7.6	7.7
BMI	29.9	29.7	6.2	6.2
DBP	88.8	89.0	10.1	10.0
FPG	125.2	123.5	57.0	58.3
HDL	48.5	46.8	14.5	14.7
SBP	156.0	156.0	13.3	16.0
Total cholesterol	212.1	216.2	43.4	43.0
Fraction with type 2 diabetes	0.37	0.361		
Fraction with history of MI or stroke	0.24	0.232		
Fraction with HDL cholesterol less than 35	0.14	0.116		
Fraction who are male	0.54	0.531		
Fraction who smoke	0.22	0.219		

User control

As the above discussion makes clear, unlike the Archimedes physiology model, which users are not able to modify, users have greater control over the project population and target populations used in an analysis. Again, the match is never exact because of random factors. This is analogous to the random factors that cause the baseline characteristics of treatment and control groups in clinical trials to be slightly different.

Healthcare System Model

Care processes

Health and economic outcomes are determined not only by the characteristics of the population (as determined by the population model) and the physiology of the diseases of interest (as determined by the physiology model) but also by the level of care people are receiving. The Model is designed to represent care as it is delivered in the US today, on average. Thus the Model includes care processes that represent current national guidelines. Examples are the Adult Treatment Panel (ATP) III guideline

© 2011-2013

for cholesterol and the American Diabetes Association (ADA) guideline for managing HbA1c in people with diabetes. A list of guidelines incorporated in the base version of the Model is given in the section on the healthcare system model below.

The guidelines are incorporated in the Model by laying them out in the form of pathways (if they are not already in that form), with logical tests at branch points (e.g., "If SBP > 140 mg/dL, then…"). An example is Figure 3, which shows a pathway for diagnosing a patient with hypertension. The pathways are then programmed and inserted into the healthcare system model.

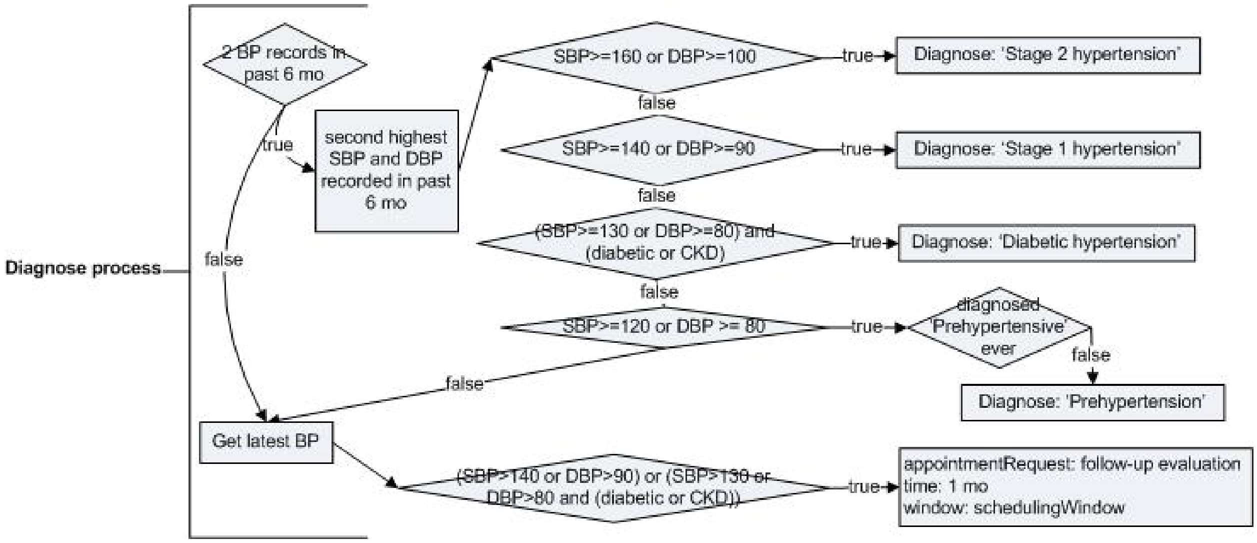

Figure 3. Pathway for Diagnosing a Person with Hypertension.

Ambiguities in a guideline, such as "Test A should be considered" or "Available drugs include…," are examined by in-house physicians and external advisors who have knowledge of the pertinent subject area, supplemented by utilization data if available, to determine how the ambiguities should be addressed in the pathway. If appropriate, probabilities or random variables are inserted at branch points to reflect uncertainties about ambiguous parts of a guideline or variations in practices.

Provider behaviors

Guidelines represent "ideal care" – what the designers of guidelines believe is best supported by the evidence and recommend be followed by all practitioners and patients. However, not all providers follow guidelines, and not all patients adhere to recommended tests and treatments. This is addressed in the Model by assigning probabilities to appropriate branch points, even if the guideline itself is unambiguous, to more realistically represent behaviors relating to performance and adherence. These probabilities can then be calibrated to try to match observed levels performance, compliance, biomarker control, and utilization. Large national datasets, such as the National Ambulatory Care Survey, are used

ARCHIMEDES
© 2011-2013

for this purpose. Other sources for calibrating the care processes are listed in the section on the healthcare system model below.

Calibration of care processes

This is done to the greatest extent permitted by the data. However, the different data sources used to calibrate care processes and behaviors often involve different populations, data collection methods (e.g., surveys, claims data, death certificates, hospital admissions), and definitions of events (e.g., different codes for "coronary heart disease" events). The result is that there can be uncertainty about which source is "correct" for a particular variable or application. This is illustrated in Table 3, which shows six different estimates of the prevalence of diabetes in the US.

Table 3. Six Estimates of Prevalence of Type 2 Diabetes in the US.

NHANES	7.61%
NAMCS and NHAMCS-OPD combined (2006)	10.85%
National Diabetes Fact Sheet (2007)	8.10%
AHA Heart and Stroke Update (2009)	7.70%
BRFSS Prevalence and Trends Data (CDC)	8.30%
Heart and Stroke Update (2005)	7.30%

Furthermore, for some variables such as utilization rates of procedures, the observed rates do not match what is called for by a guideline, under any set of assumptions about performance and adherence. Finally, it is well known that care practices vary widely in different settings. There is no such thing as a single "customary care" or "standard of care" that is applied uniformly throughout the country.

Because of these and other factors, it is not possible to achieve perfect matches for all the variables related to the healthcare delivery system. When there are multiple sources for the same variable, judgments have to be made about which source to use. When it is mathematically impossible to reconcile the sources with each other, or reconcile the observed data with current guidelines, judgments have to be made about which variables are the most important to match. The Model should be thought of as representing "usual" or "customary" care in the same abstract sense as those terms are used in common parlance; it is the "best fit possible" between national guidelines and actual care as reflected in the available data.

Uses of specifying care processes

Despite the limitations of the available data and unavoidable or irreconcilable inconsistencies between national guidelines and actual practices, the explicit incorporation of care processes and behaviors does enable the Model to represent practice patterns more accurately than would otherwise be possible. In particular, it avoids the need to assume that the background care given to patients in a simulation is the same as the background care that was given to people in whatever study that was used to estimate parameters for the Model. (We use the term "background care" to describe all aspects of care other

© 2011-2013

than the interventions being studied in a project.) For example, imagine a state transition model in which the incidence rate of MI (the transition probability from the state "No MI" to the state "MI") is calculated using the Framingham equation used in the ATP III guideline[5]. This rate reflects the behaviors, screening practices, use of preventive interventions, definitions of co-morbid conditions (e.g. the definition of "diabetes" has changed), and definitions of outcomes in use during the period the Framingham data were collected. Use of that equation to analyze a problem today requires an assumption that care today is the same as the care delivered between 1971 and 1986 in Framingham MA.

Based on the calibrated care protocols just described, the healthcare system model simulates the most important aspects of the delivery system, including visits, admissions, laboratory tests, diagnostic and therapeutic procedures, and treatments.

User control

With ARCHeS, users are able to define care processes for delivering interventions relating to cardio-metabolic risk. Currently it is not possible for users to modify or recalibrate guidelines and care processes that represent background care.

Outcomes Model

For each person, the outcomes model tracks every event that could affect any of the four main types of outcomes calculated by the Model: utilization, costs, health outcomes, and quality of life. These events are tracked for each person and the times they occur are recorded. Utilization events include visits, admissions, tests, procedures, and treatments. Costs are calculated by multiplying every cost-generating event (e.g., a visit or lab test) by the cost of that event. Quality of life is calculated by recording the time a patient spends with a particular symptom or health outcome, and multiplying by the factor that represents the decrement in quality of life associated with that symptom or health outcome. Outcomes that occur in any given year can be discounted, and present values calculated.

User control

In the base version of the Archimedes Model, costs other than the costs of interventions such as drugs and smoking cessation programs are based primarily on Medicare. ARCHeS enables users to modify the costs assigned to particular interventions such as drugs and smoking cessation programs. It does not enable users to modify the cost of other cost-generating events in the Model, such as the cost of an emergency room visit for chest pain or the cost of caring for a patient with end-stage renal disease. The ability to change the costs of particular aspects of background care will be available in a future release of ARCHeS. However, ARCHeS does enable users to set a parameter that will modify the overall cost of background care. For example, if a health plan knows that on average its costs are 10% higher than those specified by Medicare, it can set this parameter to 1.1 and the costs of all aspects of background

---

[5] Wilson PWF, D'Agostino RB, Levy D, Belanger AM, Silbershatz H, Kannel WB. Prediction of coronary heart disease using risk factor categories, Circulation 1998; 97:1837-47.

© 2011-2013

care will be increased by 10%. ARCHeS also enables users to modify discount rates for health and/or economic outcomes. Finally, ARCHeS enables users to modify the quality-of-life weights assigned to various health states, and performance/adherence rates for various interventions.

Executing the Model

Set up the simulation

Using the ARCHeS interface, users define the project population, the interventions, and the target populations for each of the interventions. They define the trial arms they want to compare, specifying the particular interventions that will be given in each trial arm (combinations of interventions are allowed), and specifying the proportion of people in each target population who will actually receive each intervention. These steps are equivalent to designing the population and treatment arms of a clinical trial, or the interventions to be applied in a demonstration program. Users then submit the specifications for calculation.

Run the simulation

When the specifications are received by the Simulator, the calculations begin. At the start of the simulation, the physiology of each person in the project population functions according to the person's baseline characteristics and other variables, and the equations in the physiology model. Each person in the simulation is different, with different baseline characteristics, trajectories for physiological variables, behaviors, random factors, and so forth. If a physiological variable for a particular simulated individual reaches a value at which symptoms occur, the person seeks care, either an outpatient visit or an emergency room visit depending on the symptom and other factors. Simulated people can also make contact with the healthcare system if the project interventions call for that. For example, a project intervention might be to screen people for a disease, in which case a simulated provider in the healthcare system will contact the simulated person and offer the screening. When a simulated person makes contact with the healthcare system, either at the initiative of the healthcare system or the initiative of the person, then simulated providers will follow care processes that are based on guidelines, subject to variations in provider behaviors as described above. The person gets tests and treatments according to the care processes and their own behaviors relating to following recommendations for tests and treatments. Any treatments the person receives can change one or more physiological variables, which in turn can change the progression of the disease, and the occurrence of symptoms and health outcomes. Any interventions specified by the user during the setup of the project will also be applied, either in conjunction with or instead of the background care processes, as defined by the user. During the entire process, all events relating to utilization, costs, health outcomes, and quality of life are recorded. After the simulation is complete and all the results have been recorded, a data file is created and sent back to the user through the ARCHeS interface.

Analyze the results

The user can then use the part of ARCHeS called the "Outcomes Analyzer" to create a wide variety of tables and charts. The user can also change a variety of assumptions, and ARCHeS will immediately recalculate the tables and charts. The number of functions that can be performed with the ARCHeS

© 2011-2013

Outcomes Analyzer is too large to describe here. Access to a demonstration copy of ARCHeS can be obtained by contacting us at archimedesmodel.com/contact.

Validating the Model

The Model is validated for face validity, internal validity, cross-validity, external validity, and predictive validity, using best practices defined by a national task force[6]. Particularly important are the external validations that compare the results of the Model with the results observed in clinical trials, cohort follow-up studies, registries, and large national databases. Historically more than 50 major clinical trials have been used to validate the Model at various stages in its development. Furthermore, particular parts of the Model are continually being validated as part of the model-building process. With ARCHeS, a major part of the validation process has been automated; before the release of each new version of the Model, the Model is validated against a standardized suite of studies using an automated "one-click" process. The methods and results are described in the report "Validation Methodology and Results: ARCHeS Simulator 2.5," available at archimedesmodel.com/resource-center.

Physiology Model Variables

As described above, the core of the Archimedes Model is a collection of algebraic and differential equations that represent the physiological variables and relationships pertinent to diseases and their complications – the physiology model. This section introduces non-technical descriptions the variables and relationships in the equations used in each of the disease sub-models and the sources used to derive the equations. Parties with a serious interest in working with Archimedes can contact us at archimedesmodel.com/contact for additional, quantitative information about the Model.

Identification and Selection of Data Sources

To identify sources for building the disease models we search PubMed and Google Scholar to identify appropriate articles that meet pre-determined criteria based on:

- study design
- number of participants
- patient characteristics (for example, men and women over age 65 with a diagnosis of CVD)
- study duration
- outcomes of interest (for example, MI, angina, or stroke)

Individual PubMed MeSH terms are combined for the search. After the search is performed, each abstract found is reviewed by in-house physicians and analysts to determine if the study meets the inclusion criteria. Studies that meet the criteria are read and evaluated for use in building the Model.

---

[6] Eddy DM, Hollingworth W, Caro JJ, Tsevat J, McDonald KM, Wong JB. Model Transparency and Validation: A Report of the ISPOR-SMDM Modeling Good Research Practices Task Force Working Group - Part 4, Value in Health 2011, in press.

© 2011-2013

References from studies retrieved from the search are also reviewed when appropriate to identify any papers that might not be identified in the initial search. The following aspects of the search are documented for future use:

- search date
- databases searched
- terms used
- number of papers originally found
- number of papers that met the inclusion criteria

Disease Diagrams

The variables and relationships between variables that are used to model the diseases in Simulator 2.5 are illustrated in what we will call "disease diagrams." In the diagrams the rectangles represent variables that in clinical parlance are often called "risk factors" for the disease. Ovals represent variables that are directly related to the disease and its progression. Rectangles with rounded corners represent outcomes of the disease that are experienced by patients such as symptoms and health outcomes. When variables related to the disease affect other organs and other diseases, they are represented in squares. Variables that can be modified with treatments or other inventions are indicated by "Rxs." Variables that can be measured by tests are indicated by "Tests." The arrows represent relationships between the elements, with the direction of the arrow representing the direction of causality. In general, arrows represent equations that relate the variables. None of the elements in these diagrams represent "states" as found in state-transition models, and the arrows do not represent transition probabilities.

Relationships between variables are continuous and dynamic

When viewing these diagrams it is important to bear in mind that virtually all of the variables are continuously valued and changing over time. (The main exceptions are sex and race/ethnicity.) For example, in Figure 4 below, which describes the metabolic changes in type 2 diabetes, the progression toward diabetes (the type 2 diabetes progression function) is a continuous function of a person's weight along with other variables. That is, if a person's weight changes, perhaps due to a diet or exercise program, then the trajectory of the progression function will change to reflect the change in weight. This is very different than, for example, a risk equation such as the Framingham risk equation for MI, which is static in the sense that it takes a person's current values of variables such as age, sex, and SBP, and calculates a ten-year risk of a heart attack from that point. That type of equation does not incorporate information about changes in risk factors in an individual's past or future. In the Archimedes Model variables such as age, SBP, and cholesterol levels are continuously changing, and the progression of the variables that represents plaque and the occurrence of MI are continuously changing with them.

Diabetes Metabolism Model

The variables relating to glucose metabolism and the development of type 2 diabetes are shown in Figure 4. The propensity to develop type 2 diabetes is represented by the type 2 diabetes progression

© 2011-2013

variable. Its associated function, the type 2 diabetes progression function, determines how the propensity to develop the disease progresses as a function of time and other variables. The progression function is different for people of different sexes and races or ethnicities, and depends as well on a person's age, weight (BMI), and family history. The progression function affects the variable in the model that represents the efficiency with which insulin affects plasma glucose levels. It corresponds clinically to how insulin affects the uptake of glucose by fat and muscle. Insulin efficiency is the inverse of insulin resistance. FPG levels are determined by insulin efficiency and by basal hepatic glucose production. The latter is estimated from data on people who do not have diabetes and is a function of age. HbA1c is a function of plasma glucose levels. The chance a person with diabetes will develop a macrovascular or microvascular complication is determined by HbA1c (which affects coronary artery disease (CAD), stroke, nephropathy, retinopathy, and neuropathy), and by the progression function itself (which affects MI, strokes, and nephropathy). The main sources used for this part of the Model are NHANES III (for the progression function), Diabetes in America second edition (for FPG levels), and UKPDS33 (for insulin efficiency). Additional sources are listed in the remainder of this section.

© 2011-2013

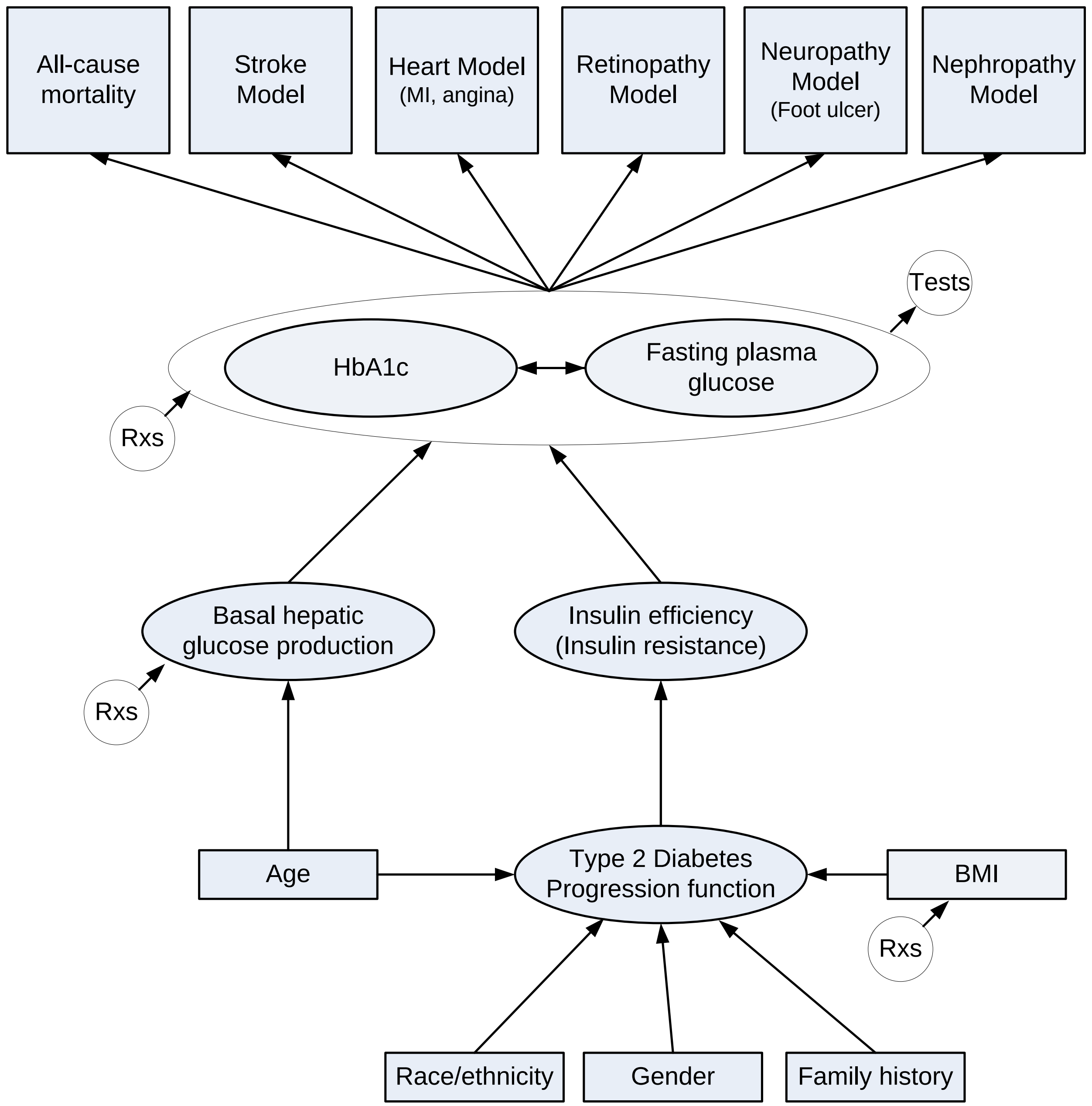

Figure 4. Diagram of Diabetes Metabolism and Pathology.

Type 2 Diabetes Progression Function

The type 2 diabetes progression function determines the probability that an individual will develop type 2 diabetes and the age at which he or she will develop it. Clinically, it corresponds to the “cause” of insulin resistance. The type 2 diabetes progression function is estimated from data on the prevalence of type 2 diabetes at various ages. Diabetes progression functions are calculated separately for men and women and for each of three ethnic groups: white, black, and Hispanic/Mexican American, all using prevalence data from NHANES III. The progression functions for these three groups are also affected by

three other risk factors: BMI, age, and family history. These risk factors are assumed to be independent of sex and race/ethnicity and are incorporated in the equation as relative risks.

The effect of BMI on a person's risk of developing diabetes is based on the following epidemiological studies, which focused on men, women, and extremely obese persons, respectively.

- Field AE et al. Impact of overweight on the risk of developing common chronic diseases during a 10-year period, Arch Intern Med 2001; 161:1581-1586.
- Colditz GA et al. Weight gain as a risk factor for clinical diabetes mellitus in women, Ann Intern Med 1995; 122(7):481-486.
- Sjöström L et al. The Swedish Obese Subjects Study Scientific Group, Lifestyle, diabetes, and cardiovascular risk factors 10 years after bariatric surgery, N Engl J Med 2004; 351:2683-93.

The effect of family history on a person's risk of developing diabetes is based on:

- Grill V et al. Family history of diabetes in middle-aged Swedish men is a gender unrelated factor which associates with insulinopenia in newly diagnosed diabetic subjects, Diabetologia 1999; 42:15-23.
- Meigs JB et al. Parental transmission of type 2 diabetes, The Framingham Offspring Study, Diabetes 2000; 49:2201-2207.
- Millar WJ and Young TK. Tracking diabetes: Prevalence, incidence and risk factors, Health Rep 2003; 14:35-47.
- Harrison TA et al. Family history of diabetes as a potential public health tool, Am J of Prev Med 2003; 24:152-159.

Fasting Plasma Glucose, Insulin Efficiency, and HbA1c

The incidence and progression of type 2 diabetes is characterized by elevated and gradually increasing levels of plasma glucose and FPG, caused by increasing degrees of insulin resistance. The Archimedes Model includes a variable "insulin efficiency" that corresponds to insulin resistance; it is the inverse of insulin resistance. (As resistance to insulin goes up, the efficiency with which insulin controls glucose levels goes down and vice versa). In the Model, insulin efficiency is determined by the type 2 diabetes progression function. As diabetes progresses, insulin efficiency declines. The main source for estimating the relationship between insulin deficiency and FPG is the UKPDS trial. A random component has been added to represent variability among individuals.

In people who do not have diabetes, basal hepatic glucose production and the relationship between FPG and age are based on data from

- Diabetes In America, 2nd edition. National Diabetes Data Group, National Institutes of Health, National Institute of Diabetes and Digestive and Kidney Diseases, NIH Publication No. 95–1468, 1995. (Available at http://diabetes.niddk.nih.gov/dm/pubs/america/index.htm.)

© 2011-2013

HbA1c is determined as a function of FPG based on data from simultaneous measurements of FPG and HbA1c in the Continuous NHANES.

Effects on Other Disease Models

As illustrated in Figure 4, the progression of diabetes affects many organs in the body. These effects are modeled based on the current understanding of the mechanisms by which the disease affects those organs. Diabetic neuropathy and retinopathy are driven by HbA1c levels. Cardiac complications of diabetes, stroke, and kidney function are directly affected by both HbA1c and the diabetes progression function. The type 2 diabetes progression function directly affects overall death rates.

The effect of diabetes on cardiovascular disease is based on many references, the most important of which are:

- The Action to Control Cardiovascular Risk in Diabetes Study Group. Effects of intensive glucose lowering in type 2 diabetes, N Engl J Med 2008; 358:2545-59.
- The ADVANCE Collaborative Group. Intensive blood glucose control and vascular outcomes in patients with type 2 diabetes, N Engl J Med 2008; 358:2560-72.
- Maka KH and Haffnerb SM. Diabetes abolishes the gender gap in coronary heart disease, European Heart Journal 2003; 24(15):1385-1386. (http://eurheartj.oxfordjournals.org/cgi/content/full/24/15/1385).
- Anderson KM et al. Cardiovascular disease risk profiles, American Heart Journal 1991; 121:293-298.
- Coutinho M et al. The relationship between glucose and incident cardiovascular events: A metaregression analysis of published data from 20 studies of 95,783 individuals followed for 12.4 years, Diabetes Care 1999; 22:233–240.
- Howard BV et al. Adverse effects of diabetes on multiple cardiovascular disease risk factors in women. The Strong Heart Study, Diabetes Care 1998; 21(8):1258-65.
- UK Prospective Diabetes Study (UKPDS) Group. Intensive blood-glucose control with sulphonylureas or insulin compared with conventional treatment and risk of complications in patients with type 2 diabetes (UKPDS 33), Lancet 1998; 352(9131):837-53.
- Clarke PM et al. on behalf of the UK Prospective Diabetes Study (UKPDS) Group. A model to estimate the lifetime health outcomes of patients with type 2 diabetes: the United Kingdom Prospective Diabetes Study (UKPDS) outcomes model (UKPDS no. 68), Diabetologia 2004; 47:1747–1759.
- Coleman RL et al. Framingham, SCORE, and DECODE risk equations do not provide reliable cardiovascular risk estimates in type 2 diabetes, Diabetes Care 2007; 30:1292-1293. (http://care.diabetesjournals.org/cgi/content/full/30/5/1292.)

The effects of diabetes on development of kidney disease, stroke, and mortality are discussed below.

Diabetic Retinopathy Model

The variables in the diabetic retinopathy model and their relationships are illustrated in Figure 5. In people with diabetes the first manifestation of diabetic retinopathy is non-proliferative retinopathy. The disease can then progress to proliferative retinopathy and blindness. The progression variables that determine whether and when a person with diabetes will develop retinopathy and the rate at which it will progress are determined by the duration of diabetes (defined as time since FPG > 125 mg/dL), SBP, and HbA1c level. (In the Archimedes Model, unless specifically stated otherwise, duration of diabetes is always defined as time since FPG > 125 mg/dL. Notice that this is different from onset of symptoms or first diagnosis.) The rate at which proliferative diabetic retinopathy (PDR) will develop is a function of how long the person has had non-proliferative diabetic retinopathy (NPDR). The occurrence of unilateral or bilateral blindness is determined by the progression of PDR. A patient with NPDR can also develop macular edema, with the progression to that condition affected by how long the patient has had non-proliferative diabetic retinopathy, as well as the patient's SBP and LDL cholesterol.

© 2011-2013

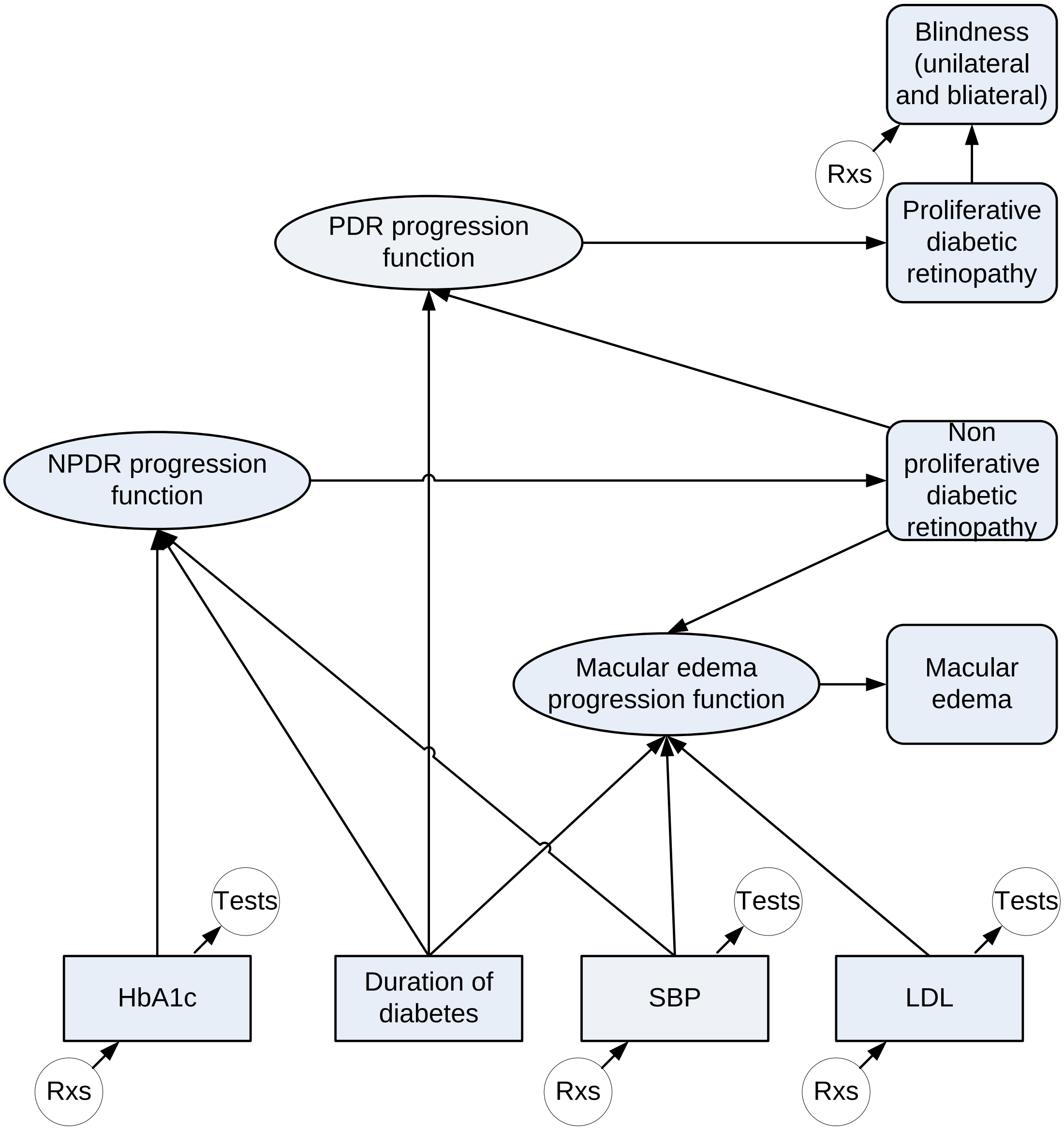

Figure 5. Diagram of Diabetic Retinopathy.

Risk Factors for Non-Proliferative Diabetic Retinopathy

Risk factors in the Model for NPDR are blood glucose levels (represented by HbA1c), SBP, duration of diabetes, and diabetes type (type 1 or type 2). The risk of developing NPDR as a function of HbA1c is based on data from the following five studies:

- Harris M et al. Is the risk of diabetic retinopathy greater in non-Hispanic blacks and Mexican Americans than in non-Hispanic whites with type 2 diabetes?, Diabetes Care 1998; 21(8)1230-1235.
- Haffner SM et al. Diabetic retinopathy in Mexican Americans and non-Hispanic whites, Diabetes 1988; 37(7):878-84.

- Tapp RJ et al. The prevalence of and factors associated with diabetic retinopathy in the Australian population, Diabetes Care 2003; 26(6):1731-7.
- Brown JB et al. Diabetic retinopathy: contemporary prevalence in a well-controlled population, Diabetes Care 2003; 26(9):2637-42.
- Klein R et al. Relationship of hyperglycemia to the long-term incidence and progression of diabetic retinopathy, Arch Intern Med 1994; 154(19):2169-78.

The effect of SBP levels on the risk of developing NPDR is based on data from the first four papers listed above, plus the following two papers:

- Klein R et al, DeMets DL. Is blood pressure a predictor of the incidence or progression of diabetic retinopathy? Arch Intern Med 1989; 149(11):2427-32.
- Adler AI et al. Association of systolic blood pressure with macrovascular and microvascular complications of type 2 diabetes (UKPDS 36): prospective observational study, BMJ 2000; 321(7258):412-9.

The risk of people with type 2 diabetes developing NPDR is based on data from:

- Klein R et al. The Wisconsin epidemiologic study of diabetic retinopathy. III. Prevalence and risk of diabetic retinopathy when age at diagnosis is 30 or more years, Arch Ophthalmol 1984; 102(4):527-32.
- Brown JB et al. Diabetic retinopathy: contemporary prevalence in a well-controlled population, Diabetes Care 2003; 26(9):2637-42.

Risk Factors for Proliferative Diabetic Retinopathy

The Model assumes that no one develops PDR without first developing NPDR. The risk of developing PDR, given that the individual has already developed NPDR, depends only on the duration of diabetes.

The risk of developing PDR for people with type 2 diabetes is based on:

- Klein R et al. The Wisconsin epidemiologic study of diabetic retinopathy. III. Prevalence and risk of diabetic retinopathy when age at diagnosis is 30 or more years, Arch Ophthalmol 1984; 102(4):527-32.
- Brown JB et al. Diabetic retinopathy: contemporary prevalence in a well-controlled population, Diabetes Care 2003; 26(9):2637-42.

Risk Factors for Diabetic Macular Edema

As with PDR, no one will develop macular edema without first developing NPDR. The risk of developing macular edema, given that the individual has already developed NPDR, depends on duration of diabetes, SBP, and LDL cholesterol. The development of macular edema as a function of LDL cholesterol level is based on data from the following paper:

© 2011-2013

- Rema M et al. Association of serum lipids with diabetic retinopathy in urban South Indians--the Chennai Urban Rural Epidemiology Study (CURES) Eye Study—2, Diabet Med 2006; 23(9):1029-36.

The risk of developing macular edema as a function of SBP level is based on:

- Brown JB et al. Diabetic retinopathy: contemporary prevalence in a well-controlled population, Diabetes Care 2003; 26(9):2637-42.

The relationship between the duration of diabetes and the development of macular edema is based on:

- Klein R et al. The Wisconsin epidemiologic study of diabetic retinopathy. IV. Diabetic macular edema, Ophthalmology 1984; 91(12):1464-74.
- Klein R et al. The Wisconsin Epidemiologic Study of diabetic retinopathy. XIV. Ten-year incidence and progression of diabetic retinopathy, Arch Ophthalmol 1994; 112(9):1217-28.
- Brown JB et al. Diabetic retinopathy: contemporary prevalence in a well-controlled population, Diabetes Care 2003; 26(9):2637-42.

Blindness

The rate at which people who have been diagnosed with PDR lose vision in one or both eyes depends strongly on whether the person has had laser treatment, which is included explicitly in the care processes in the Model. The rates of blindness in patients with type 2 diabetes are based on data from:

- Klein R et al. Visual impairment in diabetes, Ophthalmology 1984; 91(1):1-9.

Diabetic Neuropathy Model

The variables in the diabetic neuropathy model are shown in Figure 6. The model includes three main conditions: sensory neuropathy, foot ulcers, and amputations. Each has its own progression variable and progression function, and each is determined by the patient's HbA1c. In addition, amputation is affected by a person's sex as well as the person's level of proteinuria and the length of time a person has had diabetes (FPG > 125 mg/dL). The development of foot ulcers is a function of proteinuria and duration of diabetes as well as HbA1c. The development of sensory neuropathy is a function of HbA1c and the person's age and height. The dependence on duration of diabetes reflects the fact that foot ulcers occur more frequently in individuals who have had diabetes longer.

© 2011-2013

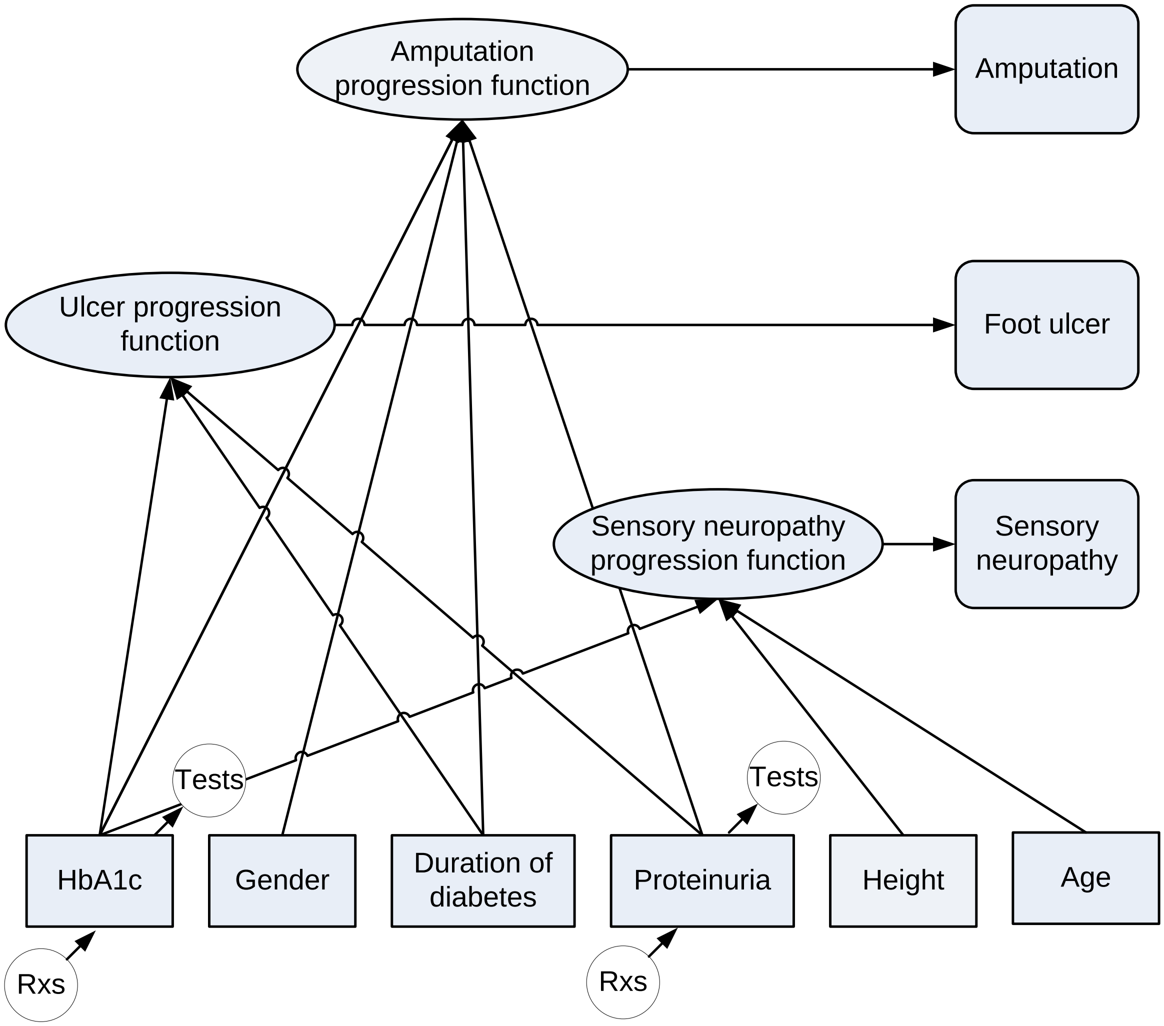

Figure 6. Diagram of Diabetic Neuropathy.

Risk Factors for Sensory Neuropathy

The risk of sensory neuropathy as a function of age, height, and HbA1c is based on:

- Adler AJ et al. Risk factors for diabetic peripheral sensory neuropathy: results of Seattle Prospective Diabetic Foot Study, Diabetes Care 1997; 20(7).

Risk Factors for Foot Ulcer

The risk of developing foot ulcers as a function of HbA1c, duration of diabetes, and proteinuria is based on:

- Moss SE et al. The prevalence and incidence of lower extremity amputation in diabetic population, Arch Intern Med 1992; 152:610-616.

Foot ulcers can recur after treatment. The recurrence rate is based on:

- Apelqvist J et al. Long-term prognosis of diabetic patients with foot ulcers, J Intern Med 1993; 233:485-491.

Risk Factors for Amputation

Risk factors for lower limb amputation are blood glucose levels as measured by HbA1c, duration of diabetes, proteinuria, sex, and history of foot ulcer. A total of eight amputation sites are used in the Model: toe, foot/ankle, below knee, and above knee for each leg. The function for amputation and amputation recurrence rate are based on the sources listed above for foot ulcer.

Nephropathy Model

The nephropathy influence diagram is shown in Figure 7. The model provides four outcomes: long-term persistent microalbuminuria, long-term persistent macroalbuminuria, stage 3 chronic kidney disease (CKD3), and end-stage renal disease (ESRD). Long-term persistence means that remission is not possible – that the person will have the outcome for life. The four outcomes are determined by disease progression functions in the Archimedes Model. In addition, the model involves two important biomedical variables that measure renal function: the urinary albumin-to-creatinine ratio (UACR) and the glomerular filtration rate (GFR). A person must develop microalbuminuria before developing macroalbuminuria. Similarly, CKD3 must occur before ESRD. The model also includes the possibility of a kidney transplant for treatment of ESRD, and an increased overall death rate for individuals diagnosed with ESRD.

For micro- and macro-albuminuria, the risk factors in the model are age, gender, race/ethnicity, diabetes status, HbA1c, systolic blood pressure, and smoking. For CKD3, they are age, gender, race/ethnicity, diabetes status and UACR. For ESRD, the risk factors are age, gender, race/ethnicity and UACR. Although HbA1c, systolic blood pressure, and smoking do not directly affect CKD3 and ESRD, they still have an indirect effect on these outcomes through UACR.

The UACR trajectory not only affects CKD3 and ESRD but also affects macroalbuminuria in a two-way relationship: the progression function for macroalbuminuria is affected by UACR, and the UACR trajectory is determined by the progression functions for macroalbuminuria (and also microalbuminuria). Similarly, there is a two-way relationship between the GFR trajectory and the CKD3 and ESRD disease models.

A person is diagnosed with CKD3 if his or her GFR test result is below 60 mL/min (per 1.73 m^2). ESRD is diagnosed as the need for renal replacement therapy (either dialysis or kidney transplant). Microalbuminuria and macroalbuminuria are diagnosed if UACR is measured to be above 30 mg/g and 300 mg/g respectively.

The nephropathy model is primarily based on the following datasets and publications:

- NHANES 1999-2008.

- United States Renal Data System (USRDS), USRDS 2011 Annual Data Report: Atlas of Chronic Kidney Disease and End-Stage Renal Disease in the United States, National Institutes of Health, National Institute of Diabetes and Digestive and Kidney Diseases, Bethesda, MD, 2011.
- The Chronic Kidney Disease Prognosis Consortium:
  - Gansevoort RT, Matsushita K, van der Velde M, Astor BC, Woodward M, Levey AS, de Jong PE, Coresh J; Chronic Kidney Disease Prognosis Consortium. Lower estimated GFR and higher albuminuria are associated with adverse kidney outcomes. A collaborative meta-analysis of general and high-risk population cohorts. Kidney Int. 2011; 80:93-104.
  - Astor BC, Matsushita K, Gansevoort RT, van der Velde M, Woodward M, Levey AS, Jong PE, Coresh J; Chronic Kidney Disease Prognosis Consortium, Lower estimated glomerular filtration rate and higher albuminuria are associated with mortality and end-stage renal disease. A collaborative meta-analysis of kidney disease population cohorts. Kidney Int. 2011; 79:1331-40.

© 2011-2013

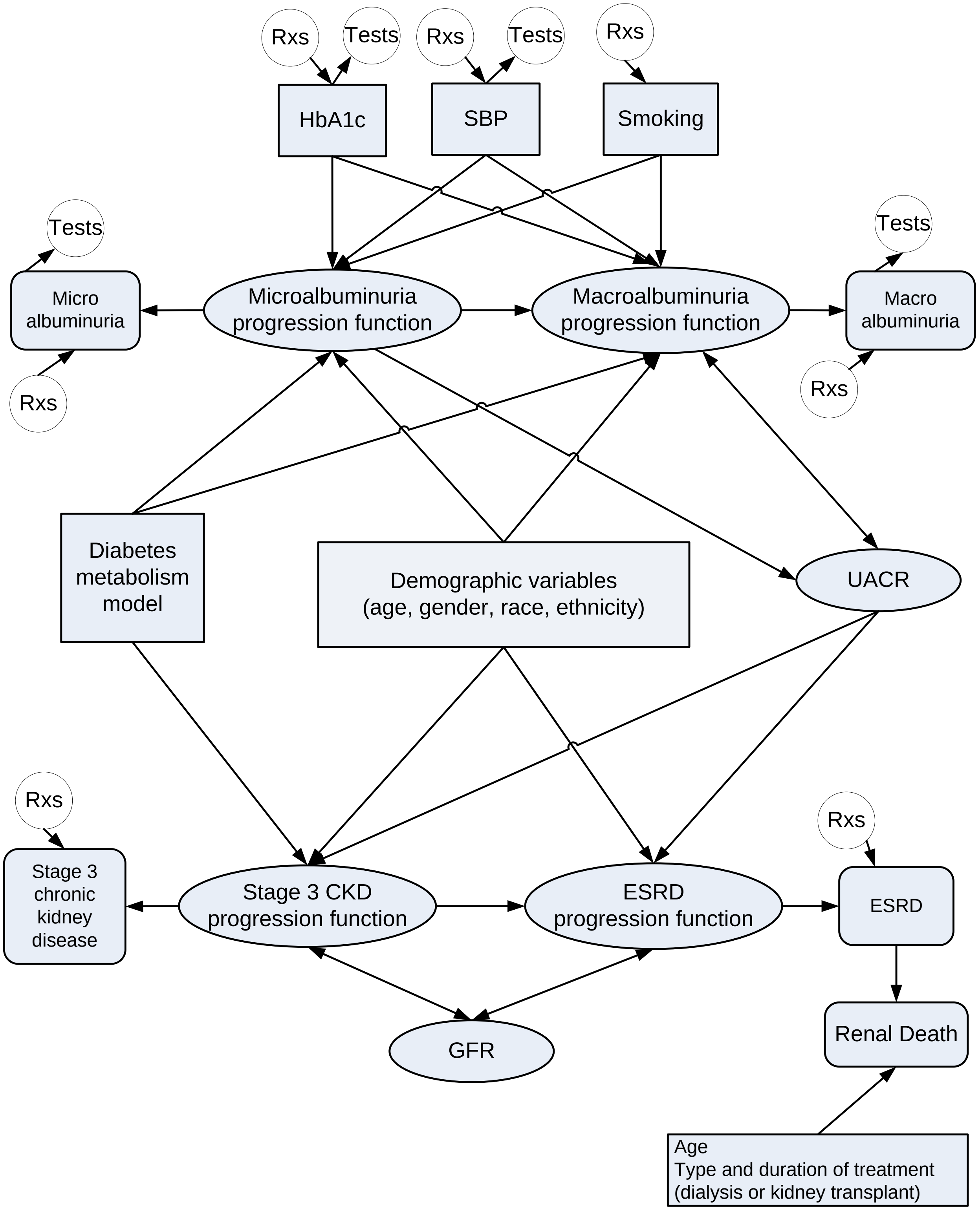

Figure 7. Diagram of Diabetic Nephropathy.

Coronary Artery Disease Model

The coronary artery disease (CAD) model is illustrated in Figure 8. It is driven by three progression functions that cause people to get stable angina, unstable angina, and MI. Stable angina corresponds clinically to occlusive plaque which causes symptoms well before complete occlusion and MI. It is measured by tests such as angiograms and treated by procedures such as PTCA and bypass grafts. Unstable angina corresponds to unstable plaque, which causes intermittent angina symptoms and indicates a high risk of MI due to sudden occlusion. Tests such as carotid intima-medial thickness (CIMT) indicate a person's risk of unstable plaque. The MI progression function determines the occurrence of an occlusive event such as rupture of plaque, clotting, and occlusion of the coronary artery. In the Model an MI can occur in any of the coronary arteries, and at any place within any artery. The proportions of MIs occurring at particular spots in particular arteries are set to match observed frequencies. Depending on the artery and the spot in the artery at which the occlusion occurs, the myocardium can be deprived of blood, causing symptoms and reduction in cardiac output. Because stable angina, unstable angina, and MI can all occur independently of each other, and because there is no progression from one to the other, they are represented in the Archimedes Model with their own progression functions. However, their occurrences are highly correlated because they share the same risk factors, and the presence of one implies (but does not cause) an increased risk of occurrence of the others. The correlations between the three types of coronary artery events is captured in the equations that calculate the progression functions for each event as functions of risk factors they share.

Person-specific data from two main sources are used to write the equations for the progression functions. Data from Atherosclerosis Risk in Communities (ARIC) are used to write progression functions for first MI, stable angina, and unstable angina. Data from the Framingham Heart Study (FHS) are used to write progression functions for first MI and recurrent MIs. Information about these datasets is in the following references:

- The ARIC Investigators. The Atherosclerosis Risk in Communities (ARIC) Study: design and objectives, Am J Epidemiology 1989; 129(4):687-702.
- McGee D. The Framingham Study: An epidemiologic investigation of cardiovascular disease, Section 27. Bethesda, MD: US Government Printing Office, 1973.
- Anderson KM et al. Cardiovascular disease risk profiles, American Heart Journal 1991; 121(1):293-298.

© 2011-2013

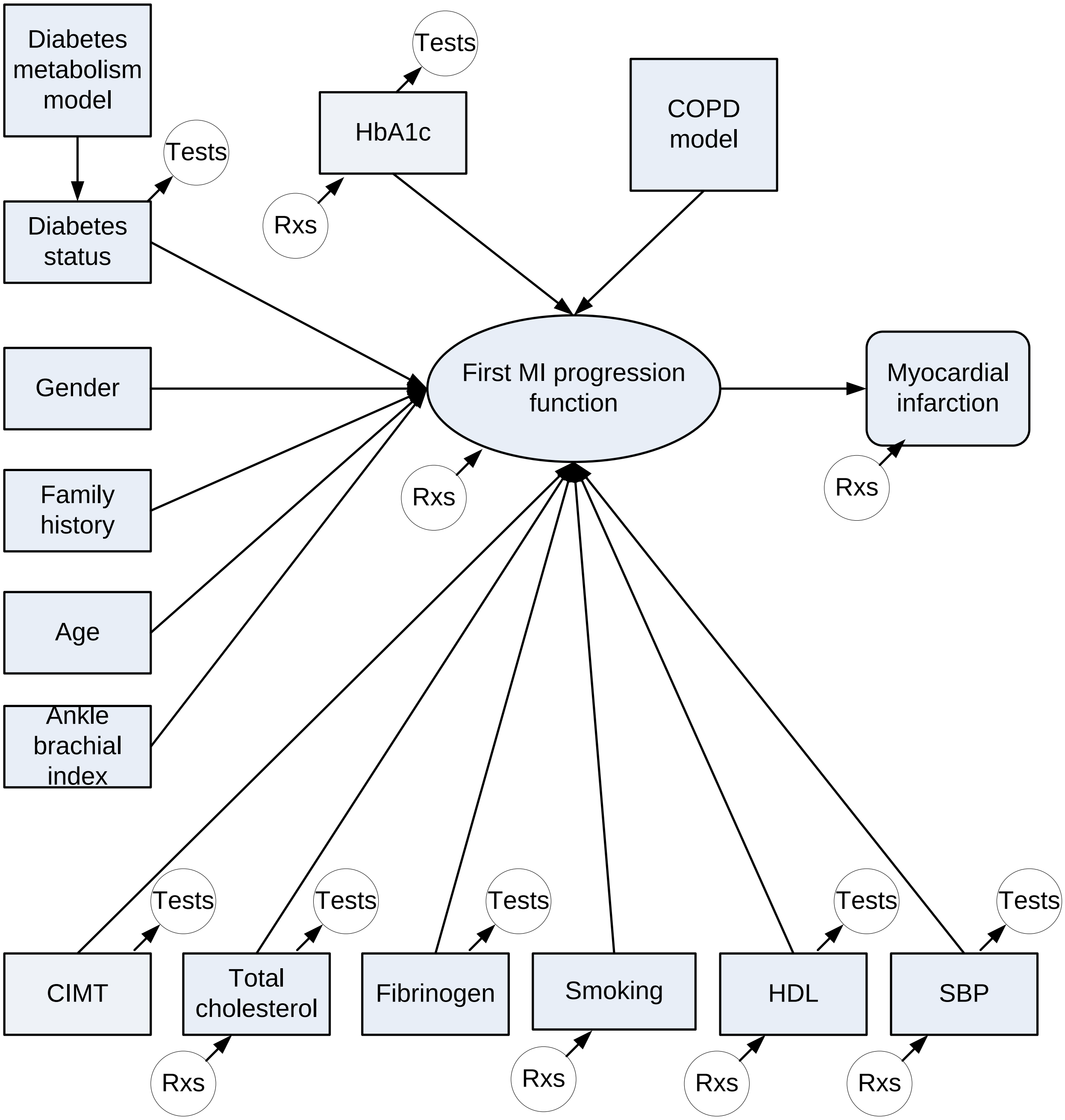

Figure 8. Diagram of First MI.

Risk Factors for First Myocardial Infarction

Risk factors for a person's first MI are age, sex, SBP, smoking status, HbA1c, family history, total cholesterol, HDL cholesterol, fibrinogen, ankle-brachial index (ABI), and CIMT. An additional risk factor is the presence of diabetes; this factor is calculated separately for men and women. Risk factors for first MI are based on the ARIC and Framingham datasets, cited above. Recurrent MI depends on time since

the previous MI and diabetic status. Risk factors for recurrent MI are based on the Framingham and ARIC datasets, cited above.

Risk Factors for Unstable Angina

The risk factors for unstable angina are the same as those for first MI and are also based on the ARIC dataset.

Risk Factors for Stable Angina

Risk factors for stable angina are age, sex, smoking status, family history, total cholesterol, HDL cholesterol, presence and severity of diabetes (calculated from the type 1 and type 2 diabetes progression function and FPG), waist-to-hip ratio (WHR), ABI, and CIMT. The progression function for stable angina is estimated using the ARIC dataset.

© 2011-2013

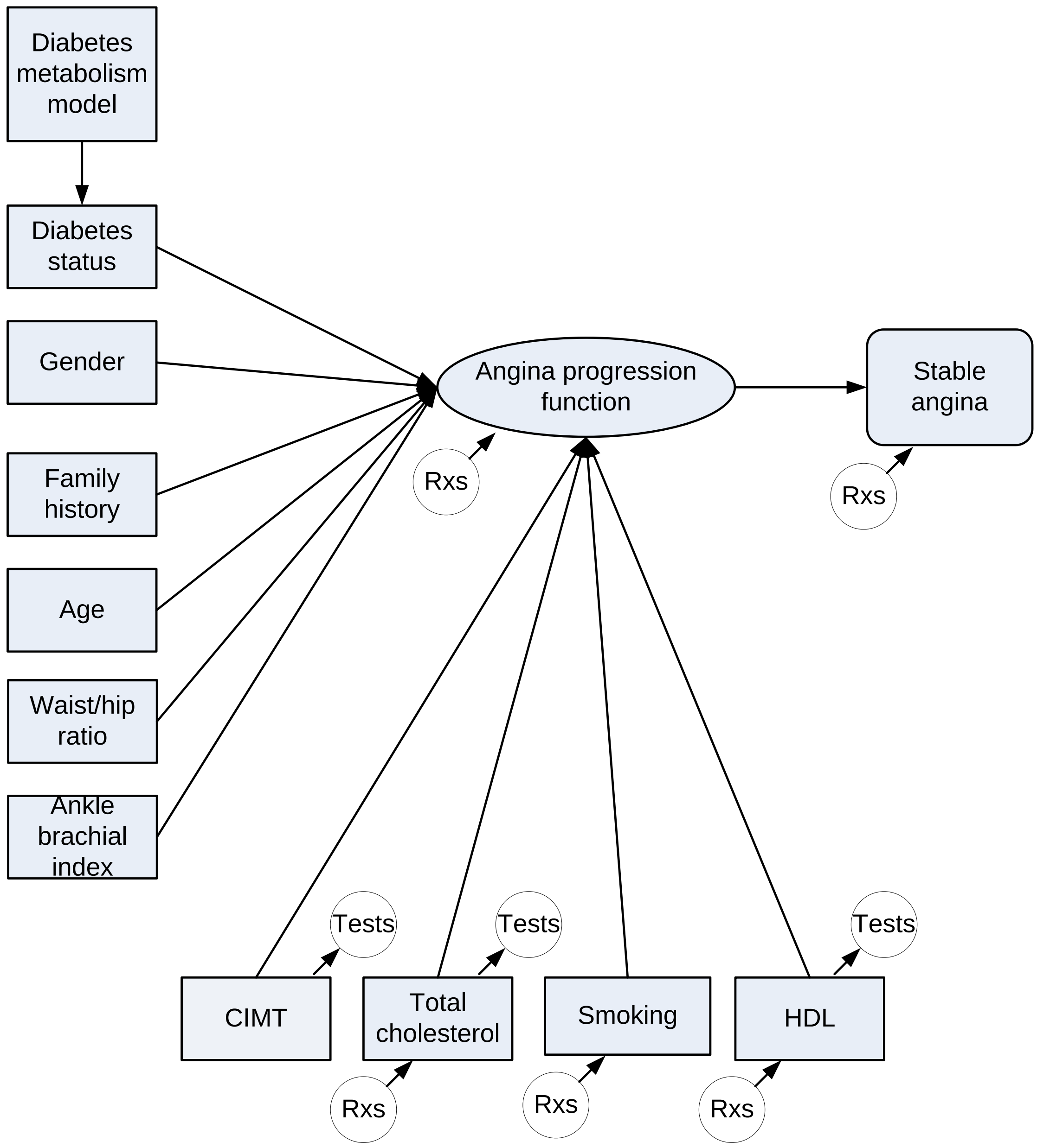

Figure 9. Diagram of Stable Angina.

Heart Failure Model

In the model, heart failure can be directly caused by a myocardial infarction, or can occur with no direct cause. Risk factors for heart failure caused by MI are age, time since MI, and left ventricular ejection fraction. Risk factors for heart failure without direct cause are age, diabetic status, fasting plasma glucose, body surface area, history of angina, history of chronic atrial fibrillation, number of previous

MIs, smoking status, and unmedicated systolic blood pressure. Antihypertensive medications and statins also affect the risk of heart failure.

Risk factors for acute heart failure death (within 30 days of a heart failure event) include age, sex, number of previous heart failure events, body surface area, and history of stroke.

Risk factors for recurrent heart failure are age, diabetic status, unmedicated systolic blood pressure, number of previous heart failure events, and time since last heart failure event. Risk of recurrent heart failure is highest immediately after a heart failure episode and decreases over time.

Patients with heart failure are at elevated risk for death from any cause relative to individuals of similar demographics without heart failure. The heart failure model incorporates this added risk of death. Risk factors are age, sex, left ventricular ejection fraction, history of stroke, and time since the last heart failure event. As with recurrent heart failure, the elevated risk of death from any cause is highest immediately after a heart failure episode and decreases over time.

The heart failure model is based on person-specific data from the Cardiovascular Health Study (CHS), along with aggregated clinical trial data from the additional references listed below.

- Fried LP, Borhani NO, Enright P, et al. The Cardiovascular Health Study (CHS): Design and Rationale. Annals of Epidemiology 1991; 1:263-76.
- Butler J et al. Incident heart failure prediction in the elderly: the health ABC heart failure score, Circ Heart Fail 2008; 1(2):125-133.
- Butler et al. Systolic blood pressure and incident heart failure in the elderly. The Cardiovascular Health Study and the Health, Ageing and Body Composition Study, Heart 2011; 97:1304-1311.
- de Simone G et al. Left ventricular mass predicts heart failure not related to previous myocardial infarction: the Cardiovascular Health Study, Eur Heart J 2008; 29:741-747.
- Gottdiener JS et al. Predictors of congestive heart failure in the elderly: the Cardiovascular Health Study, J Am Coll Cardiol 2000; 35(6):1628 -1637.
- Kalogeropoulos A et al. Cardiovascular Health Study. Validation of the Health ABC heart failure model for incident heart failure risk prediction: the Cardiovascular Health Study, Circ Heart File 2010; 3:495-502.
- Kannel WB et al. Profile for estimating risk of heart failure, Arch Intern Med 1999; 159(11):1197-1204.
- Lloyd-Jones DM et al. Lifetime risk for developing congestive heart failure, Circulation 2002; 106:3068-3072.
- Levy D et al. The progression from hypertension to congestive heart failure, JAMA 1996; 275(20):1557-1562.

 ARCHIMEDES © 2011-2013

- Velagaleti RS et al. Long-term trends in the incidence of heart failure after myocardial infarction, Circulation 2008; 118:2057-2062.

The following reference was used to develop the model of the effects of antihypertensive medications on heart failure:

- Sciarretta et al. Antihypertensive Treatment and Development of Heart Failure in Hypertension, Arch Intern Med 2011; 171(5): 384-394.

The following reference was used to develop the model of the effects of statins on heart failure:

- Scirica BM et al. Intensive statin therapy and the risk of hospitalization for heart failure after an acute coronary syndrome in the PROVE IT-TIMI 22 study, J Amer Coll Cardiol 2006; 47(11):2326-2331.

Chronic Atrial Fibrillation Model

Chronic atrial fibrillation is important not only as a condition in its own right, but also because of the risk it imposes for ischemic stroke. The chronic atrial fibrillation model is based on data from the Cardiovascular Heath Study (CHS).

The occurrence of chronic atrial fibrillation is determined by the chronic atrial fibrillation progression function, which is a function of age, systolic blood pressure (SBP), history of CHF, MI, or angina, and height (Figure 10).

© 2011-2013

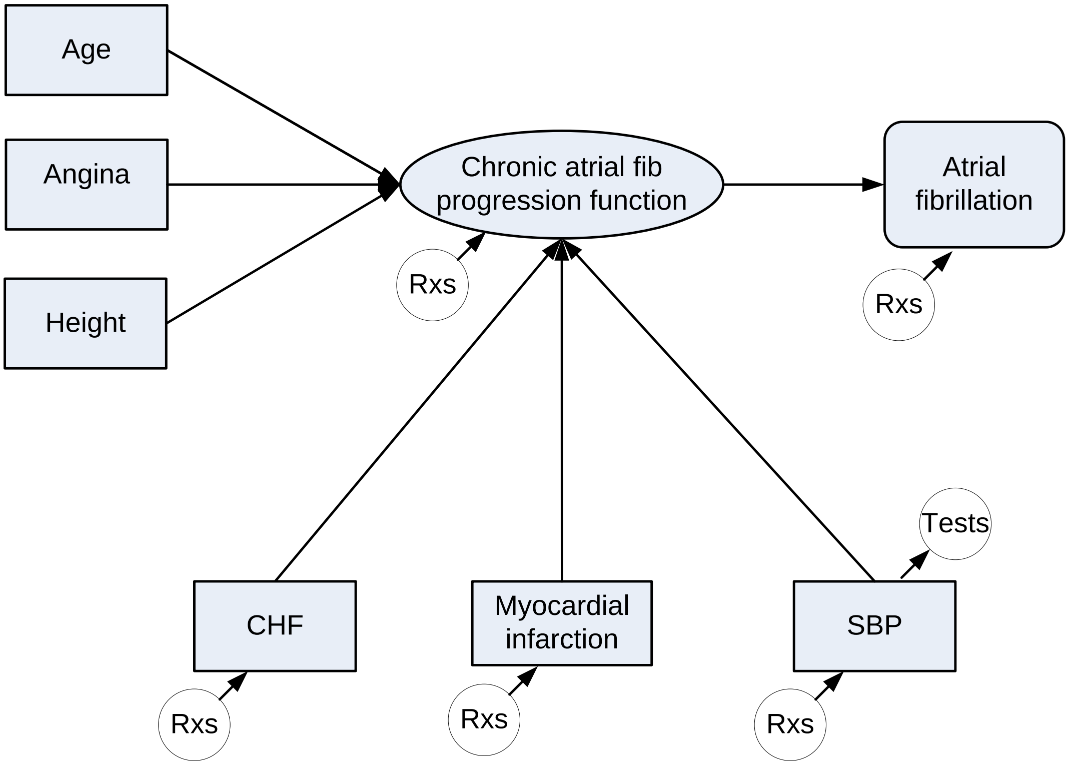

Figure 10. Diagram of Chronic Atrial Fibrillation.

The progression function for chronic atrial fibrillation is based on:

- Fried LP et al. The Cardiovascular Health Study (CHS): Design and Rationale, Annals of Epidemiology 1991; 1:263-276.

Lipids Model

The Archimedes Model includes a physiologically motivated model of cholesterol and triglyceride levels (Figure 11). Triglycerides (TG) depend on age, sex, and the severity of type 2 diabetes. The effect of diabetes on TGs is modeled using information about the relationship between TGs and FPG in a large dataset from a health maintenance organization. TGs affect the trajectories for HDL and total cholesterol (TC). LDL cholesterol is calculated using the Friedewald equation: TC= HDL+LDL+TG/5.

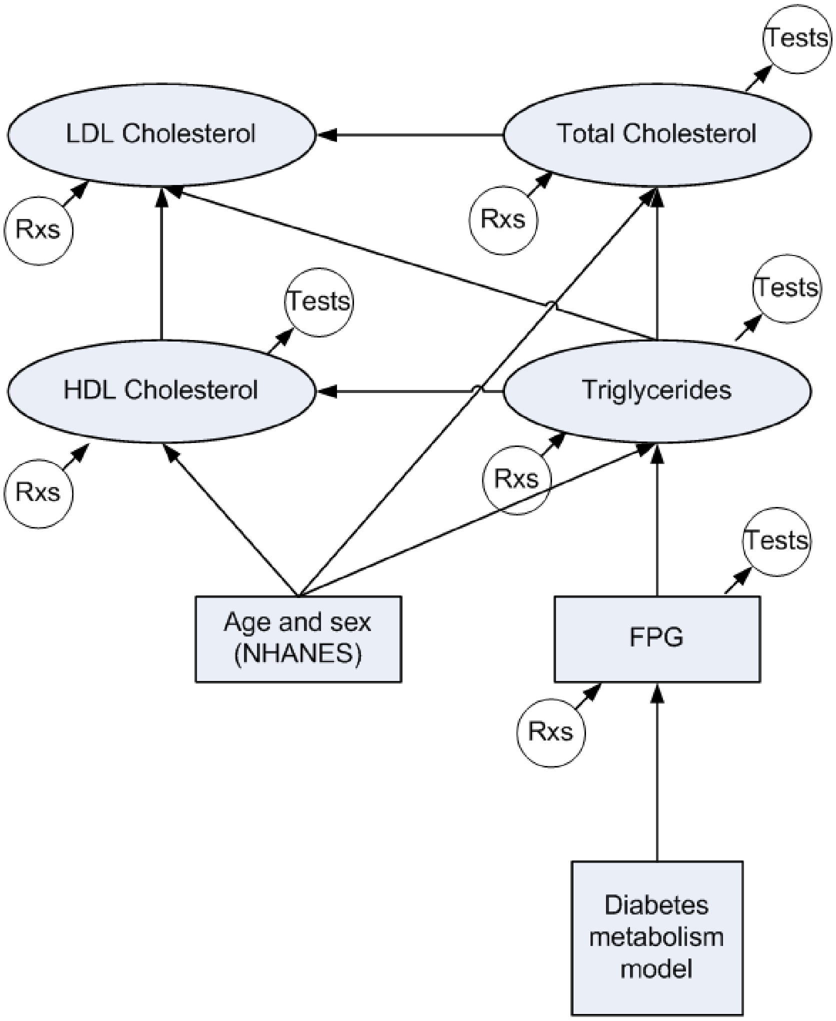

Figure 11. Diagram of the Lipids Model.

Stroke Model

The Archimedes stroke model includes two types of stroke: ischemic stroke, caused by a blocked blood vessel in the brain, and hemorrhagic stroke, caused by bleeding into the brain. The model is based on a single progression function that is multiplied by the relative probability of ischemic and hemorrhagic strokes.

Because the probability distribution for subsequent strokes is different from that of initial strokes, the model uses separate progression functions for “first stroke” and “recurrent stroke.”

First Stroke

The progression function for first stroke depends on age, systolic blood pressure, sex, smoking status, diabetes status, HbA1c, MI history, angina history, and presence of atrial fibrillation (Figure 12). Progression functions for first stroke are determined based on:

- Black HR et al. Principal results of the Controlled Onset Verapamil Investigation of Cardiovascular End Points (CONVINCE) Trial. JAMA 2003; 289:2073-2082.
- Brown MJ et al. Morbidity and mortality in patients randomised to double-blind treatment with a long-acting calcium-channel blocker or diuretic in the International Nifedipine GITS study: Intervention as a Goal in Hypertension Treatment (INSIGHT). Lancet 2000; 356:366-72.
- Chalmers J et al. Intensive blood glucose control and vascular outcomes in patients with type 2 diabetes (ADVANCE). N Engl J Med 2008; 358.
- Dahlöf B et al. Cardiovascular morbidity and mortality in the Losartan Intervention For Endpoint reduction in hypertension study (LIFE): a randomised trial against atenolol. Lancet 2002; 359:995-1003.
- Dahlof B et al for the ASCOT investigators. Prevention of cardiovascular events with an antihypertensive regimen of amlodipine adding perindopril as required versus atenolol adding bendroflumethiazide as required, in the Anglo-Scandinavian Cardiac Outcomes Trial (ASCOT) - Blood Pressure Lowering Arm: a multicentre randomised controlled trial. Lancet 2005; 366:895-906.
- Gerstein H et al. Effects of intensive glucose lowering in type 2 diabetes (ACCORD). N Engl J Med 2008; 358.
- Hansson L et al. Randomised trial of effects of calcium antagonists compared with diuretics and β-blockers on cardiovascular morbidity and mortality in hypertension: the Nordic Diltiazem (NORDIL) study. Lancet 2000; 356:359-65.
- Hansson L et al. Randomised trial of old and new antihypertensive drugs in elderly patients: cardiovascular mortality and morbidity the Swedish Trial in Old Patients with Hypertension-2 (STOP-2) Study. Lancet 1999; 354:1751-6.
- Hansson L et al. Effect of angiotensin-converting-enzyme inhibition compared with conventional therapy on cardiovascular morbidity and mortality in hypertension: the Captopril Prevention Project (CAPPP) randomised trial. Lancet 1999; 353:611-16.
- Hansson L et al for the HOT Study Group. Effects of intensive blood-pressure lowering and low-dose aspirin in patients with hypertension: principal results of the Hypertension Optimal Treatment (HOT) randomised trial. Lancet 1998; 351:1755-62.
- Hu FB et al. Elevated risk of cardiovascular disease prior to clinical diagnosis of type 2 diabetes (NHS). Diabetes care 2002; 25:1129-34.
- Julius S et al for the VALUE (Valsartan Antihypertensive Long-term Use Evaluation) trial group. Outcomes in hypertensive patients at high cardiovascular risk treated with regimens based on valsartan or amlodipine: the VALUE randomised trial. Lancet 2004; 363:2022-31.

© 2011-2013

- Khaw KT et al. Glycated haemoglobin, diabetes, and mortality in men in Norfolk cohort of European Prospective Investigation of Cancer and Nutrition (EPIC-Norfolk). Bmj 2001; 322:15.
- Lithell H et al for the SCOPE study group. The Study on Cognition and Prognosis in the Elderly (SCOPE). J Hypertens 2003; 21:875-86.
- Lui L et al for the FEVER Study Group. The felodipine event reduction (FEVER) Study: a randomized long-term placebo-controlled trial in Chinese hypertensive patients. J Hypertens 2005; 23:2157-72.
- Medical Research Council (MRC) Working Party. MRC trial of treatment of mild hypertension: principal results. BMJ 1985; 291:97-104.
- Medical Research Council (MRC) Working Party. MRC trial of treatment of hypertension in older adults: principal results. BMJ 1992; 304:405-12.
- Nakamura H et al for the MEGA Study Group. Primary prevention of cardiovascular disease with pravastatin in Japan (MEGA Study): a prospective randomised controlled trial. Lancet 2006; 368: 1155–63.
- Pepine CJ et al for the INVEST (International Verapamil-Trandolapril Study) Investigators. A calcium antagonist vs a non-calcium antagonist hypertension treatment strategy for patients with coronary artery disease. JAMA 2003;290:2805-16
- Scandinavian Simvastatin Survival Study Group. Randomised trial of cholesterol lowering in 4444 patients with coronary heart disease: the Scandinavian Simvastatin Survival Study (4S). Lancet 1994; 344: 1383–89.
- Sever PS et al. Prevention of coronary and stroke events with atorvastatin in hypertensive patients who have average or lower-than-average cholesterol concentrations, in the Anglo-Scandinavian Cardiac Outcomes Trial-Lipid Lowering Arm (ASCOT-LLA): a multicentre randomised controlled trial. Lancet 2003; 361: 1149–58.
- Shepherd J et al on behalf of the PROSPER study group. Pravastatin in elderly individuals at risk of vascular disease (PROSPER): a randomised controlled trial. Lancet 2002; 360: 1623–30.
- Shepherd J et al. Prevention of coronary heart disease with pravastatin in men with hypercholesterolemia (WOSCOPS). N Engl J Med 1995; 333: 1301–07.
- Staessen JA et al for the Systolic Hypertension in Europe (Syst-Eur) Trial Investigators. Randomised double-blind comparison of placebo and active treatment for older patients with isolated systolic hypertension. Lancet 1997; 350:757-64.
- Study of the Eff ectiveness of Additional Reductions in Cholesterol and Homocysteine (SEARCH) Collaborative Group. Intensive lowering of LDL cholesterol with 80 mg versus 20 mg simvastatin daily in 12,064 survivors of myocardial infarction: a double-blind randomised trial. Lancet 2010; published online Nov 9. DOI:10.1016/S0140-6736(10)60310-8.
- Sunaga K et al. Glycated Hemoglobin and Risk of Stroke, Ischemic and Hemorrhagic, in Japanese Men and Women (Toyama City). Cerebrovascular Diseases 2008; 26:310-6.
- Sung J et al. Fasting blood glucose and the risk of stroke and myocardial infarction (KNHIS). Circulation 2009; 119:812-9.

© 2011-2013

- The Long-Term Intervention with Pravastatin in Ischaemic Disease (LIPID) Study Group. Prevention of cardiovascular events and death with pravastatin in patients with coronary heart disease and a broad range of initial cholesterol levels. N Engl J Med 1998; 339: 1349–57.
- Wikstrand J et al. Metoprolol versus thiazide diuretics in hypertension: morbidity results from the MAPHY study. Hypertension 1991; 17:579-88.
- Wilhelmsen L et al on behalf of the Heart Attack Primary Prevention in Hypertension (HAPPY) Trial Research Group. Beta-blockers versus diuretics in hypertensive men: main results from the HAPPHY trial. J Hypertens 1987; 5:561-72.
- Wing LMH et al for the second Australian national blood pressure study group. A comparison of outcomes with angiotensin-converting-enzyme inhibitors and diuretics for hypertension in the elderly. N Eng J Med 2003; 348:583-92.

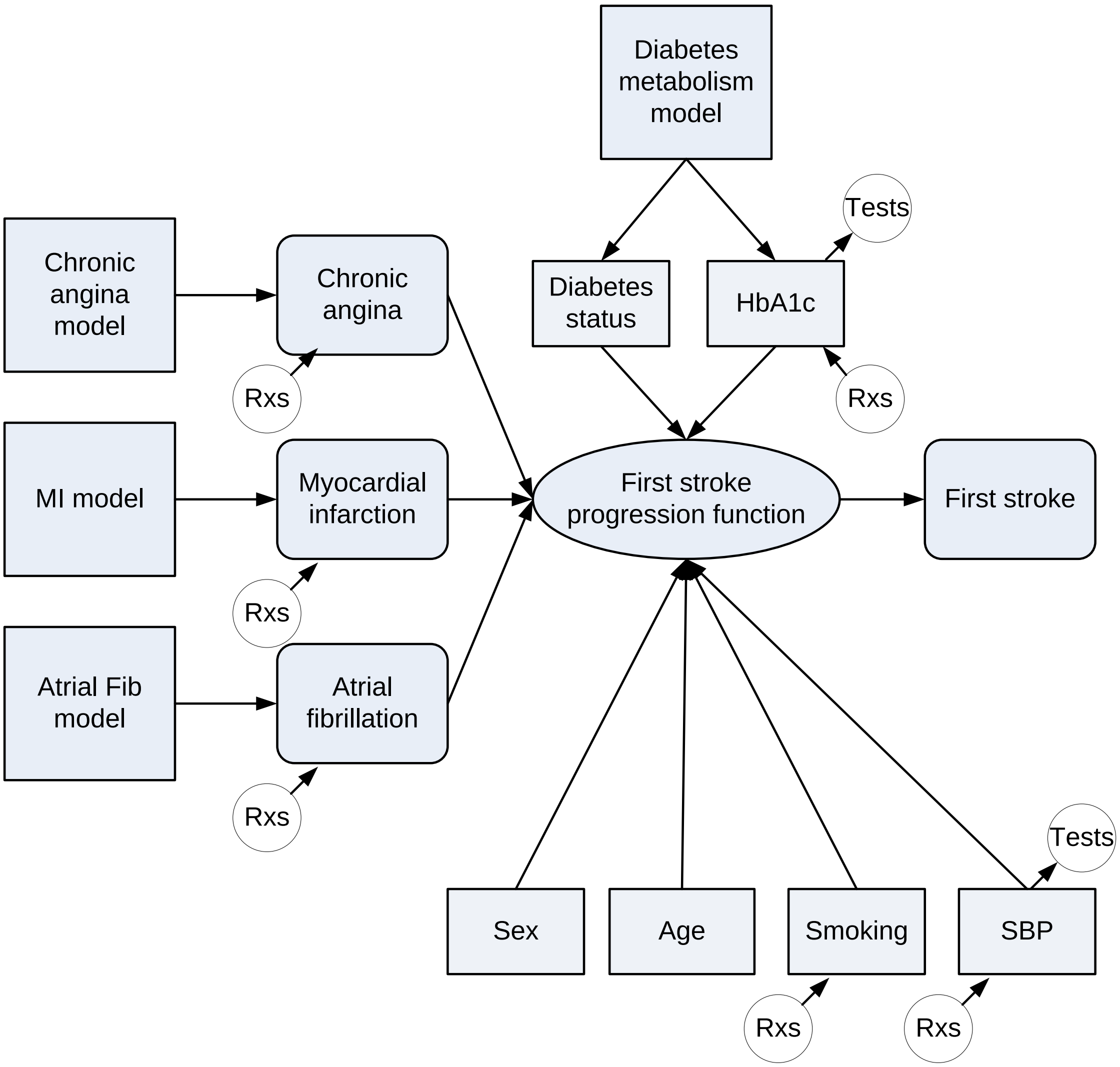

Figure 12. Diagram of First Stroke Model.

ARCHIMEDES
© 2011-2013

Recurrent Stroke

After an individual has a first stroke, he or she is at increased risk for a subsequent stroke (Figure 13). The progression function for recurrent stroke depends on age, sex, race, and the age at which the first stroke occurred. The type of stroke (ischemic or hemorrhagic) depends on the type of the first stroke; there is an increased probability that a recurrent stroke will be the same type as the first.

The progression functions for recurrent strokes are based on:

- Lloyd-Jones D et al. Heart disease and stroke statistics—2010 update: a report from the American Heart Association, 2010; 121:e46-e215.
- Hankey GJ et al. Five-year survival after first-ever stroke and related prognostic factors in the Perth Community Stroke Study, Stroke 2000; 31:2080-2086.
- Hardie K, et al. Ten-year risk of first recurrent stroke and disability after first-ever stroke in the Perth Community Stroke Study, Stroke 2004; 35:731-735.
- Hillen T et al. Cause of stroke recurrence is multifactorial: patterns, risk factors, and outcomes of stroke recurrence in the South London stroke register, Stroke 2003; 34:1457-1463.

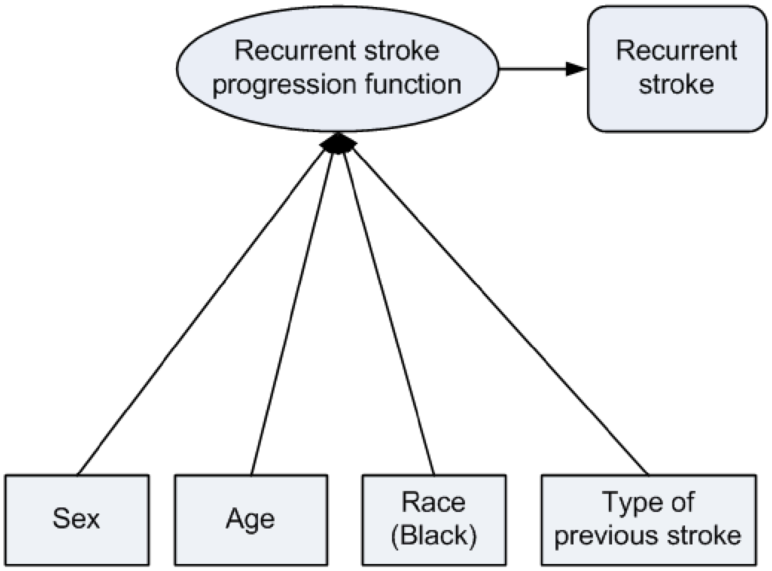

Figure 13. Diagram of Recurrent Stroke Model.

Chronic Obstructive Pulmonary Disease (COPD) Model

A new model of chronic obstructive pulmonary disease (COPD) was added in Simulator version 2.5. Risk factors for COPD are age, sex, race, and smoking history and status. Risk of MI and stroke are both increased by the presence of COPD.

© 2011-2013

The COPD model is based on two progression functions that correspond to two clinical measures used to evaluate pulmonary function: FEV_1, forced expiratory volume in one second, and FVC, forced vital capacity.

The COPD model is based on:

- Anthonisen NR et al. Smoking and lung function of lung health study participants after 11 years. American Journal of Respiratory and Critical Care Medicine 2002; 166(5):675–679.
- Calverley P & Anderson J. Salmeterol and fluticasone propionate and survival in chronic obstructive pulmonary disease. N Eng J Med 2007; 356:775-789.
- Drummond MB et al. Spirometric predictors of lung function decline and mortality in early chronic obstructive pulmonary disease. American Journal of Respiratory and Critical Care Medicine 2012; 185(12):1301–1306.
- Fletcher C & Peto R. The natural history of chronic airflow obstruction. BMJ 1977; 1(6077): 1645–1648.
- Garcia-Aymerich J et al. Risk factors of readmission to hospital for a COPD exacerbation: a prospective study. Thorax 2003; 58:100-105.
- Garcia-Aymerich, J et al. Regular physical activity reduces hospital admission and mortality in chronic obstructive pulmonary disease: a population based cohort study. Thorax 2006; 61:772–778.
- Hankinson JL et al. Spirometric reference values from a sample of the general U.S. population. American Journal of Respiratory and Critical Care Medicine 1999; 159(1):179–187.
- Jansson S et al. Costs of COPD in Sweden according to disease severity. Chest 2002; 122(6):1994-2002.
- Miravitlles M et al. Costs of chronic bronchitis and COPD: a 1-year follow-up study. Chest 2003; 123(3):784–791.
- Niewoehner DE et al. (2007). Risk indexes for exacerbations and hospitalizations due to COPD. Chest 2007; 131(1):20–28.
- Niewoehner DE et al. Prevention of exacerbations of chronic obstructive pulmonary disease with tiotropium, a once-daily inhaled anticholinergic bronchodilator: a randomized trial. Annals of Internal Medicine 2005; 143(5):317-326.

© 2011-2013

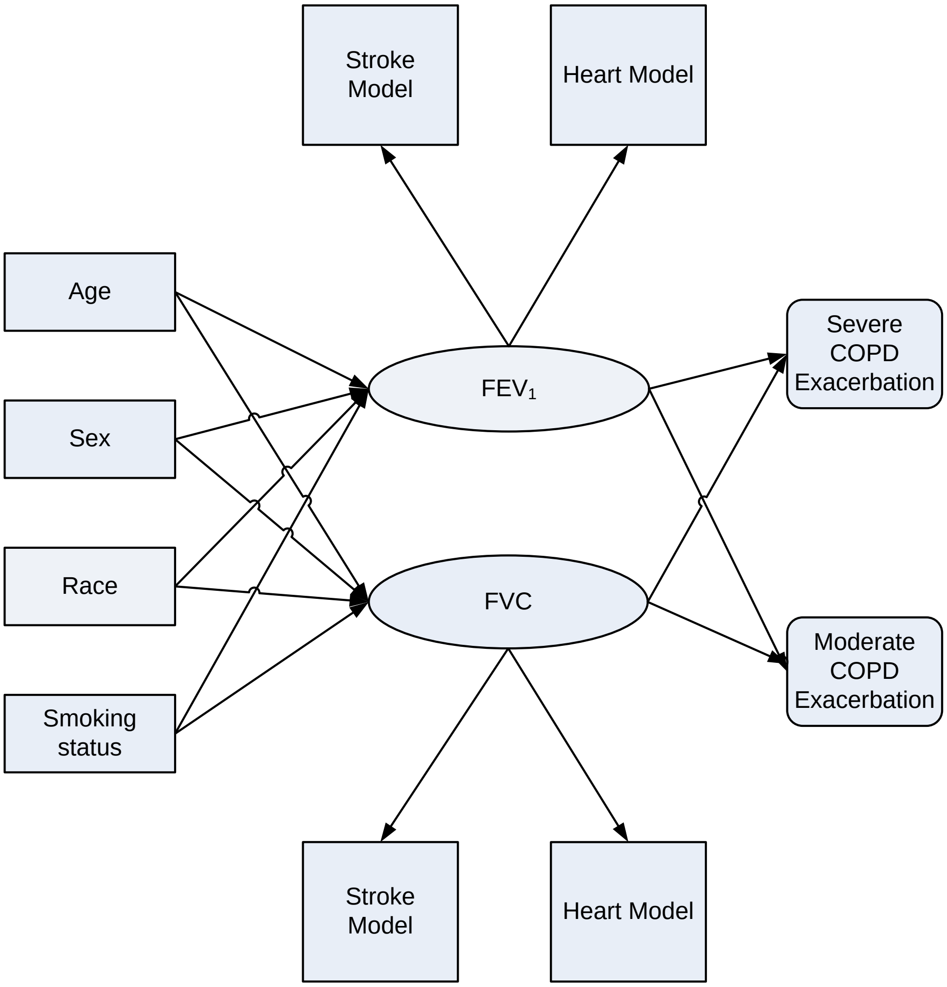

Figure 14. Diagram of COPD Model.

Mortality

The Archimedes Model includes a model for calculating all-cause mortality based on data from the National Vital Statistics System (NVSS). In the Model, disease-specific mortalities are generated by the individual disease models. The Model then adjusts the all-cause mortality rate to determine a progression function for “other-cause” mortality that includes deaths from all causes other than the diseases explicitly included in the Archimedes Model.

The mortality models are based on the following publications from the National Center for Health Statistics:

- Arias E. United States Life Tables, 2006. National Vital Statistics Reports; vol. 58 no 21. Hyattsville, MD: National Center for Health Statistics. 2010.

- Arias E. United States Life Tables by Hispanic Origin. National Center for Health Statistics. Vital Health Stat 2010; 2(152).
- Death rates from 113 selected causes, specified Hispanic origin, race for non-Hispanic population, United States, 2006. (Available at http://www.cdc.gov/nchs/nvss/mortality/gmwkh210r.htm.)

Interventions

The Archimedes Model includes a large number of tests and treatments. Many but not all of them are accessible through the ARCHeS interface. This section describes the interventions that are accessible through ARCHeS. We use the term "intervention" very broadly to include not only drugs and procedures, but also anything that directly or indirectly affects either a risk factor for a disease or the progression and outcome of a disease. Thus interventions can include behavior changes such as diet and exercise, and public health measures such as reducing salt or substituting trans fats in foods. Interventions can also include activities whose effects on risk factors are very indirect. For example a media-based program to increase the rate at which patients adhere to recommended treatments is an intervention and can be evaluated using the Archimedes Model and ARCHeS. Even a policy initiative such as increasing insurance coverage can be addressed if the changes in coverage affect risk factors and diseases that are in the Model. For interventions whose effects on risk factors and diseases are indirect, it is necessary to have evidence about the effect of the intervention on risk factors or diseases. For example, to analyze the effects on health and economic outcomes of a media-based program to increase patient adherence to recommendations for hypertension treatment, it is necessary to have evidence from a trial or demonstration program showing the effect of the program on use of blood pressure medications, or preferably on actual blood pressure levels.

In the Model, interventions can affect biomarkers directly and can have additional "pleiotropic" effects on the progression of diseases (the disease progression functions). Pleiotropic effects of an intervention are effects on the risk of developing a disease, or of the disease progressing, that go beyond the known effects of an intervention on specific biomarkers.

Table 4 shows the biomarkers and disease processes affected by each intervention in the part of the Model that is included in ARCHeS. Note that the biomarker changes that result from an intervention may themselves affect other biomarkers and disease progressions due to linkages within the Model.

Table 4. Biomarkers and Disease Processes Affected by Each Intervention.

Intervention	Effects
Aspirin	Stable angina, MI, ischemic stroke
Diuretic	Stable angina, MI, blood pressure, ischemic stroke, hemorrhagic stroke, CHD death, heart failure onset
ACE inhibitor/ARB	Stable angina, MI, blood pressure, ischemic stroke, hemorrhagic stroke, CHD death, heart failure onset, renal disease
Beta blocker	Stable angina, MI, blood pressure, ischemic stroke, hemorrhagic stroke, CHD

© 2011-2013

	death, heart failure onset
Calcium channel blocker	Stable angina, MI, blood pressure, ischemic stroke, hemorrhagic stroke, CHD death, heart failure onset
Metformin	Weight, FPG, total cholesterol, HDL, TGs, systolic blood pressure
Sulfonylurea	Weight, insulin amount, FPG, possibility of hypoglycemia
Glitazones	Weight, FPG, total cholesterol, HDL, TGs
Insulin	Weight, insulin amount, FPG, possibility of hypoglycemia
Statin	Total cholesterol, HDL, TG, MI, ischemic stroke, heart failure onset, CHD death
Diabetes diet	Weight, blood pressure

Aspirin Model

In the Model, aspirin has both primary prevention effects (changes to the risk of a first event) and secondary prevention effects (changes to the risk of a repeat event in people who have already had a first event).

The dose of aspirin used in the model is 81mg/day. This is the dose most commonly prescribed in the US.

References for the Aspirin Intervention Model

1. Berger JS et al. Aspirin for the primary prevention of cardiovascular events in women and men: a sex-specific meta-analysis or randomized controlled trials, JAMA 2006; 295(3):306-313.
2. Antithrombotic Trialists' (ATT) Collaboration. Aspirin in the primary and secondary prevention of vascular disease: collaborative meta-analysis of individual participant data from randomised trials, Lancet 2009; 373(9678):1849-1860.

Summary of the Aspirin Intervention Model

The aspirin model is primarily based on the meta-analysis in (1). This study is consistent with (2).

Aspirin treatment does not affect individual biomarkers. Instead, aspirin treatment is modeled as a multiplier of the MI and stroke hazard rate functions. The effects of aspirin differ in men and women.

Table 5 shows the diseases and outcomes in the Model that are affected when a patient takes aspirin, and the multiplier used. For example, when a woman in the Model starts taking aspirin, the ischemic stroke hazard rate is multiplied by 0.76.

© 2011-2013

Table 5. Diseases and Outcomes Affected by the Aspirin Intervention.

Condition/outcome	Multiplier (men)	Multiplier (women)	References
Ischemic stroke	1.0	0.76	1, 2
First myocardial infarction (MI)	0.68	1	1
Recurrent MI	0.8	0.8	2

Statin Model

In the Model, statins affect total cholesterol, HDL, and triglycerides, and also have pleiotropic effects on myocardial infarction and ischemic stroke rates. Note that LDL is indirectly affected via the Friedewald equation.

References for the Statin Intervention Model

1. Jones PH et al. Comparison of the efficacy and safety of rosuvastatin versus atorvastatin, simvastatin, and pravastatin across doses (STELLAR* Trial), Am J Cardiol 2003; 92(2):152-160.
2. The ALLHAT Officers and Coordinators for the ALLHAT Collaborative Research Group. Major outcomes in moderately hypercholesterolemic, hypertensive patients randomized to pravastatin vs usual care: the antihypertensive and lipid-lowering treatment to prevent heart attack trial (ALLHAT-LLT), JAMA 2002; 288(23):2998-3007.
3. Sever PS et al. Prevention of coronary and stroke events with atorvastatin in hypertensive patients who have average or lower-than-average cholesterol concentrations, in the Anglo-Scandinavian Cardiac Outcomes Trial – Lipid Lowering Arm (ASCOT-LLA): a multicentre randomised controlled trial, Lancet 2003; 361:1149-1158.
4. Colhoun HM et al. Primary prevention of cardiovascular disease with atorvastatin in type 2 diabetes in the Collaborative Atorvastatin Diabetes Study (CARDS): multicentre randomised placebo-controlled trial, Lancet 2004; 364:685-696.
5. Sacks FM et al. The effect of pravastatin on coronary events after myocardial infarction in patients with average cholesterol levels, NEJM 1996; 335(14):1001-1009.
6. Pedersen TR et al. High-dose atorvastatin vs usual-dose simvastatin for secondary prevention after myocardial infarction: the IDEAL study: a randomized controlled trial, JAMA 2005; 294(19): 2437-2445.
7. LaRosa JC et al. Intensive lipid lowering with atorvastatin in patients with stable coronary disease, NEJM 2005; 352(14):1425-1435.
8. Scirica BM et al. Intensive statin therapy and the risk of hospitalization for heart failure after an acute coronary syndrome in the PROVE IT-TIMI 22 study, J Amer Coll Cardiol 2006; 47(11):2326-2331.
9. Sheperd J et al. Effect of lowering LDL cholesterol substantially below currently recommended levels in patients with coronary heart disease and diabetes: the treating to new targets (TNT) study, Diabetes Care 2006; 29(6):1220-1226.

ARCHIMEDES
© 2011-2013

10. Scirica BM et al. Intensive statin therapy and the risk of hospitalization for heart failure after an acute coronary syndrome in the PROVE IT–TIMI 22 study, Journal of the American College of Cardiology 2006; 47:2326-2331.

Summary of the Statin Intervention Model

The effect of different statins on the biomarkers shown in Table 6 is based on the results of the STELLAR trial (1). The overall effect of statins on MI and ischemic stroke rates is based on a meta-analysis performed by Archimedes using data from references (2), (3), (4), (5), (6), (7), and (9). The effect of statins on heart failure onset is based on a meta-analysis from reference (10).

Table 6 shows the biomarkers and outcomes in the Model that are affected when a patient takes a statin.

Table 6. Effects of the Statin Intervention on Biomarkers and Outcomes.

Statin	TC	HDL	TG	MI	Ischemic Stroke	Heart Failure Onset	CHD Mortality
Simvastatin 5mg	0.83	1.05	0.88	0.75	0.75	0.78	0.75
Simvastatin 10mg	0.79	1.05	0.87	0.71	0.69	0.74	0.71
Simvastatin 20mg	0.75	1.06	0.85	0.64	0.60	0.68	0.64
Simvastatin 40mg	0.71	1.06	0.84	0.63	0.58	0.66	0.63
Simvastatin 80mg	0.67	1.06	0.82	0.57	0.49	0.61	0.57
Atorvastatin 80mg	0.61	1.02	0.72	0.46	0.34	0.49	0.46
**TC**: total cholesterol, **HDL**: high-density lipoprotein, **TG**: triglycerides, **MI**: myocardial infarction, **CHD:** coronary heart disease							

Diabetes Medications

The following diabetes medications are included in the Model: metformin, sulfonylurea, insulin, and glitazones.

Metformin Model

In the Model, metformin affects fasting plasma glucose, weight, systolic blood pressure, total cholesterol, HDL, and triglycerides.

References for the Metformin Intervention Model

1. Amador-Licona N et al. The short-term effect of a switch from glibenclamide to metformin on blood pressure and microalbuminuria in patients with type 2 diabetes mellitus. Archives of Medical Research 2000; 31(6):571–575.
2. Boyd K et al. Insulin, glibenclamide or metformin treatment for non insulin dependent diabetes: heterogenous responses of standard measures of insulin action and insulin secretion before and after differing hypoglycaemic therapy. Diabetes Research 1992; 19(2):69–76.
3. Campbell IW et al. One year comparative trial of metformin and glipizide in Type 2 diabetes mellitus. Diabete et Metabolisme 1994; 20:394–400.

4. Charpentier G et al. Improved glycaemic control by addition of glimepiride to metformin monotherapy in type 2 diabetic patients. Diabetes Medicine 2001; 18(10):828–834.
5. Collier A et al. Effect of glycaemic control, metformin and gliclazideon platelet density and aggregability in recently diagnosed type 2 (non-insulin-dependent) diabetic patients. Diabete etMetabolisme 1989; 15(6):420–425.
6. Dalzell GW et al. A randomized trial tolbutamideand metformin for persistent severe hyperglycaemia innon insulin dependent diabetes mellitus (NIDDM). IrishJournal of Medical Science 1986; 155(9):341–342.
7. Damsbo P et al. Irreversibility of the defect in glycogensynthase activity in skeletal muscle from obese patients withNIDDM treated with diet and metformin. Diabetes Care1998; 21(9):1489–1494.
8. DeFronzo RA et al. Efficacy of metformin in patients with non-insulin-dependent diabetes mellitus. The Multicenter Metformin Study Group [see comments]. New England Journal of Medicine 1995; 333(9):541–549.
9. Del Prato S et al. Six-month efficacy of benfluorex vs. placebo or metformin in dietfailed type 2 diabetic patients. Acta Diabetologica 2003; 40 (1):20–27.
10. Dornan TL et al. Doubleblind evaluation of efficacy and tolerability of metformin in NIDDM. Diabetes Care 1991; 14(4):342–344.
11. Fanghanel G et al. Metformin's effects on glucose and lipid metabolism in patients with secondary failure to sulfonylureas. Diabetes Care 1996; 19(11):1185–1189.
12. Goldstein BJ et al. Multicenter, randomized, double-masked, parallel-group assessment of simultaneous glipizide /metformin as second-line pharmacologic treatment for patients with type 2 diabetes mellitus that is inadequately controlled by a sulfonylurea. Clinical Therapeutics 2003; 25(3):890–903.
13. Grant PJ. The effects of high- and medium-dose metformin therapy on cardiovascular risk factors in patients with type II diabetes. Diabetes Care 1996; 19(1):64–66.
14. Hallsten K et al.Rosiglitazone but not metformin enhances insulin- and exercise-stimulated skeletal muscle glucose uptake in patients with newly diagnosed type 2 diabetes. Diabetes 2002; 51(12):3479–3485.
15. Hermann LS et al. Prospective comparative study in NIDDM patients of metformin and glibenclamide with special reference to lipid profiles. Eur J of Clin Pharm 1991; 41(3):263-265.
16. Hoffmann J et al. Efficacy of 24-week monotherapy with acarbose, metformin, or placebo in dietary-treated NIDDM patients: the Essen-II Study. American Journal of Medicine 1997; 103(6):483–490.
17. Horton ES et al. Nateglinide alone and in combination with metformin improves glycemic control by reducing mealtime glucose levels in type 2 diabetes. Diabetes Care 2000; 23 (11):1660–1665.
18. Inzucchi SE et al.Efficacy and metabolic effects of metformin and troglitazone in type II diabetes mellitus [see comments]. New England Journal of Medicine 1998; 338(13):867–872.

© 2011-2013

19. Johnson AB et al.The impact of metformin therapy on hepatic glucose production and skeletal muscle glycogen synthase activity in overweight type II diabetic patients. Metabolism: Clinical and Experimental 1993; 42(9): 1217–1222.
20. Josephkutty S et al. Comparison of tolbutamide and metformin in elderly diabetic patients. Diabetic Medicine 1990; 7:510–514.
21. Lalor BC et al.Placebo-controlled trial of the effects of guar gum and metformin on fasting blood glucose and serum lipids in obese, type 2 diabetic patients. Diabetic Medicine 1990; 7:242–245.
22. Lee A et al. Metformin decreases food consumption and induces weight loss in subjects with obesity with type II non-insulin-dependent diabetes. Obesity Research 1998; 6 (1):47–53.
23. Mather KJ et al. Improved endothelial function with metformin in type 2 diabetes mellitus. Journal of the American College of Cardiology 2001; 37(5):1344–1350.
24. Moses R et al.Effect of repaglinide addition to metformin monotherapy on glycemic control in patients with type 2 diabetes. Diabetes Care 1999; 22(1):119–124.
25. Nagi DK et al. Effects of metformin on insulin resistance, risk factors for cardiovascular disease, and plasminogen activator inhibitor in NIDDM subjects. A study of two ethnic groups. Diabetes Care 1993; 16(4): 621–629.
26. Noury J et al. Comparative three-month study of the efficacies of metformin and gliclazide in the treatment of NIDD. Diabete et Metabolisme 1991; 17(1 Pt 2):209–212.
27. Rains SG et al. The effect of glibenclamide and metformin on serum lipoproteins in type 2 diabetes. Diabetic Medicine 1988; 5 (7):653–658.
28. Tamez Pérez HE et al. Normoglucemia in newly diagnosed no insulin dependent diabetes mellitus. Non pharmacologic therapy vs. pharmacologic therapy [Normoglucemia en diabetes mellitus no dependiente de insulina de reciente diagnóstico. Tratamiento no farmacológico vs. tratamiento farmacológico]. Medicina Interna de Mexico 1997; 13(6): 272–275.
29. Tessari P et al.Effects of metformin treatment on whole-body and splanchnic amino acid turnover in mild type 2 diabetes. Journal of Clinical Endocrinology and Metabolism 1994; 79:1553–1560.
30. Tessier D et al. Effects of gliclazide versus metformin on the clinical profile and lipid peroxidation markers in type 2 diabetes. Metabolism: Clinical and Experimental 1999; 48(7):897–903.
31. Teupe B et al. Prospective randomized two-years clinical study comparing additional metformin treatment with reducing diet in type 2 diabetes. Diabete et Metabolisme 1991; 17:213–217.
32. Turner RC et al. Effect of intensive blood-glucose control with metformin on complications in overweight patients with type 2 diabetes (UKPDS 34). Lancet 1998; 352(9131):854–65.
33. Uehara MH. Metabolic and hemodinanic effects of metformin in patients with type 2 diabetes mellitus and hypertension [Efeitos metabólicos e hemodinâmicos da metformina em pacientes com diabetes mellitus tipo 2 e hipertensäo arterial essencial]. Tesis: Universidae Federal de Säo Paulo. Escola Paulista de Medicina para obtención del grado de Doutor 1999.

© 2011-2013

Summary of the Metformin Intervention Model

Table 7 shows the biomarkers in the Model that are affected when a patient takes metformin, and the multiplier used. These effects are based on a meta-analysis performed by Archimedes based on data from the studies listed in the References section above.

Table 7. Biomarkers Affected by the Metformin Intervention.

Biomarker	Effect or Multiplier
Fasting plasma glucose	Post-treatment value = 31.88 + 0.6647 * pre-treatment FPG
Weight	0.977
Total cholesterol	0.963
HDL	1.089
Triglycerides	0.898
Systolic blood pressure	0.972

Sulfonylurea Model

In the Model, sulfonylurea affects HbA1c and weight.

References for the Sulfonylurea Intervention Model

1. Ferrannini E et al. Fifty-two-week efficacy and safety of vildagliptin vs. glimepiride in patients with type 2 diabetes mellitus inadequately controlled on metformin monotherapy. Diabetes Obes Metab 2009; 11:157-66.
2. Nauck MA et al. Dapagliflozin versus glipizide as add-on therapy in patients with type 2 diabetes who have inadequate glycemic control with metformin: a randomized, 52-week, double-blind, active-controlled noninferiority trial. Diabetes Care 2011; 34:2015-22.
3. Goke B et al. Saxagliptin is non-inferior to glipizide in patients with type 2 diabetes mellitus inadequately controlled on metformin alone: a 52-week randomised controlled trial. Int J Clin Pract 2010; 64:1619-31.
4. Filozof C et al. A comparison of efficacy and safety of vildagliptin and gliclazide in combination with metformin in patients with Type 2 diabetes inadequately controlled with metformin alone: a 52-week, randomized study. Diabet Med 2010; 27:318-26.
5. Nauck MA et al. Efficacy and safety of the dipeptidyl peptidase-4 inhibitor, sitagliptin, compared with the sulfonylurea, glipizide, in patients with type 2 diabetes inadequately controlled on metformin alone: a randomized, double-blind, non-inferiority trial. Diabetes Obes Metab 2007; 9:194-205.
6. Arechavaleta R et al. Efficacy and safety of treatment with sitagliptin or glimepiride in patients with type 2 diabetes inadequately controlled on metformin monotherapy: a randomized, double-blind, non-inferiority trial. Diabetes Obes Metab 2011; 13:160-8.
7. Nauck M et al. Efficacy and safety comparison of liraglutide, glimepiride, and placebo, all in combination with metformin, in type 2 diabetes: the LEAD (liraglutide effect and action in diabetes)-2 study. Diabetes Care 2009; 32:84-90.

© 2011-2013

8. Hamann A et al. Comparison of fixed-dose rosiglitazone/metformin combination therapy with sulphonylurea plus metformin in overweight individuals with Type 2 diabetes inadequately controlled on metformin alone. Exp Clin Endocrinol Diabetes 2008; 116:6-13.
9. Khanolkar MP et al. Rosiglitazone produces a greater reduction in circulating platelet activity compared with gliclazide in patients with type 2 diabetes mellitus--an effect probably mediated by direct platelet PPARgamma activation. Atherosclerosis 2008; 197:718-24.
10. Ristic S et al. Nateglinide or gliclazide in combination with metformin for treatment of patients with type 2 diabetes mellitus inadequately controlled on maximum doses of metformin alone: 1-year trial results. Diabetes Obes Metab 2007; 9:506-11.
11. Feinglos M et al. Effect on glycemic control of the addition of 2.5 mg glipizide GITS to metformin in patients with T2DM. Diabetes Res Clin Pract 2005; 68:167-75.
12. Matthews DR et al. Long-term therapy with addition of pioglitazone to metformin compared with the addition of gliclazide to metformin in patients with type 2 diabetes: a randomized, comparative study. Diabetes Metab Res Rev 2005; 21:167-74.
13. Charpentier G et al. Improved glycaemic control by addition of glimepiride to metformin monotherapy in type 2 diabetic patients. Diabet Med 2001; 18:828-34.
14. Phung OJ et al. Effect of noninsulin antidiabetic drugs added to metformin therapy on glycemic control, weight gain, and hypoglycemia in type 2 diabetes. JAMA 2010; 303:1410-1418.

Summary of the Sulfonylurea Intervention Model

Table 8 shows the biomarkers in the Model that are affected when a patient takes sulfonyurea. These effects are based on a meta-analysis performed by Archimedes based on data from the studies listed in the References section above.

Table 8. Biomarkers Affected by the Sulfonylurea Intervention.

Biomarker	Effect or Multiplier	References
HbA1c	Post-treatment value =<br>2.1 + 0.64 * pre-treatment HbA1c	1 – 13
Weight	1.023	14

Insulin Model

Only insulin glargine is modeled. In the Model, insulin affects HbA1c and weight.

References for the Insulin Intervention Model

1. Al-Shaikh AR. Comparison of Basal Insulin Added to Oral Agents Versus Twice-Daily Premixed Insulin as Initial Insulin Therapy for Type 2 Diabetes. Pak J Med Sci 2006; 22:14-17.
2. Barnett A. Dosing of insulin glargine in the treatment of type 2 diabetes. Clin Ther. 2007; 29:987-999.
3. Bretzel RG et al. Initiating insulin therapy in type 2 diabetic patients failing on oral hypoglycemic agents: basal or prandial insulin? The APOLLO trial and beyond. Diabetes Care 2009; 32 Suppl 2:S260-265.

4. Fonseca V et al. An analysis of early insulin glargine added to metformin with or without sulfonylurea: impact on glycaemic control and hypoglycaemia. Diabetes Obes Metab 2011; 13:814-822.
5. Heise T et al. Glycemic control with preprandial versus basal insulin in patients with type 2 diabetes mellitus poorly controlled by oral antidiabetes agents. Diabetes Technol Ther 2010; 12:135-141.
6. Hollander P et al. A 52-week, multinational, open-label, parallel-group, noninferiority, treat-to-target trial comparing insulin detemir with insulin glargine in a basal-bolus regimen with mealtime insulin aspart in patients with type 2 diabetes. Clin Ther 2008; 30(11):1976-1987.
7. Home PD et al. Meta-analysis of individual patient data to assess the risk of hypoglycaemia in people with type 2 diabetes using NPH insulin or insulin glargine. Diabetes Obes Metab 2010; 12:772-779.
8. Kennedy L et al. Impact of active versus usual algorithmic titration of basal insulin and point-of-care versus laboratory measurement of HbA1c on glycemic control in patients with type 2 diabetes: the Glycemic Optimization with Algorithms and Labs at Point of Care (GOAL A1C) trial. Diabetes Care 2006; 29:1-8.
9. Lee LJ et al. Effects of insulin initiation on patient-reported outcomes in patients with type 2 diabetes: results from the durable trial. Diabetes Res Clin Pract 2010; 89:157-166.
10. Meneghini LF et al. Improved glycemic control with insulin glargine versus pioglitazone as add-on therapy to sulfonylurea or metformin in patients with uncontrolled type 2 diabetes mellitus. Endocr Pract. 2010; 16:588-599.
11. Pollock RF et al. Long-acting insulin analogs: a review of "real-world" effectiveness in patients with type 2 diabetes. Curr Diabetes Rev. 2011; 7:61-74.
12. Raskin P et al. Comparison of insulin detemir and insulin glargine using a basal-bolus regimen in a randomized, controlled clinical study in patients with type 2 diabetes. Diabetes Metab Res Rev 2009; 25(6): 542-548.
13. Rosenstock J et al. A randomised, 52-week, treat-to-target trial comparing insulin detemir with insulin glargine when administered as add-on to glucose-lowering drugs in insulin-naive people with type 2 diabetes. Diabetologia 2008; 51(3): 408-416.
14. Schreiber SA et al. The long-term efficacy of insulin glargine plus oral antidiabetic agents in a 32-month observational study of everyday clinical practice. Diabetes Technol Ther. 2008; 10:121-127.
15. Swinnen SG et al. A 24-week, randomized, treat-to-target trial comparing initiation of insulin glargine once-daily with insulin detemir twice-daily in patients with type 2 diabetes inadequately controlled on oral glucose-lowering drugs. Diabetes Care 2010; 33(6):1176-1178.
16. Triplitt C et al. Comparison of glargine insulin versus rosiglitazone addition in poorly controlled type 2 diabetic patients on metformin plus sulfonylurea. Diabetes Care. 2006; 29:2371-2377.
17. Tsai ST et al. First insulinization with basal insulin in patients with Type 2 diabetes in a real-world setting in Asia. J Diabetes. 2011; 3:208-216.

18. UK Prospective Diabetes Study (UKPDS) Group. Intensive blood-glucose control with sulphonylureas or insulin compared with conventional treatment and risk of complications in patients with type 2 diabetes (UKPDS 33). Lancet. 1998; 352:837-853.
19. Wang F et al. Insulin glargine: a systematic review of a long-acting insulin analogue. Clin Ther. 2003; 25:1541-1577.

Summary of the Insulin Intervention Model

Table 9 shows the biomarkers in the Model that are affected when a patient takes insulin. These effects are based on a meta-analysis performed by Archimedes based on data from the studies listed in the References section above.

Table 9. Biomarkers Affected by the Insulin Intervention.

Biomarker	Effect or Multiplier	References
HbA1c	Post-treatment value =<br>2.2 + 0.6 * pre-treatment HbA1c	1 ,2, 3, 4, 5, 7, 8, 9, 10, 11,<br>14, 16, 17, 18, 19
Weight	1.028	6, 12, 13, 15

Glitazone Model

The Model includes a glitazone intervention that was built from data on pioglitazones. It affects fasting plasma glucose, weight, total cholesterol, HDL, and triglycerides.

References for the Glitazone Intervention Model

1. Goldberg RB et al (for GLAI). A comparison of lipid and glycemic effects of pioglitazone and rosiglitazone in patients with type 2 diabetes and dyslipidemia, Diabetes Care 2005; 28(7):1547-1554.
2. Tan MH et al (for GLAL). Comparison of pioglitazone and gliclazide in sustaining glycemic control over 2 years in patients with type 2 diabetes, Diabetes Care 2005; 28(3):544-550.
3. Chiquette E et al. A meta-analysis comparing the effect of thiazolidinediones on cardiovascular risk factors, Arch Intern Med 2004; 164:2097-2104.

Summary of the Glitazone Intervention Model

Table 10 shows the biomarkers in the Model that are affected when a patient takes a glitazone. These effects are based on a meta-analysis performed by Archimedes based on data from the studies listed in the References section above.

© 2011-2013

Table 10. Biomarkers Affected by the Glitazone Intervention.

Biomarker	Multiplier	References
Fasting plasma glucose	0.81	1, 2, 3
Weight	1.033	1, 2, 3
Total cholesterol	1.05	1, 3
HDL	1.13	1, 3
Triglycerides	0.81	1, 3

GLP-1 Agonist and DPP-4 Inhibitor Models

The Model includes GLP-1 agonist and DPP-4 inhibitor interventions that were built from data sources included in two meta-analyses.

References for the GLP-1 Agonist and DPP-4 Inhibitor Intervention Models

1. Esposito K, Chiodini P, Bellastella G, Maiorino MI, Giugliano D. Proportion of patients at HbA1c target < 7% with eight classes of antidiabetic drugs in type 2 diabetes: systematic review of 218 randomized controlled trials with 78945 patients. Diabetes, Obesity and Metabolism (DOM) [Systematic review]. 2011 2011;14:228-33.
2. Aroda VR, Henry RR, Han J, Huang W, DeYoung MB, Darsow T, et al. Efficacy of GLP-1 Receptor Agonists and DPP-4 Inhibitors: Meta-Analysis and Systematic Review. Clinical Therapeutics. 2012.

Summary of the GLP-1 Agonist Model

Table 11 shows the biomarkers in the Model that are affected when a patient takes a GLP-1 agonist. These effects are based on a meta-analysis performed by Archimedes based on data from the studies listed in the References section above.

Table 11. Biomarkers Affected by the GLP-1 Agonist.

Biomarker	Multiplier	References
HbA1c	0.860	1, 2
Weight	0.978	1, 2
SBP	0.979	1, 2

Summary of the DPP-4 Inhibitor Model

The DPP-4 inhibitor intervention affects only HbA1c in the Model. Its effect is based on a meta-analysis performed by Archimedes. The multiplier on HbA1c is 0.909.

Blood Pressure Medications Model

References for the Blood Pressure Medications Model

1. Sciarretta et al. Antihypertensive Treatment and Development of Heart Failure in Hypertension, Arch Intern Med 2011; 171(5): 384-394.

2. Law MR et al. Use of blood pressure lowering drugs in the prevention of cardiovascular disease: meta-analysis of 147 randomised trials in the context of expectations from prospective epidemiological studies, BMJ 2009; 338:b1665.
3. Amery A et al. Mortality and morbidity from the European Working Party on High Blood Pressure in the Elderly trial, Lancet 1985; 1:1349-1354.
4. Beckett NS et al. Treatment of hypertension in patients 80 years of age or older, NEJM 2008; 358(18):1887-1898.
5. Black HR et al. Controlled Onset Verapamil Investigation of Cardiovascular Endpoints: CONVINCE primary results. Presented at: American Society of Hypertension; May 18, 2002, New York, NY.
6. Coope J et al. Randomised trial of treatment of hypertension in elderly patients in primary care, BMJ 1986; 293(6555):1145-1151.
7. Dahlof B et al. Morbidity and mortality in the Swedish Trial in Old Patients with Hypertension (STOP-Hypertension), Lancet 1991; 338:1281-1285.
8. The Dutch TIA Trial Study Group. Trial of secondary prevention with atenolol after transient ischemic attack or nondisabling ischemic stroke, Stroke 1993; 24:543-548.
9. Eriksson S et al for the TEST study group. Atenolol in secondary prevention after stroke, Cerebrovasc Dis 1995; 5:21-25.
10. Hansson L et al. Randomised trial of old and new antihypertensive drugs in elderly patients: cardiovascular mortality and morbidity the Swedish Trial in Old Patients with Hypertension-2 study, Lancet 1999; 354:1751-1756.
11. The IPPPSH Collaborative Group. Cardiovascular risk and risk factors in a randomized trial of treatment based on the beta-blocker oxprenolol: the international prospective primary prevention study in hypertension (IPPPSH), Journal of Hypertension 1985; 3:379-392.
12. Mancia G et al. Clinical Trial-INVEST Blood pressure control and improved cardiovascular outcomes in the international verapamil SR-trandolapril study, Hypertension 2007; 50:299-305.
13. Medical Research Council Working Party. MRC trial of treatment of mild hypertension: principal results, BMJ 1985; 291:97-104.
14. Passa P et al. The DIAB-HYCAR Study, Diabetologia 1996; 39(12):1662-1667.
15. PROGRESS Collaborative Group. Randomised trial of perindopril-based blood pressure-lowering regimen among 6105 individuals with previous stroke or transient ischaemic attack, Lancet 2001; 358:1033-1041.
16. Psaty BM et al. Health outcomes associated with various antihypertensive therapies used as first-line agents: a network meta-analysis, JAMA 2003; 289(19):2534-2544.
17. Sever PS et al. Prevention of coronary and stroke events with atorvastatin in hypertensive patients who have average or lower-than-average cholesterol concentrations, in the Anglo-Scandinavian Cardiac Outcomes Trial—Lipid Lowering Arm (ASCOT-LLA): a multicentre randomised controlled trial, Lancet 2003; 361:1149.
18. SHEP Cooperative Research Group. Prevention of stroke by antihypertensive drug treatment in older persons with isolated systolic hypertension: final results of the systolic hypertension in the elderly program (SHEP), JAMA 1991; 265(24):3255-3264.

© 2011-2013

19. Wang JG et al. Prevention of stroke and myocardial infarction by amlopidine and angiotensin receptor blockers: a quantitative overview, Hypertension 2007; 50:181-188.
20. Wing LMH et al for the Second Australian National Blood Pressure Study Group. A comparison of outcomes with angiotensin-converting-enzyme inhibitors and diuretics for hypertension in the elderly, NEJM 2003; 348:583-592.
21. Zanchetti Z et al. Calcium antagonist lacidipine slows down progression of asymptomatic carotid atherosclerosis: principal results of the European Lacidipine Study on Atherosclerosis (ELSA): a randomized, double-blind, long-term trial, Circulation 2002; 106:2422-2427.

Summary of the Blood Pressure Medications Model

The effect of different types of blood pressure medications on the biomarkers shown in Table 12 is based on a meta-analysis performed by Archimedes using data from the references listed above.

The overall effect of antihypertensive treatment on the development of congestive heart failure (CHF) was based on (1), and the overall effect on recurrent myocardial infarctions was based on (2).

Table 12 shows the biomarkers and outcomes in the Model that are affected when a patient takes one of the classes of blood pressure medications included in the Model.

Table 12. Effects of Hypertensive Medications on Biomarkers and Outcomes.

Anti-Hypertensive	SBP/DBP	Myocardial Infarction	Ischemic or Hemorrhagic Stroke	CHD Mortality	Heart Failure Onset
ACE inhibitor	0.94	0.68	0.82	0.68	0.75
Calcium channel blocker	0.93	0.81	0.71	0.81	0.86
Diuretic	0.91	0.65	0.67	0.65	0.64
Beta blocker	0.95	0.91	0.81	0.71	0.92

Diabetes Diet Model

References for the Diabetes Diet Model

1. UKPDS Group. UK Prospective Diabetes Study 7: response of fasting plasma glucose to diet therapy in newly presenting type II diabetic patients, Metabolism 1990; 39(9):905-912.
2. UKPDS Group. Effect of three months' diet after diagnosis of type 2 diabetes on plasma lipids and lipoproteins (UKPDS 45), Diabet Med 2000; 17:518-523.
3. UK Prospective Diabetes Study (UKPDS) Group. Intensive blood-glucose control with sulphonylureas or insulin compared with conventional treatment and risk of complications in patients with type 2 diabetes (UKPDS 33), Lancet 1998; 352(9131):837-853.
4. Neter JE et al. Influence of weight reduction on blood pressure: a meta-analysis of randomized controlled trials, Hypertension 2003; 42:878-884.

© 2011-2013

Summary of the Diabetes Diet Model

The diabetes diet intervention produces a 3% weight loss (and thus a decrease in 3% BMI), based on references (1), (2), and (3) above. Weight loss in turn affects other biomarkers such as FPG, lipids, and blood pressure. The effect of weight loss on blood pressure is based on reference (4) above.

Healthcare System Model

The Archimedes Model includes a detailed model of the healthcare system. As described above in the overview of the Model, simulated healthcare providers follow protocols that are based on published U.S. guidelines for screening, diagnosing and treating diseases. The care provided by the simulated providers is adjusted to represent the average care provided in the United States today. That is, because guidelines are not applied perfectly in the real world, the Model is calibrated to account for healthcare providers who may not apply guidelines exactly as they are written, and to account for patients who may not adhere to prescribed tests, treatments, or follow-up care.

Guidelines Implemented in the Model

A literature search was performed to identify guidelines that have been issued by various organizations for each disease included in the Model. The main source for this was www.guidelines.gov. The guidelines were reviewed by in-house medical staff, as well as external advisers. A major consideration was to choose guidelines that are considered to be national in scope, such as guidelines issued by government-sponsored panels (e.g., ATP III guideline for cholesterol management), national-level professional societies (e.g., American Academy of Ophthalmology), and voluntary health organizations (e.g., American Heart Association and American Diabetes Association). The guidelines incorporated in the Archimedes Model and used by ARCHeS are in Table 13.

Table 13. Guidelines Implemented in the Model.

Disease	Sources
Hypertension	• The seventh report of the Joint National Committee on Prevention, Detection, Evaluation, and Treatment of High Blood Pressure (JNC-7), 2003.<br>• American Diabetes Association, Standards of medical care in diabetes—2009, Diabetes Care 2009; 32 Suppl 1:S13-61.<br>• Sacco RL et al. Guidelines for prevention of stroke in patients with ischemic stroke or transient ischemic attack, Stroke 2006; 37:577-617.

© 2011-2013

Disease	Sources
Dyslipidemia	• Executive summary of the third report of the National Cholesterol Education Program (NCEP) Expert Panel on Detection, Evaluation, and Treatment of High Blood Cholesterol in Adults (Adult Treatment Panel III), 2002.<br>• Grundy SM, Cleeman JI, Merz CN, Brewer HB Jr, Clark LT, Hunninghake DB, Pasternak RC, Smith SC Jr, Stone NJ; National Heart, Lung, and Blood Institute; American College of Cardiology Foundation; American Heart Association. Implications of recent clinical trials for the National Cholesterol Education Program Adult Treatment Panel III guidelines. Circulation. 2004; 110: 227–239.
Obesity	• Institute for Clinical Systems Improvement (ICSI): Health care guideline: prevention and management of obesity (mature adolescents and adults), 2009.
Diabetes	• American Diabetes Association, Standards of medical care in diabetes—2012, Diabetes Care 2012; 35 Suppl 1:S11-63.<br>• Inzucchi SE, et al. Management of hyperglycemia in type 2 diabetes: a patient-centered approach: position statement of the American Diabetes Association (ADA) and the European Association for the Study of Diabetes (EASD). Diabetes Care. 2012; 35:1364-79.
Diabetic retinopathy	• American Academy of Ophthalmology Retina Panel. Preferred Practice Pattern® Guidelines. Diabetic Retinopathy. San Francisco, CA: American Academy of Ophthalmology; 2008. (Available at: http://www.aao.org/ppp.)<br>• American Diabetes Association, Standards of medical care in diabetes—2012, Diabetes Care 2012; 35 Suppl 1:S11-63.<br>• Ferris F. Early Photocoagulation in Patients with Either Type I or Type II Diabetes. Tr. Am. Ophth. Soc. 1996; 94:505-531.
Diabetic neuropathy	• American Diabetes Association, Standards of medical care in diabetes—2012, Diabetes Care 2012; 32 Suppl 1:S11-63.<br>• Lipsky BA, et al. Diagnosis and Treatment of Diabetic Foot Infections. Clin Infect Dis. 2004. 39(7):885-910.<br>• Frykberg RG et al, Diabetic Foot Disorders. Journal of Foot and Ankle Surgery. 2006; 45(5):S1-S66.

Disease	Sources
Diabetic nephropathy	• American Diabetes Association, Standards of medical care in diabetes—2012, Diabetes Care 2012; 35 Suppl 1:S11-63.<br>• Levey AS, et al. National Kidney Foundation Practice Guidelines for Chronic Kidney Disease: Evaluation, Classification, and Stratification. Ann Intern Med. 2003; 139:137-147.<br>• Coresh J, et al. Prevalence of Chronic Kidney Disease in the United States. JAMA. 2007; 298(17):2038-204.<br>• National Kidney Foundation. I. Executive Summary, II. Clinical Practice Guidelines, III. Clinical Practice Recommendations. American Journal of Kidney Diseases. 2007; 49(2):S13-S153.
Stroke	• Adams HP et al. Guidelines for the early management of adults with ischemic stroke, Stroke 2007; 38(5):1655-1711.<br>• Sacco RL, et al. Guidelines for Prevention of Stroke in Patients With Ischemic Stroke or Transient Ischemic Attack. Stroke 2006, 37:577-617.<br>• Broderick J, et al. Guidelines for the Management of Spontaneous Intracerebral Hemorrhage in Adults : 2007 Update: A Guideline From the American Heart Association/American Stroke Association Stroke Council, High Blood Pressure Research Council, and the Quality of Care and Outcomes in Research Interdisciplinary Working Group. Stroke 2007, 38:2001-2023.
Atrial fibrillation	• Adams RJ et al. Update to the AHA/ASA recommendations for the prevention of stroke in patients with stroke and transient ischemic attack, Stroke 2008; 39:1647-1652.
Aspirin primary prevention	• U.S. Preventive Services Task Force. Aspirin for the Prevention of Cardiovascular Disease: U.S. Preventive Services Task Force Recommendation Statement. Ann Intern Med. 2009;150:396-404

Disease	Sources
Heart disease (includes MI, angina, CAD, and chest pain)	• ACC/AHA 2007 Guidelines for the management of patients with unstable angina/non-ST-elevation myocardial infarction, Circulation 2007; 116:803-877.<br>• ACC/AHA Guidelines for the management of patients with ST-elevation myocardial infarction, Circulation 2004; 110:e82-e293.<br>• ACC/AHA 2005 Guideline update for the diagnosis and management of chronic heart failure in the adult, Circulation 2005; 112:e154-e235.<br>• Gibbons RJ, Abrams J, Chatterjee K, et al. ACC/AHA 2002 guideline update for the management of patients with chronic stable angina: a report of the American College of Cardiology/American Heart Association Task Force on Practice Guidelines (Committee to Update the 1999 Guidelines for the Management of Patients with Chronic Stable Angina), 2002. (Available at www.acc.org/clinical/guidelines/stable/stable.pdf.)<br>• Institute for Clinical Systems Improvement (ICSI).Diagnosis and Treatment of Chest Pain and Acute Coronary Syndrome (ACS). Bloomington (MN): Institute for Clinical Systems Improvement (ICSI); 2008 Oct: 1-69.<br>• Antman EM, et al. 2007 Focused Update of the ACC/AHA 2004 Guidelines for the Management of Patients With ST-Elevation Myocardial Infarction. Circulation 2008, 117:296-329.<br>• Fraker TD, et al. 2007 Chronic Angina Focused Update of the ACC/AHA 2002 Guidelines for the Management of Patients With Chronic Stable Angina. Circulation 2007;116;2762-2772.<br>• Braunwald E. Epilogue: What Do Clinicians Expect From Imagers? J Am Coll Cardiol, 2006; 47:101-103.

© 2011-2013

Datasets Used for Model Calibration

Approximately 30 national datasets were surveyed to identify a collection of data that would most completely span the diseases and healthcare processes represented in the Archimedes Model. The goal was to identify sources of data that represent standard care within the general US population and also included information on subpopulations. The main datasets used to estimate the calibration targets are listed in Table 14.

For more information about the model calibration process, refer to the report "Care Processes: Calibration Methodology and Results," available at archimedesmodel.com/resource-center.

Table 14. Datasets Used for Model Calibration.

Dataset	Year	Survey Content	Survey Sample Design	Sample Size
NHANES<br><br>National Health And Nutrition Examination Survey	1999 - 2008	Chronic disease prevalence and conditions (including undiagnosed conditions), risk factors, diet and nutritional status, immunization status, infectious disease prevalence, health insurance, and measures of environmental exposures. Other topics addressed include hearing, vision, mental health, anemia, diabetes, cardiovascular disease, osteoporosis, obesity, oral health, mental health, and physical fitness.	Uses a stratified multistage probability sample, nationally representative of the US civilian non-institutionalized population.	Approximately 5,000 people are examined each year.
NAMCS<br><br>National Ambulatory Medical Care Survey	2006	Information is obtained on various aspects of office visits, including physician practice characteristics, patient characteristics, and other visit characteristics. Among the items collected are patient's age, gender, race, and ethnicity; patient's expressed reason for visiting the physician; intentionality of injury, if any; physician's diagnoses; diagnostic services ordered or provided; therapeutic services; ambulatory surgical procedures performed; medications; providers seen; visit disposition; time spent with physician; and expected source of payment.	National probability sample survey of visits to office-based physicians in the United States.	In 2004, 25,286 survey forms (each representing one physician-patient visit) were collected.

© 2011-2013

NHAMCS<br><br>National Hospital Ambulatory Care Survey	2006	The NHAMCS includes two files: emergency department (ED) visits and outpatient department (OPD) visits. Information is obtained on various aspects of patient visits, including patient, hospital, and visit characteristics. Among the items collected are patient's age, gender, race, and ethnicity; patient's expressed reason for visit; intentionality of injury, if any; physician's diagnoses; diagnostic services ordered or provided; procedures provided; medications; providers seen; visit disposition; immediacy with which patient should be seen; and expected source of payment. Items collected that are specific to the ED include mode of arrival, waiting time, duration of time in the ED, initial vital signs, and cause of injury.	National probability sample survey of visits to emergency departments (EDs) and outpatient departments (OPDs) of non-federal, short-stay, and general hospitals in the United States.	About 400 EDs and 225 OPDs participate each year. In 2004, 36,589 ED forms and 31,783 OPD forms were completed.
NHDS<br><br>National Hospital Discharge Survey	2006	Variables collected include age; gender; race; ethnicity; admission and discharge dates (length of stay); discharge status; source of payment; hospital size, ownership, and region; from one to seven diagnoses coded using the ICD-9-CM; and, from zero to four procedures using the ICD-9-CM.	Utilizes a three-stage national probability design that includes primary sampling units (PSUs), hospitals within the PSUs, and discharges within the hospitals.	Approximately 300,000 discharges are sampled each year from about 500 hospitals.

CMF<br><br>Compressed Mortality File	2006	The number of deaths, crude death rates or age-adjusted death rates can be obtained by place of residence (total US, census region, census division, state, and county), age group, race (years 1979-1998: White, Black, and Other; years 1999-present: American Indian or Alaska Native, Asian or Pacific Islander, Black or African American, and White), Hispanic origin (years 1979-1998: not available; years 1999-present: Hispanic or Latino, not Hispanic or Latino, not stated), gender, year of death, and underlying cause of death (years 1979-1998: 4-digit ICD-9 code and 72 cause-of-death recode; years 1999-present: 4-digit ICD-10 codes and 113 cause-of-death recode), and urbanization level of residence (2006 NCHS urban-rural classification scheme for counties).	Mortality information is collected by state registries and provided to the National Vital Statistics System. Underlying cause of death and demographic descriptors are indicated on the death certificates.	In 2006, among adults aged 20-84, 1,671,006 deaths were recorded in a population of 212,022,561.

Limitations of the Model

The Model has several limitations.

Population

The default population in the Model is the US population, as described by the NHANES survey. The Model can be configured to analyze other populations, based on either person-specific data or aggregated data from the new population of interest. This can be done either in consulting projects or through the ARCHeS interface.

Care processes

The default care processes in the Model are based on those currently used in the United States. Sources for the specific guidelines are described in the section the healthcare system model. The Model's implementation of these guidelines is calibrated as described in that section to match population characteristics and biomarkers, performance levels, and compliance levels seen in the United States. Care processes can be modified and customized to different settings in consulting projects, but at present cannot be modified through the ARCHeS interface.

Missing and inconsistent data

Setting up and calibrating the Model, specifically the population and care processes, requires using data from multiple sources, such as NHANES, NAMCS, NHAMCS, and NHDS. Because different data sources are derived from different populations and use different definitions and methods, their results are often

© 2011-2013

not entirely consistent, and judgments have to be made about the most appropriate target to use for calibration and the range of uncertainty to explore.

Diseases, interventions, and outcomes

While the Archimedes Model is a single integrated model that includes multiple conditions, there are many diseases that are not in the Model. This document describes only the diseases, interventions, and outcomes included in the Model that are available via the ARCHeS interface. Additional diseases, interventions, and outcomes are available in the Model for consulting projects.

Standard care remains constant

When calculating outcomes and the effects of interventions in the future, the Model assumes that, in general, care will be delivered at the same level as today. The exceptions would be whatever interventions are being studied in any particular analysis.

Adults only

Currently, the Archimedes Model creates populations of age 20-85. It does not include pediatric conditions, interventions, or outcomes.

Population remains the same through the simulation period

In ARCHeS, the default is that a simulation includes only people who are in the population at the simulation start date. New individuals do not enter the population after the start of the simulation. People in the initial population leave the simulation only when they die. The Model can be configured to incorporate people entering and leaving the simulation such as might happen with individuals moving in and out of health plans. However, this must be done through a consulting project; it cannot be set up in the ARCHeS interface.

No patient interactions

The Model does not yet allow for interactions between patients. Thus it is currently not able to address the spread of infections in a population.

No competing resources

The Model tracks the use of resources at a high level of detail for each individual, and these data can be combined to determine the total amount of resources used. However, the Model assumes that the amount of resources is unlimited. It does not explicitly include resource shortages, competition for resources, queues, or bottlenecks.

Disease-based

The Archimedes Model is built up from the underlying physiology and pathophysiology that causes diseases. It is designed to address questions that relate to the incidence and progression of diseases, and their management. Because only certain diseases are in the Model, the Model is not designed to answer system-wide questions or general questions about the delivery or financing of a total healthcare system. For example, it is not designed to answer questions about healthcare workforces or facilities, or very general policies about coverage. Questions of this nature can be addressed but need to be

© 2011-2013

narrowed so as to apply to the individual diseases that are in the Model. For example, because diabetes is in the Model, the Model could be used to address the need for diabetes specialists in the future and how that is affected by trends in obesity and its management.

Socioeconomic status and insurance

Currently, the Model does not include socioeconomic status or insurance coverage. If there are descriptive data about a subpopulation that has a particular socioeconomic or insurance coverage status, then those descriptive data can be used to create a simulated population that matches the subpopulation of interest. This would need to be done through a consulting project; it cannot be set up in the ARCHeS interface.

Unknown or unmeasured covariates

To the greatest extent permitted by the available data, the Model incorporates information about factors that affect the incidence and progression of diseases – “covariates.” However, in any population, whether it is based on geography or the inclusion/exclusion criteria of a clinical trial, there are inevitably covariates that affect the outcomes of interest that are unknown, unmeasured, or unreported. It is not possible to incorporate the effects of missing or unmeasured covariates. For this reason, there will always be uncertainty about the Model’s results for any new population. This limitation of the Model will gradually decrease as future epidemiological studies identify new covariates.

Unknown risks and side effects

The Model does not include any outcomes or the effects of any interventions that have not been observed and reported. If an intervention has been shown to affect a biomarker that is in the Model, then the Model can be used to predict implications of the effect on that biomarker on longer-term health outcomes. For example, if a side effect of a glucose control agent is an increase in LDL cholesterol, then the effects on LDL cholesterol will be incorporated in the Model’s calculations of the effects of the glucose control agent on cardiovascular outcomes.

Acknowledgements

The Model has been evolving since originally conceived by Dr. Eddy and first given life by Dr. Schlesinger so many years ago. Many scientists, engineers, and physicians have been involved in crafting the current Model. We are indebted to Kathleen Thompson for her work on the early drafts of this document and for leading the modeling effort in the past four years; to the cancer modeling team, Tuan Dinh and Joyce Noah-Vanhoucke; to the heart modeling team, Ian Blumenfeld and Adam Guetz; to the diabetes modeling team, Stuart Samuel and Kenny Shum; to the healthcare modeling team, Julia Dziuba and Swathi Guda; to Carl Anderson, Andrew Van Herick, Helene Grossman, Andy Schuetz, Roshan Shah, and Amory Schlender for their contributions; to Barbara Peskin, Andrea Kress, Jennifer Burton, and Patrick van der Valk for supporting the efforts to produce this version of the Model and this document; to Brian Zuzga and Paul Jasper for providing leadership and direction in architectural integrity and coding standards; to Katrina Montinola for her leadership and support in realizing the science embodied in the

© 2011-2013

Model in “military grade” software; and finally, to all the former Archimedeans on whose shoulders we stand.

Sources of Funding

Archimedes, Inc. is a for-profit healthcare modeling company owned by Kaiser Permanente. Kaiser Health Plans is a not-for-profit company.

Primary Contributors

- Kaiser Permanente
- Robert Wood Johnson Foundation
- American Diabetes Association

© 2011-2013

ARCHIMEDES
a KAISER PERMANENTE Innovation
123 Mission Street, 11th floor
San Francisco, CA 94105, USA
www.archimedesmodel.com
info@archimedesmodel.com
+1 415 490 0400

© 2011-2013
