# Supplementary material for: Simulating the Impact of Improved Cardiovascular Risk Interventions on Clinical and Economic Outcomes in Russia
Source: PLoS One. 2014 Aug 20;9(8):e103280. doi: 10.1371/journal.pone.0103280 (PMC4139197; doi:10.1371/journal.pone.0103280)
Supplement: File S1 — Appendix S1: Description of the Archimedes Model. Appendix S2: Validation Methodology and Results. Appendix S3: Age Standardized Mortality in Russian Federation and Yaroslavl Region in 2010. Appendix S4: Incidence of cardiovascular diseases according to Yaroslavl Region's department of health (DoH) statistics in 2010. (ZIP) [file pone.0103280.s002.zip › File S1/Appendix S3 Mortality morbidity for calibration Russia 2010.doc]

**Age standardized mortality in Russian Federation and Yaroslavl Region in 2010**

*(STANDARDIZATION performed and data provided by russian state scientific research center of preventive medicine based on rosstat tables)*

**Total population (European standard)**

| **Mortality cause** | **Russian Federation** | | | **Yaroslavl Region** | | |
| --- | --- | --- | --- | --- | --- | --- |
|  | **Men** | **Women** | **All** | **Men** | **Women** | **All** |
| **All causes (total mortality)** | **1782,8** | **881,4** | **1247,2** | **1946,3** | **860,2** | **1276** |
| **Cardiovascular mortality** | **916,8** | **518** | **673,9** | **1039,5** | **516,2** | **706,3** |
| **Coronary Heart Disease Mortality** | **501** | **254,6** | **348,8** | **588,8** | **245,5** | **367,1** |
| **Cerebrovascular Diseases Mortality** | **261,9** | **184,8** | **214,9** | **303,1** | **208,1** | **241,6** |

**Ages 25-64 (European standard**)

| **Mortality cause** | **Russian Federation** | | | **Yaroslavl Region** | | |
| --- | --- | --- | --- | --- | --- | --- |
|  | **Men** | **Women** | **All** | **Men** | **Women** | **All** |
| **All causes (total mortality)** | **1443,9** | **475,8** | **914,9** | **1645,4** | **478,2** | **955,5** |
| **Cardiovascular mortality** | **560,4** | **171,2** | **344,3** | **658,5** | **165,1** | **379,3** |
| **Coronary Heart Disease Mortality** | **303,4** | **73,7** | **175,2** | **389,7** | **77,3** | **212,4** |
| **Cerebrovascular Diseases Mortality** | **113,9** | **49,2** | **77,6** | **90,1** | **30,3** | **55,5** |

**Prevalence and incidence of cardiovascular diseases in adult population of the Russian Federation and Yaroslavl Region (2010)**

| **Region** | **Total number of patients with registered disease (prevalence)** | | **Including those with first time established diagnosis in this year (incidence)** | |
| --- | --- | --- | --- | --- |
|  | **Total** | **Per 100 000** | **Total** | **Per 100 000** |
|  | **Cardiovascular diseases (all)** | | | |
| **Russian Federation** | 31714079 | 27355,2 | 3468397 | 2991,7 |
| **Yaroslavl Region** | 271470 | 24786,7 | 32276 | 2946,9 |
|  | **Hypertensive diseases** | | | |
| **Russian Federation** | 11739820 | 10126,3 | 855198 | 731,7 |
| **Yaroslavl Region** | 100485 | 9174,5 | 5165 | 471,6 |
|  | **Coronary heart diseases** | | | |
| **Russian Federation** | 7373730 | 6360,3 | 707151 | 610,0 |
| **Yaroslavl Region** | 53477 | 4882,5 | 4962 | 453,0 |
|  | **Cerebrovascular diseases** | | | |
| **Russian Federation** | 7024377 | 6058,9 | 851179 | 734,2 |
| **Yaroslavl Region** | 78110 | 7131,6 | 9765 | 891,6 |
