# Supplementary material for: Simulating the Impact of Improved Cardiovascular Risk Interventions on Clinical and Economic Outcomes in Russia
Source: PLoS One. 2014 Aug 20;9(8):e103280. doi: 10.1371/journal.pone.0103280 (PMC4139197; doi:10.1371/journal.pone.0103280)
Supplement: File S1 — Appendix S1: Description of the Archimedes Model. Appendix S2: Validation Methodology and Results. Appendix S3: Age Standardized Mortality in Russian Federation and Yaroslavl Region in 2010. Appendix S4: Incidence of cardiovascular diseases according to Yaroslavl Region's department of health (DoH) statistics in 2010. (ZIP) [file pone.0103280.s002.zip › File S1/Appendix S4 CV diseases Yaroslavl Region.doc]

**Incidence of cardiovascular diseases according to Yaroslavl Region’s department of health (DoH) statistics in 2010**

*(DATA provided to Novartis in 2011,* population = 1 306 320 people)

**TOTAL number of DIAGNOSED events in Yaroslavl** region in 2010

| **Coronary heart diseases** (same number as a above) | 4962 |
| --- | --- |
| Acute myocardial infarction, all cases (not seen above) | 2627 |
| Primary myocardial infarction (first event) | 1992 |
| Secondary myocardial infarction (second, etc. event) | 635 |
| **Cerebrovascular diseases, all cases** (same number as a above) | 9765 |
| Stroke, all cases (not seen above) | 5031 |
| Ischemic Stroke | 3420 |
| Hemorrhagic Stroke | 556 |
| Subarachnoid bleeding | 102 |

**N.B.** Please note the difference in Stroke vs cerebrovascular diseases. Approx. half of incidence of cerebrovascular diseases is due to verified strokes, the rest are difficult to classify (we have a so-called diagnosis of “dyscirculation encephalopathy” and TIAs are not really recorded here and mostly are under stroke classification). The same applies to mortality – it is an aggregate of stroke and non-stroke cerebrovascular diseases mortality. Approximately half (like with incidence) of mortality cases are established diagnoses of stroke and the other half – “other” cerebrovascular disorders, where the diagnosis was not established at time of death, but the patient had a history of cerebrovascular diseases (data provided in 2011 from Region’s DoH):

| **CV MORTALITY CAUSES** *(ABSOLUTE NUMBER OF CASES FOR 2010):* | **TOTAL** |
| --- | --- |
| **Chronic rheumatic diseases** | **49** |
| Hypertensive heart disease | 116 |
| Hypertensive kidney disease | 6 |
| Hypertensive heart and kidney disease | 12 |
| Other forms of Hypertensive disease | 1 |
| **Hypertensive disease (total)** | **135** |
| Acute myocardial infarction | 549 |
| Secondary infarction | 183 |
| Atherosclerosis heart disease | 4317 |
| Other forms of chronic ischemia | 1457 |
| Other forms of acute ischemia | 127 |
| **Coronary heart disease (total)** | **6633** |
| **Pulmonary heart** | **5** |
| **Other heart diseases** | **521** |
| Subarachnoid bleeding | 53 |
| Hemorrhagic Stroke | 556 |
| Ischemic Stroke | 1361 |
| Stroke unspecified | 740 |
| Other cerebrovascular diseases | 2522 |
| **Cerebrovascular diseases (total)** | **5232** |
| **Atherosclerosis** | **125** |
| **Other diseases of arteries and arterioles** | **95** |
| **Phlebitis, thrombophlebitis** | **53** |
| **Other diseases of lymphatic vessels** | **7** |
| **TOTAL CV MORTALITY CASES IN 2010** | **12855** |
